# Supplementary material for: PRMT5 activates AKT via methylation to promote tumor metastasis
Source: Nat Commun. 2022 Jul 8;13:3955. doi: 10.1038/s41467-022-31645-1 (PMC9270419; doi:10.1038/s41467-022-31645-1)
Supplement: Supplementary file 1 — Supplementary Information [file 41467_2022_31645_MOESM1_ESM.pdf]

Table S1. Antibodies and antibody/lipids-coated beads used in this study

Name	Vendor	Catalog Number
AKT	Cell Signaling Technology	9272
AKT1	Cell Signaling Technology	2938
AKT2	Cell Signaling Technology	3063
AKT3	Cell Signaling Technology	14982
phospho-AKT Ser 473	Cell Signaling Technology	4060
phospho-Akt Thr308	Cell Signaling Technology	13038
phospho-AKT1 Ser 473	Cell Signaling Technology	9018
phospho-AKT2 Ser 474	Cell Signaling Technology	8599
Symmetric Di-Methyl Arginine Motif [sdme-RG] MultiMab™ Rabbit mAb	Cell Signaling Technology	13222
Anti-dimethyl-arginine antibody, symmetric, (SYM10)	Sigma-Aldrich	07-412
DNA-PK	Cell Signaling Technology	38168
EGFR	Cell Signaling Technology	4267
phospho-EGFR Tyr 1068	Cell Signaling Technology	3777
ErbB3	Cell Signaling Technology	12708
p-ErbB3 Tyr1289	Cell Signaling Technology	4791
FGFR4	Cell Signaling Technology	8562
VGFR2	Cell Signaling Technology	9698
p-VGFR2 Tyr 1059	Cell Signaling Technology	3817
IGF-1 Receptor beta	Cell Signaling Technology	9750
phospho-IGF-1 beta Tyr1316	Cell Signaling Technology	28897
GSK-3α	Cell Signaling Technology	4818
GSK-3β	Cell Signaling Technology	12456
phospho-GSK-3α Ser21	Cell Signaling Technology	9316
phospho-GSK-3β Ser9	Cell Signaling Technology	5558
Flag	Sigma-Aldrich	F7425
anti-HA-Peroxidase	Sigma-Aldrich	12013819001
phospho-(Ser/Thr) Phe	Cell Signaling Technology	9631
phospho-Tyr-1000	Cell Signaling Technology	8954
p75NTR	Cell Signaling Technology	8238
PDK1	Cell Signaling Technology	3062
PP2A A subunit	Cell Signaling Technology	2041T
PP2A B subunit	Cell Signaling Technology	2290T
PP2A C subunit	Cell Signaling Technology	2259T
PRMT5	Santa Cruz Biotechnology	sc-376937
PTEN	Cell Signaling Technology	9188
Rictor	Cell Signaling Technology	9476
Phospho-Rictor Thr 1135	Cell Signaling Technology	3806
PRMT9	Bethyl Laboratories	A304-189A
ZEB1	Cell Signaling Technology	70512

SNAIL	Cell Signaling Technology	3879
TWIST1	Cell Signaling Technology	69366
SIN1	Cell Signaling Technology	12860
Mouse anti-rabbit IgG-HRP	Santa Cruz Biotechnology	sc-2357
Mouse IgGκ BP-HRP	Santa Cruz Biotechnology	sc-516102
Anti-rabbit IgG, HRP-linked antibody	Cell Signaling Technology	7074
Normal rabbit IgG	Cell Signaling Technology	2729
Alexa Fluor 488-conjugated goat anti-rabbit	ThermoFisher	A32731
Alexa Fluor 594-conjugated goat anti-mouse	ThermoFisher	A11032
Mouse monoclonal anti-HA antibody-coated magnetic beads	ThermoFisher	88836
Phosphatidylserine agarose beads	Echelon Biosciences	P-B0PS

Table S2. Mouse Blood Chemistry Test

**Test Item**	**Samples**					
	**Vehicle**			**GSK595**		
ALB	3.7	4.1	3.5	3.5	3.1	3.7
ALP	37	29	26	61	30	27
ALT	52	59	44	25	30	27
AMY	860	1216	1564	798	730	795
TBIL	0.4	102	0.9	0.5	1.3	0.5
BUN	20	32	53	16	30	15
CA	10.9	11.4	10.4	10.5	10.7	10.9
PHOS	8	8	10.6	9.8	8.9	7.7
CRE	0.2	0.2	0.5	0.5	<0.2	<0.2
GLU	260	185	163	171	200	165
TP	5.4	5.6	4.4	5.1	4.9	4.9
GLOB	1.6	1.5	0.9	1.6	1.8	1.2

ALB: albumin; ALP: alkaline phosphatase; ALT: alanine aminotransferase; AMY: amylase; TBIL: total bilirubin test; BUN: blood urea nitrogen; CA: calcium; PHOS: phosphorus; CRE: creatinine; GLU: glucose; TP: total protein; GLOB: globulin

Table S3. Bioluminescent intensity of xenografts prior to and at the end of treatment

	Bioluminescent Intensity (total flux, pixel/second)			
	DMSO		GSK595	
**Cell lines**	**Day 0**	**Day 14**	**Day 0**	**Day 14**
**CHLA20-iRFP720-LUC**	1.15E+07	2.44E+09	3.05E+07	1.25E+08
	6.95E+07	3.07E+09	9.75E+07	2.36E+08
	9.43E+06	9.70E+08	1.96E+07	1.23E+08
	2.71E+07	1.30E+09	1.66E+07	1.26E+08
	3.41E+07	1.03E+09	3.62E+07	1.39E+08
	1.12E+08	5.06E+09	6.02E+07	3.49E+09
	1.62E+07	7.73E+09	2.55E+07	5.10E+08
	6.74E+07	9.01E+09	2.12E+07	2.01E+08
	7.11E+07	4.20E+09	3.13E+07	5.54E+08
			4.66E+07	2.90E+08
**NGP-iRFP720-LUC**	3.45E+07	1.10E+09	2.24E+07	5.28E+08
	2.18E+07	9.34E+08	9.00E+06	1.09E+08
	9.15E+06	1.54E+09	8.23E+07	2.76E+08
	5.35E+07	1.38E+09	1.62E+07	4.60E+08
	3.03E+08	7.08E+09	1.24E+08	2.04E+09
	9.75E+07	6.28E+09	7.99E+07	5.65E+08
	1.26+08	4.21E+09	1.22E+07	2.18E+08
			2.25E+07	8.60E+08
			7.76E+06	1.02E+08

Supplementary Figure 1

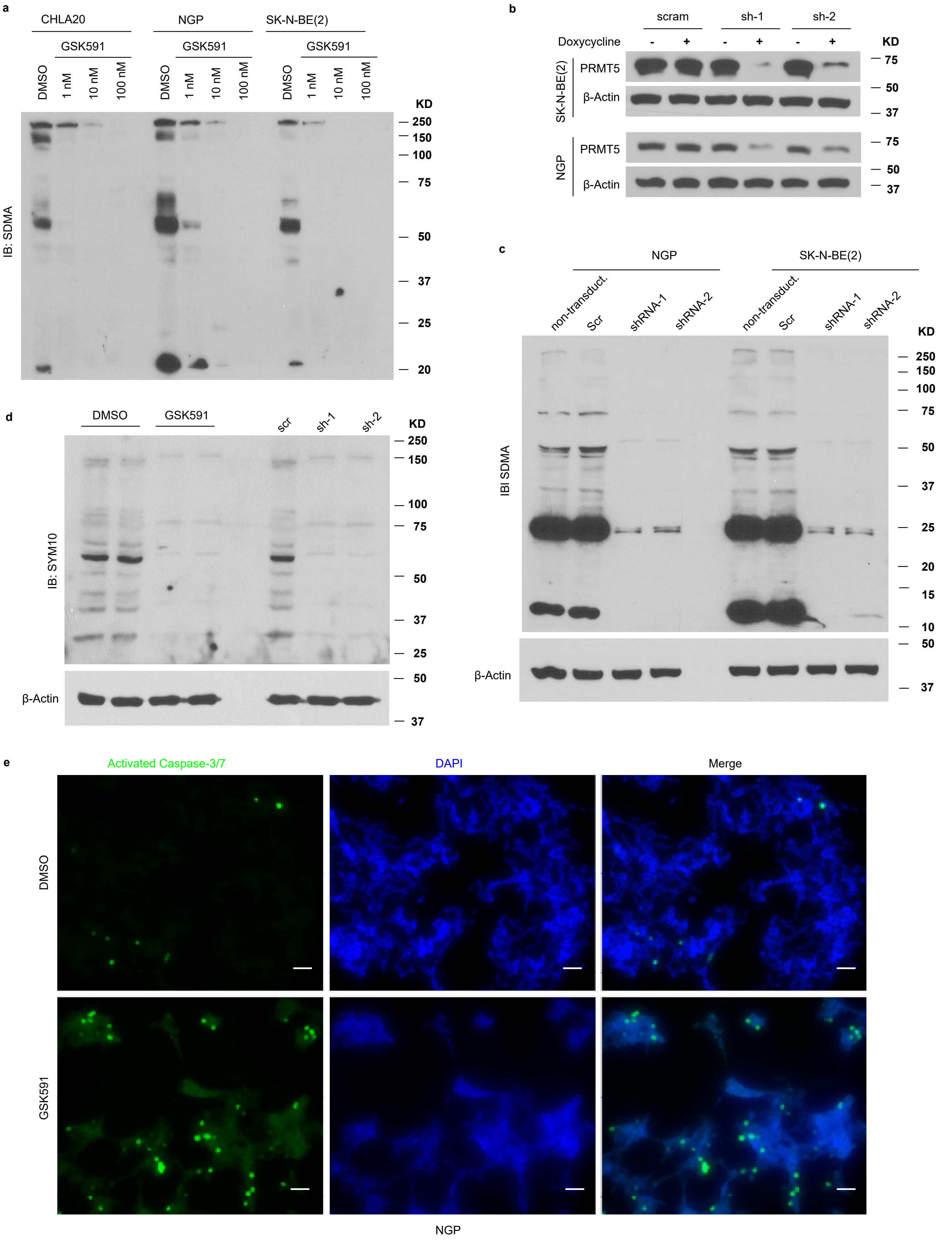

Fig. S1. PRMT5 inhibition decreases SDMA and induces apoptosis in neuroblastoma cells. a, Immunoblotting of SDMA with Cell Signaling antibody in neuroblastoma cells treated with DMSO or increasing doses of GSK591. **b**, PRMT5 knockdown efficiency was detected by Western blotting in scramble or PRMT5 knockdown cells in the absence or presence of doxycycline. **c**, SDMA in control and PRMT5 knockdown cells. **d**, Western blotting analysis of SDMA using an additional antibody (SYM10) against SDMA in CHLA 20 cells treated with DMSO or GSK591, and SK-N-BE(2) cells transduced with scramble or two shRNAs targeting PRMT5. **e**, Caspase-3/7 staining in NGP cells treated with DMSO or GSK591. Scale bars, 100 μm. All the results shown here were representative of three independent experiments. Uncropped immunoblots were provided in Source Data file.

Supplementary Figure 2

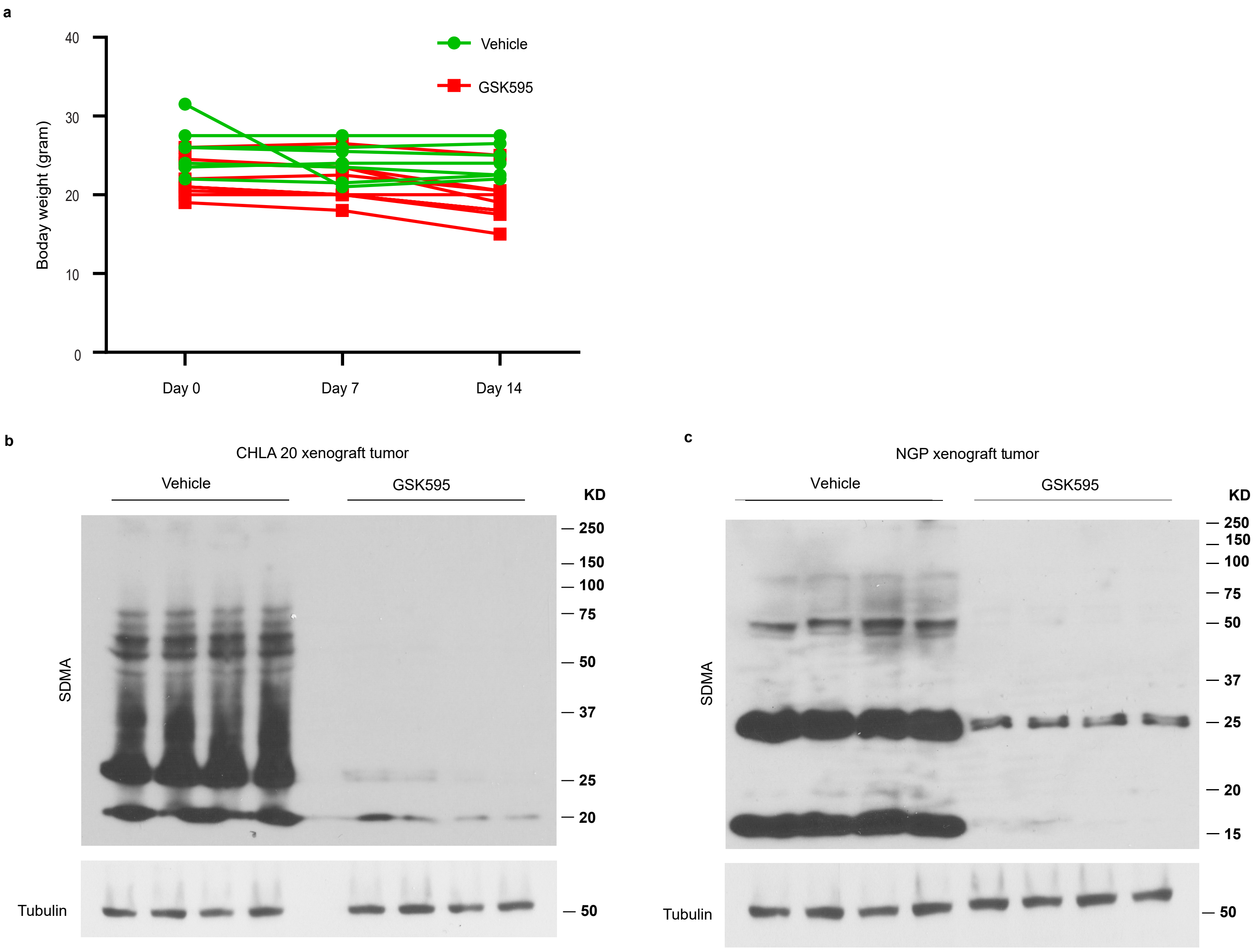

Fig. S2. GSK595 treatment shows efficacy *in vivo*. a, Weekly bodyweight of mice used in this study (n=7-9, Vehicle=7, GSK595=9). Immunoblots of SDMA in CHLA20 (**b**) (n=4) and NGP (**c**) (n=4) xenograft tumors (equal loading control seen in Fig. 3**e**). Uncropped immunoblots were provided in Source Data file.

Supplementary Figure 3

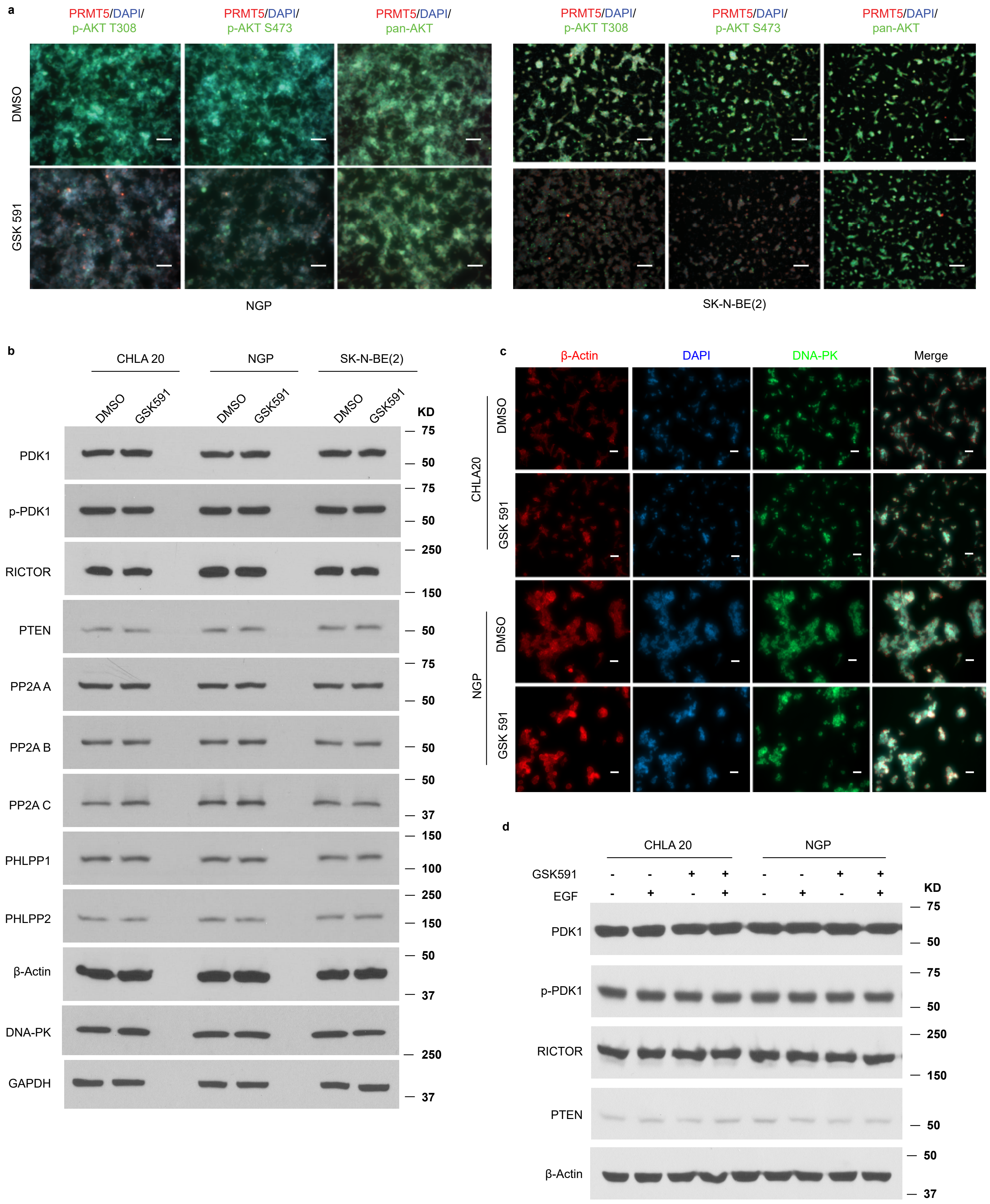

Supplementary Figure 3 continued

e

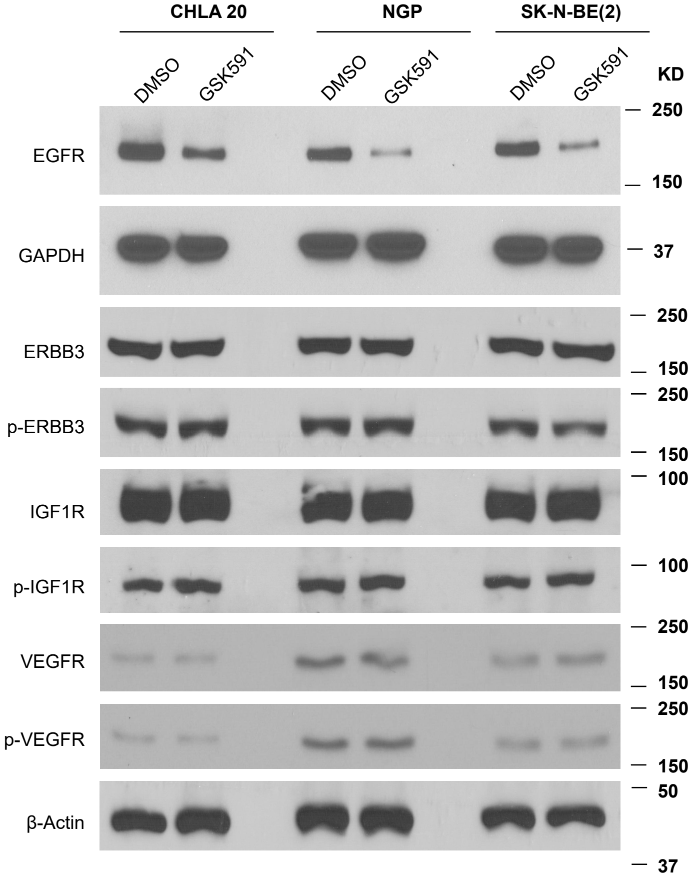

f

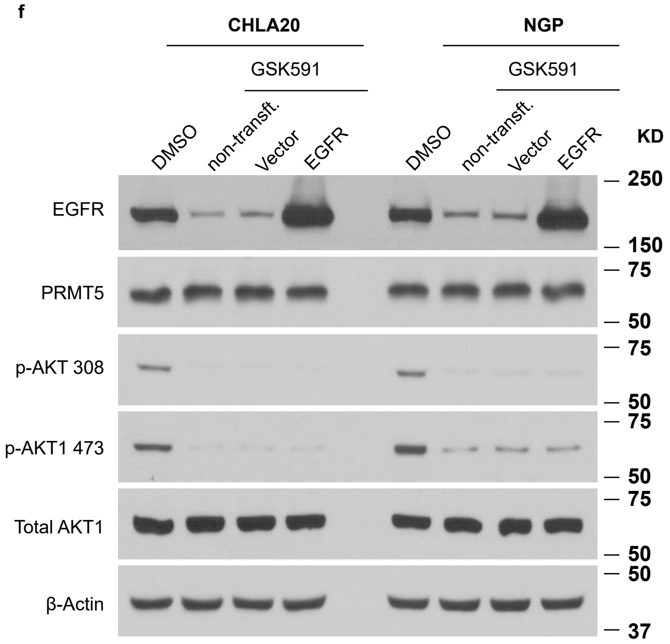

g

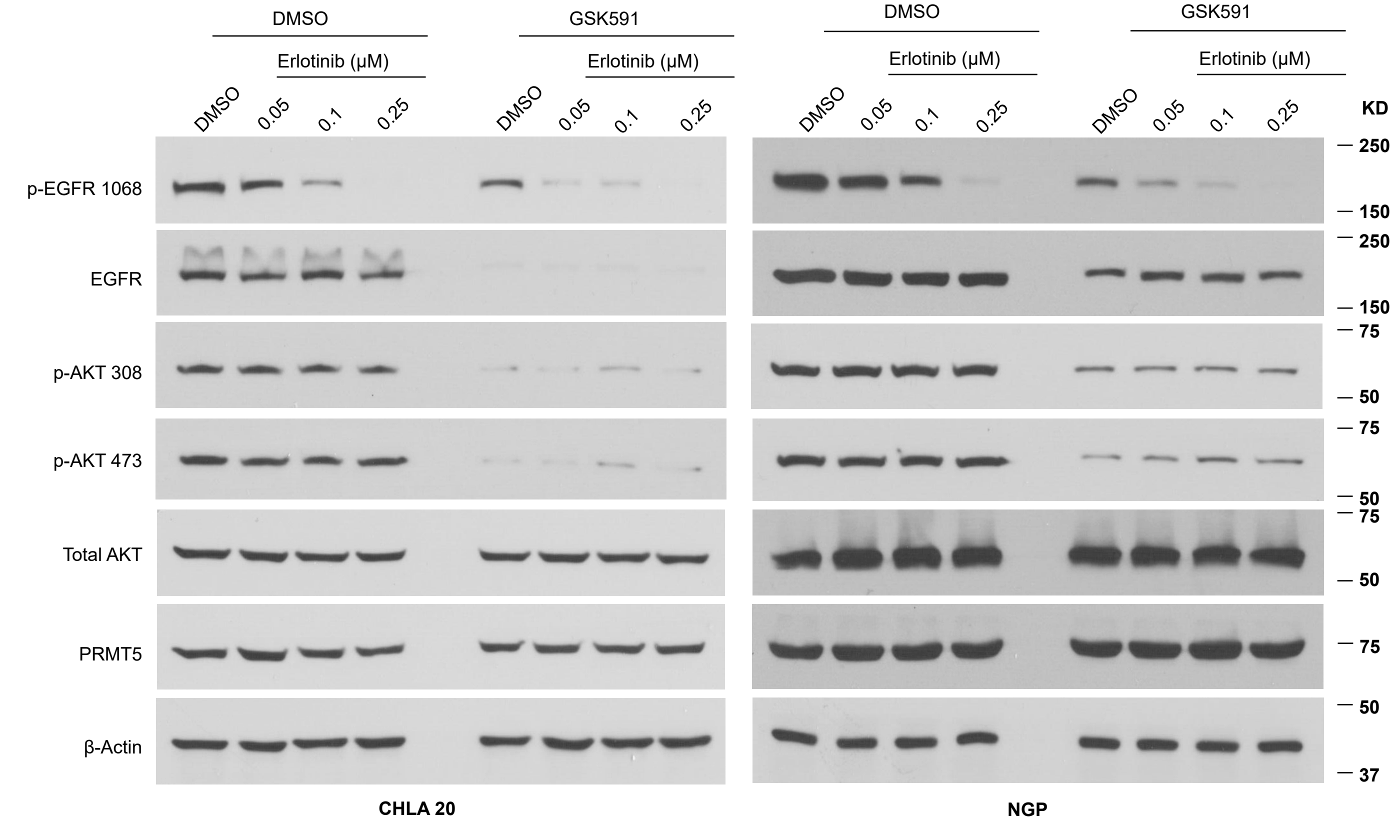

Fig. S3. Screening the impact of PRMT5 inhibition on known upstream regulators of AKT activation. a, Immunofluorescence showing the levels of PRMT5, phosphorylated AKT, and total AKT in NGP (left) and SK-N-BE(2) (right) cells treated with DMSO or GS591. Scale bars, 100 μm. **b**, The expression or phosphorylation of indicated kinases or phosphatases involved in the regulation of AKT activation in neuroblastoma cells under GSK591 treatment. **c**, Immunofluorescence showing the expression of DNA-PKs in CHLA20 and NGP cells treated with DMSO or GS591. Scale bars, 100 μm. **d**, Western blots of PDK1, phospho-PDK1, Rictor and PTEN in CHLA20 and NGP cells treated with DMSO or GSK591 with or without EGF stimulation. **e**, Immunoblots showing the protein levels or phosphorylation of RTKs not affected by GSK591 treatment. **f**, Immunoblots showing the levels of, EGFR, p-AKT Thr308, p-AKT Ser473, and PRMT5 in cells treated with DMSO or GSK591 followed by indicated doses of Erlotinib. **g,** The expression of EGFR, PRMT5, and phosphorylation of AKT1 examined by Western blot in cells either non-transfected or transfected with vector and EGFR, respectively. **a**, **c**, **d**, **f**, and **g**, representative results from three independent experiments. **b** and **e**, representative results from two independent experiments. Uncropped immunoblots were provided in Source Data file.

Supplementary Figure 4

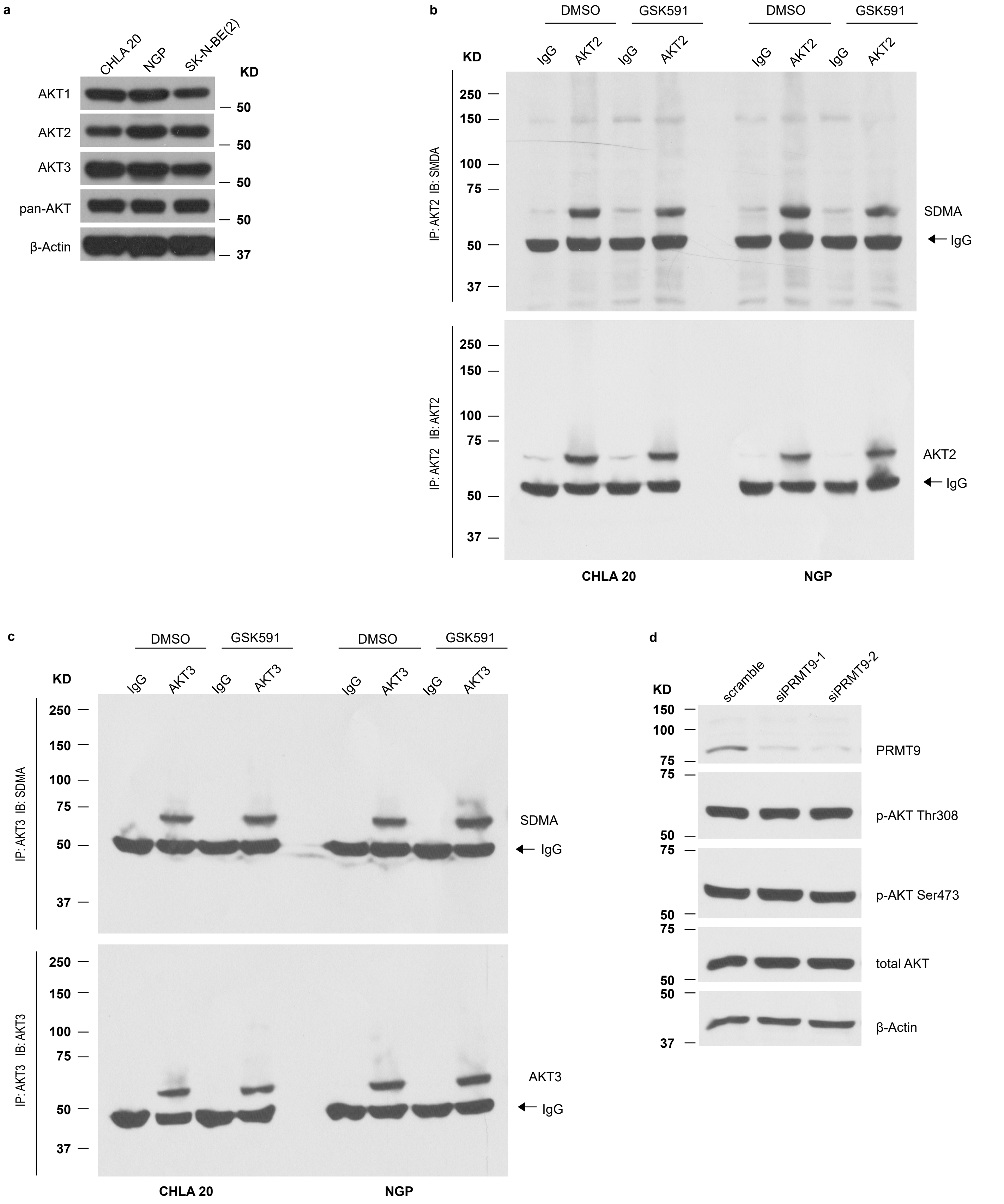

Fig. S4. GSK591 treatment does not affect SDMA on AKT2/3. **a**, The expression of AKT isoforms in neuroblastoma cell lines. **b**, Analyzing AKT2 SDMA in CHLA20 and NGP cells treated with DMSO or GSK591. **c**, Western blots of SDMA on AKT3 in CHLA20 and NGP cells treated with DMSO or GSK591. **d**, Western blots showing levels of phosphorylated AKT in control or PRMT9 knockdown cells. **a-c**, representative results from three independent experiments. **d**, representative results from two independent experiments. Uncropped immunoblots were provided in Source Data file.

Supplementary Figure 5

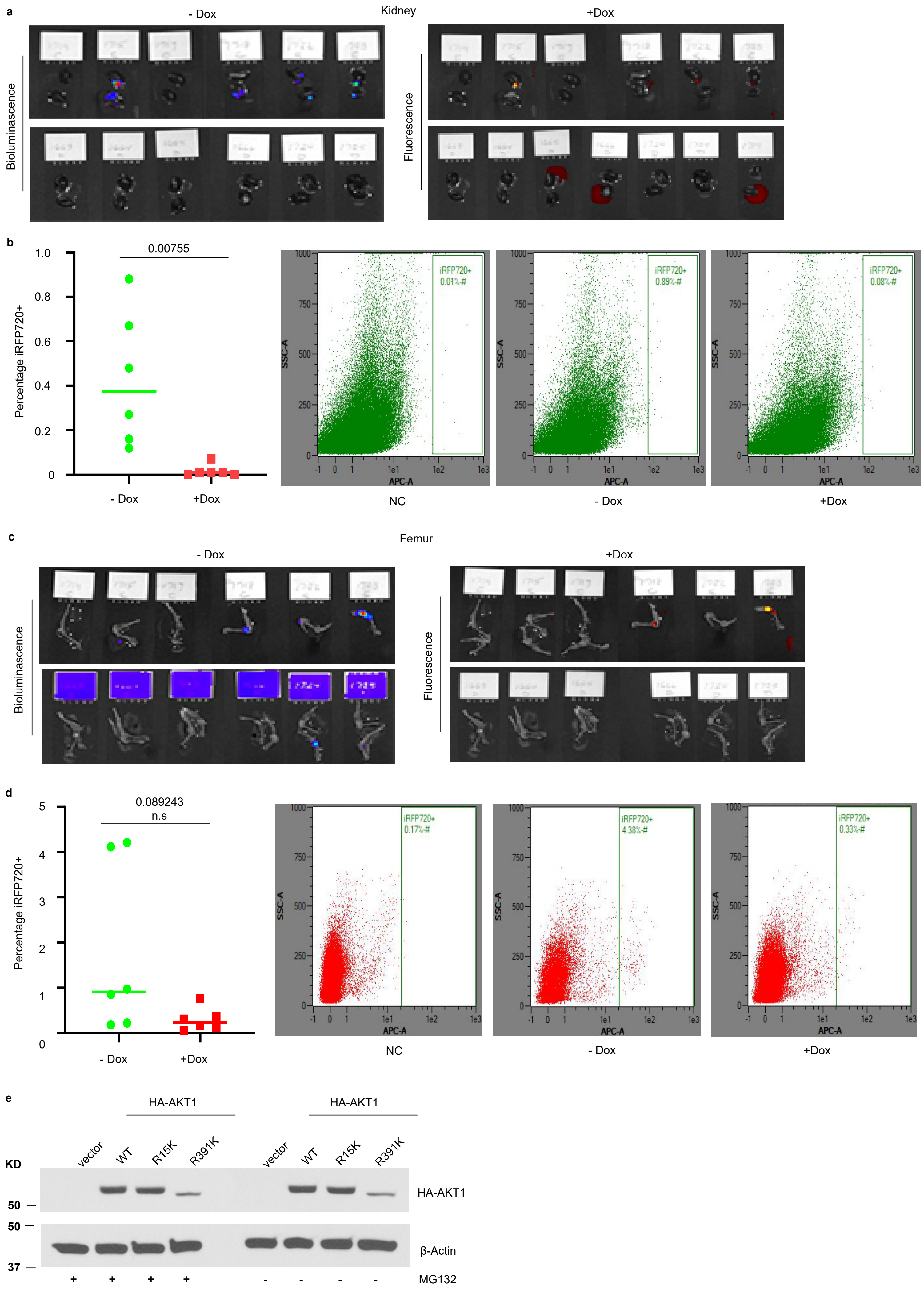

Fig. S5. PRMT5 depletion reduces tumor metastasis in the kidney. a, Bioluminescent (upper) and fluorescent imaging (lower) of kidneys harvested from mice described in Fig. 7a. **b**, FACS analysis of iRFP720+ human neuroblastoma cells in the kidneys (n=6). **c**, Bioluminescent (upper) and fluorescent imaging (lower) of femurs harvested from mice described in Fig. 7a. **d**, FACS analysis of iRFP720+ human neuroblastoma cells in the bone marrow isolated from the femur (n=6). **e**, Western blot showing the expression of HA-AKT1 wild type, R15K, and R391K in the absence and presence of MG132. The Western blotting results shown here were representative of two independent experiments. Uncropped immunoblots were provided in Source Data file. Uncropped immunoblots were provided in Source Data file. *p* values are calculated by two-tailed unpaired Student's *t* test using Microsoft Excel. Error bars represent SD, n.s, not significant.

Supplementary Figure 6. Uncropped Western blots

Fig. 1f

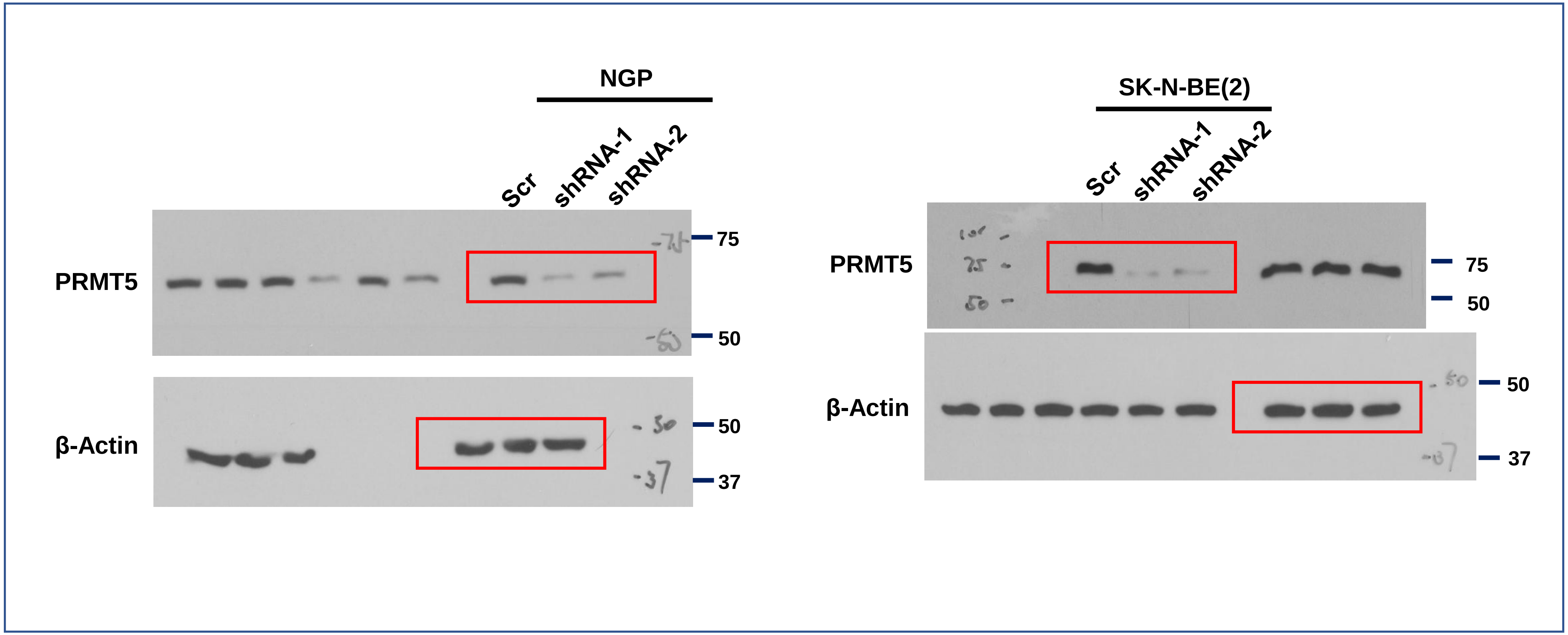

Fig. 1g

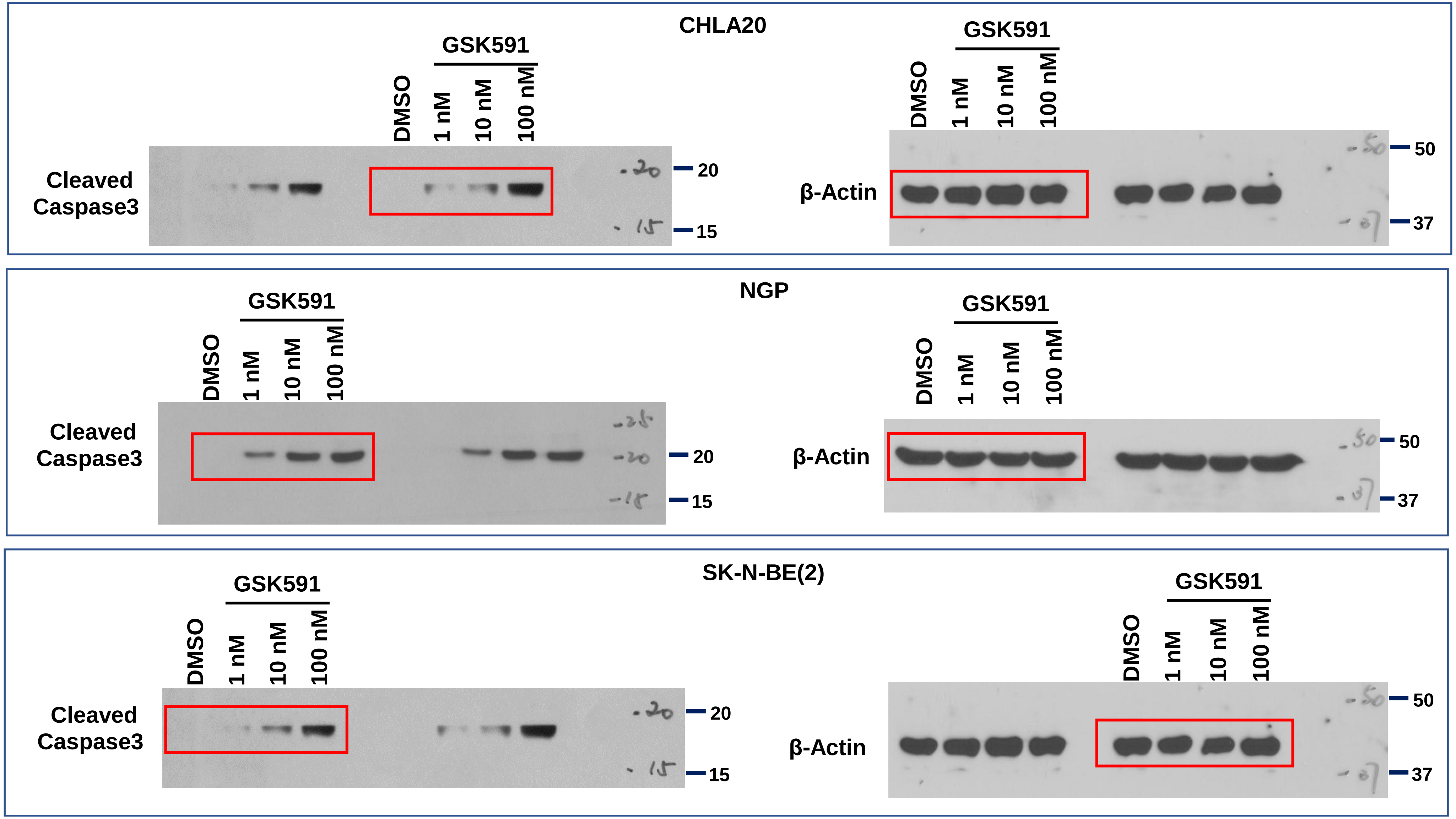

CHLA20
GSK591
DMSO
1 nM
10 nM
100 nM
Cleaved Caspase3
20
15
β-Actin
50
37
NGP
SK-N-BE(2)

Fig. 3b

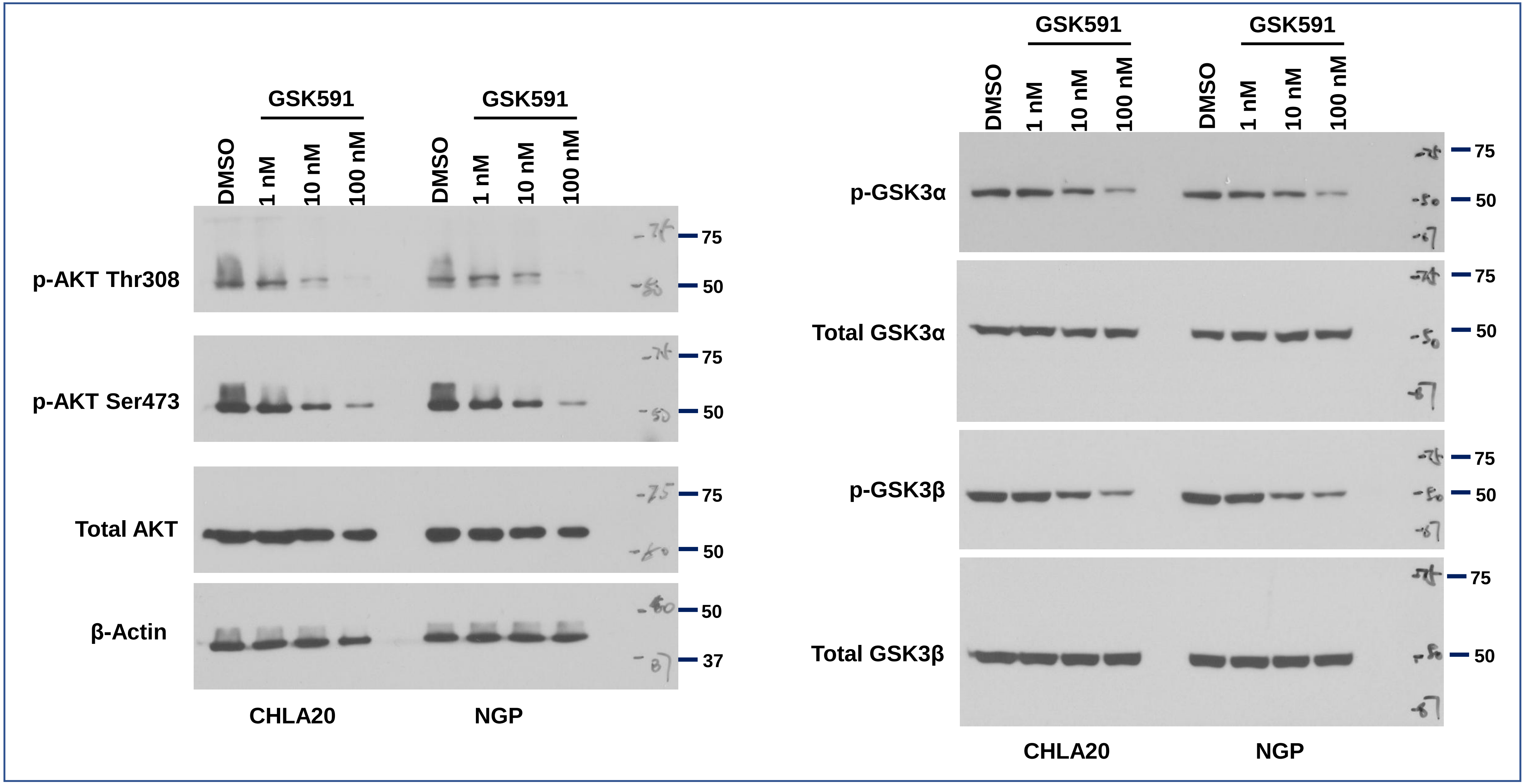
GSK591
DMSO
1 nM
10 nM
100 nM
p-AKT Thr308
p-AKT Ser473
Total AKT
β-Actin
75
50
37
CHLA20
NGP
p-GSK3α
Total GSK3α
p-GSK3β
Total GSK3β

Fig. 3c left panel

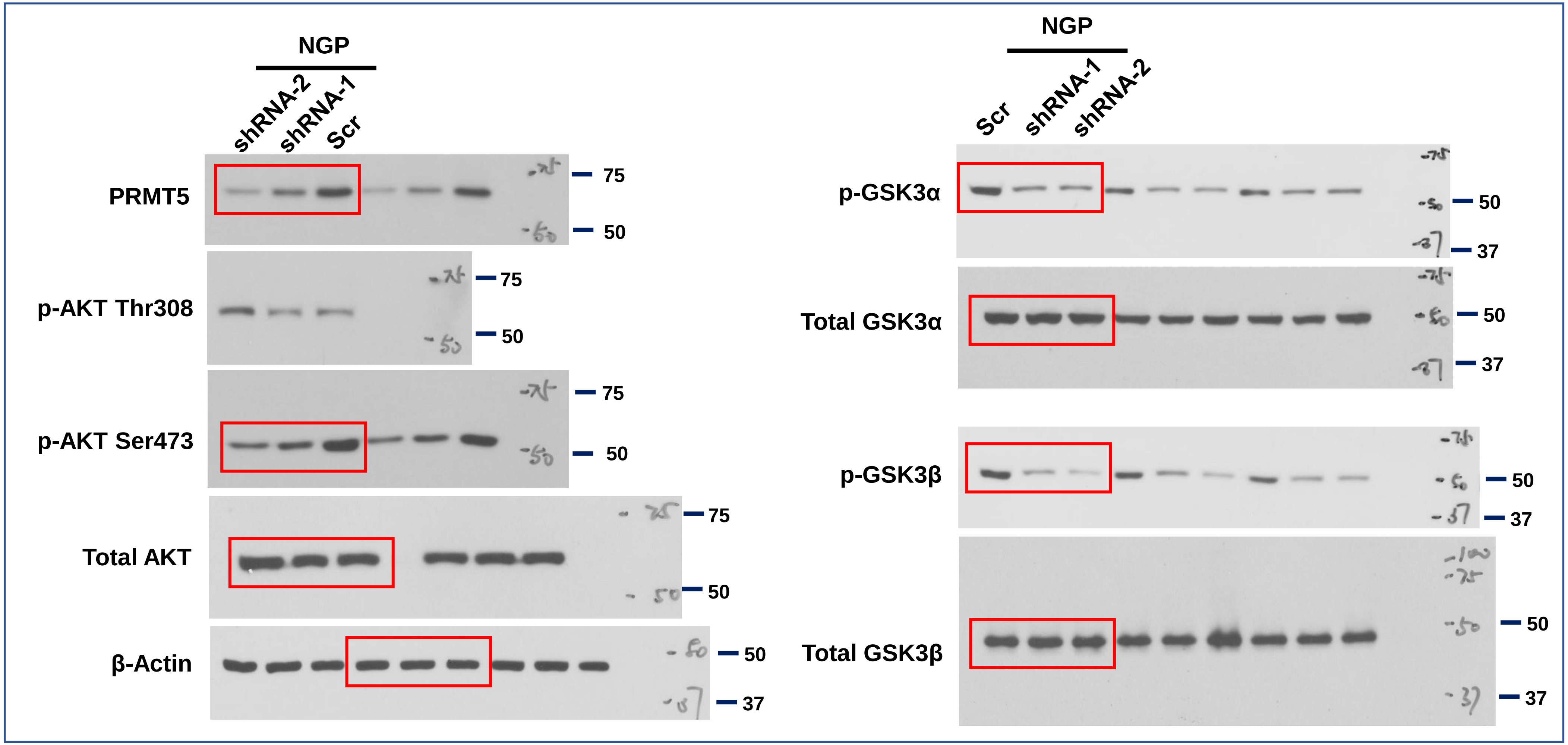

Fig. 3c right panel

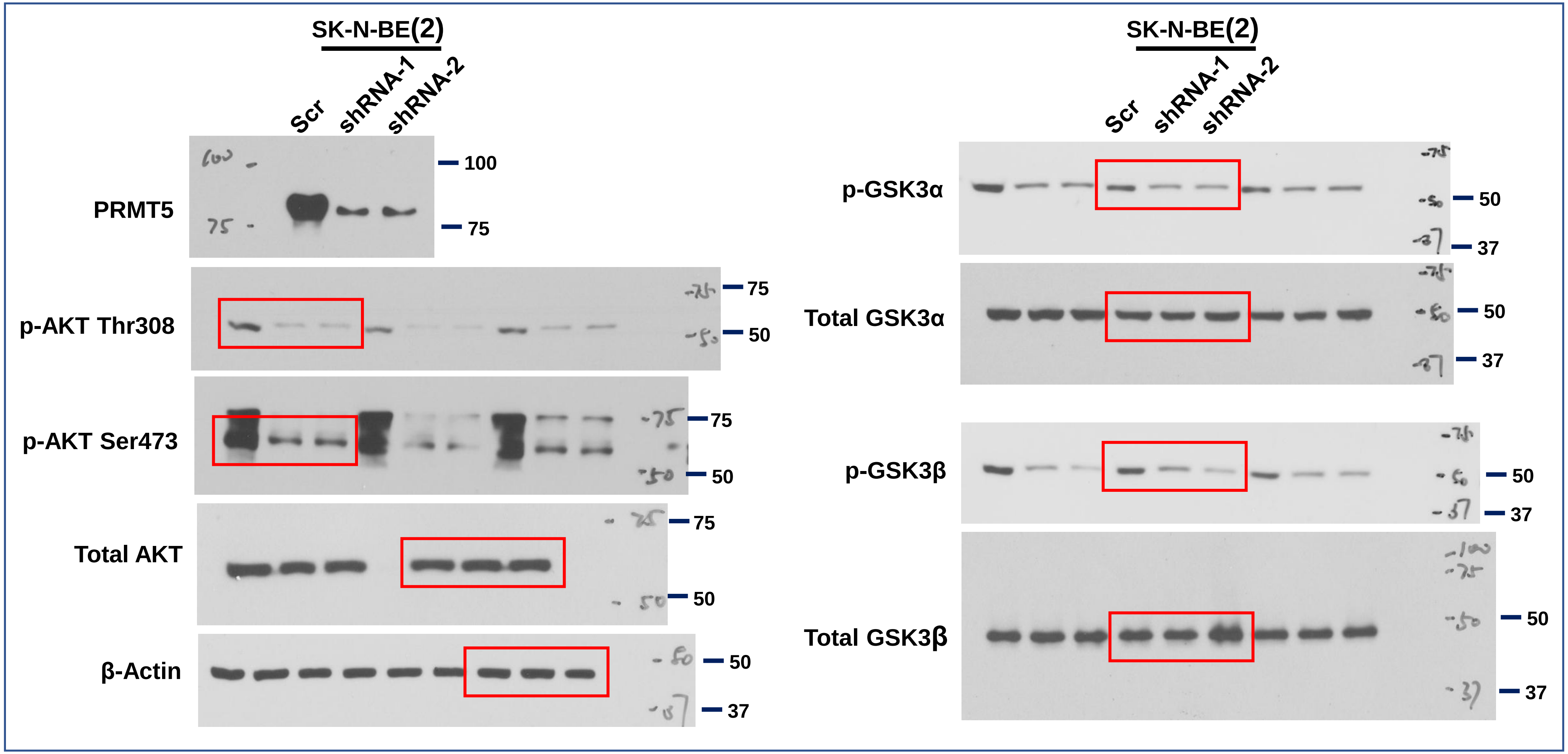

Fig. 3d

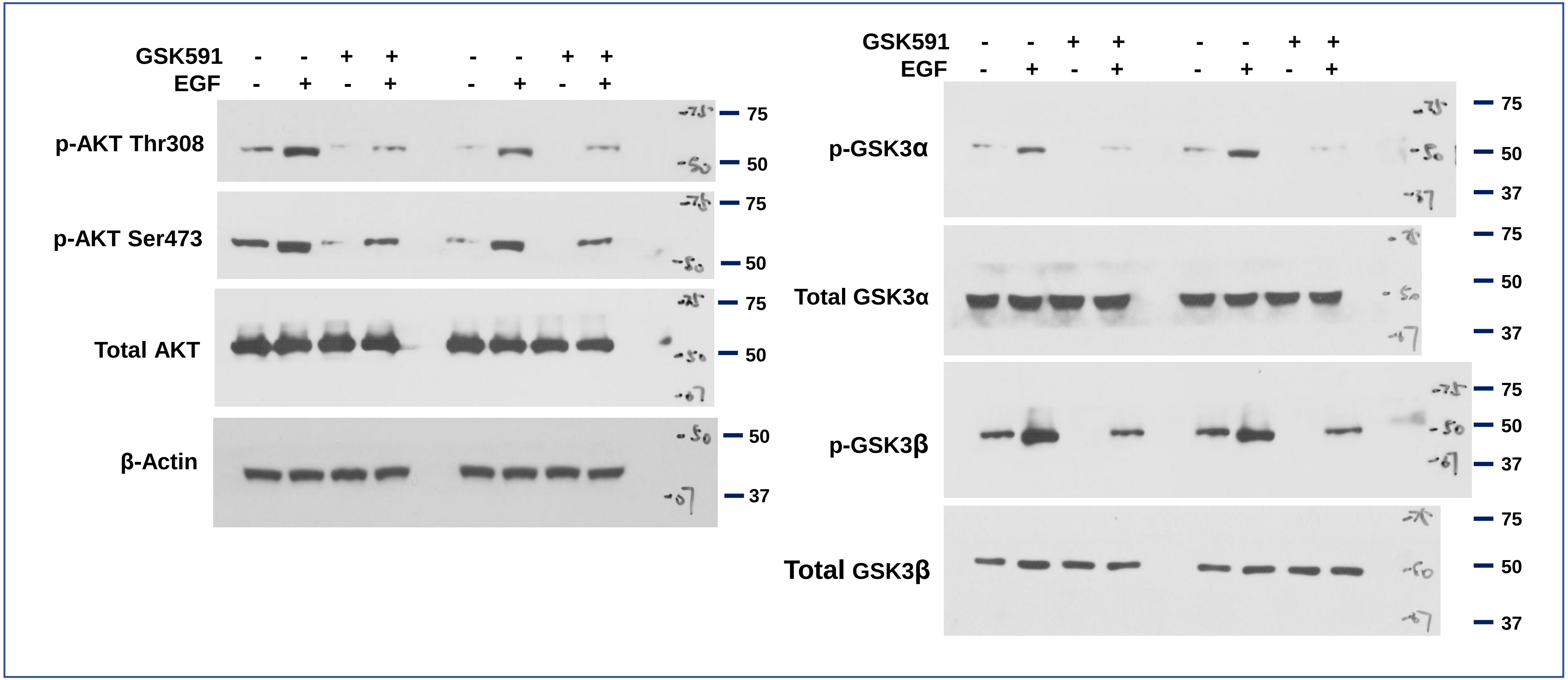

GSK591 - - + + - - + +
EGF - + - + - + - +
p-AKT Thr308
p-AKT Ser473
Total AKT
β-Actin
75
50
37
p-GSK3α
Total GSK3α
p-GSK3β
Total GSK3β

Fig. 3e left panel

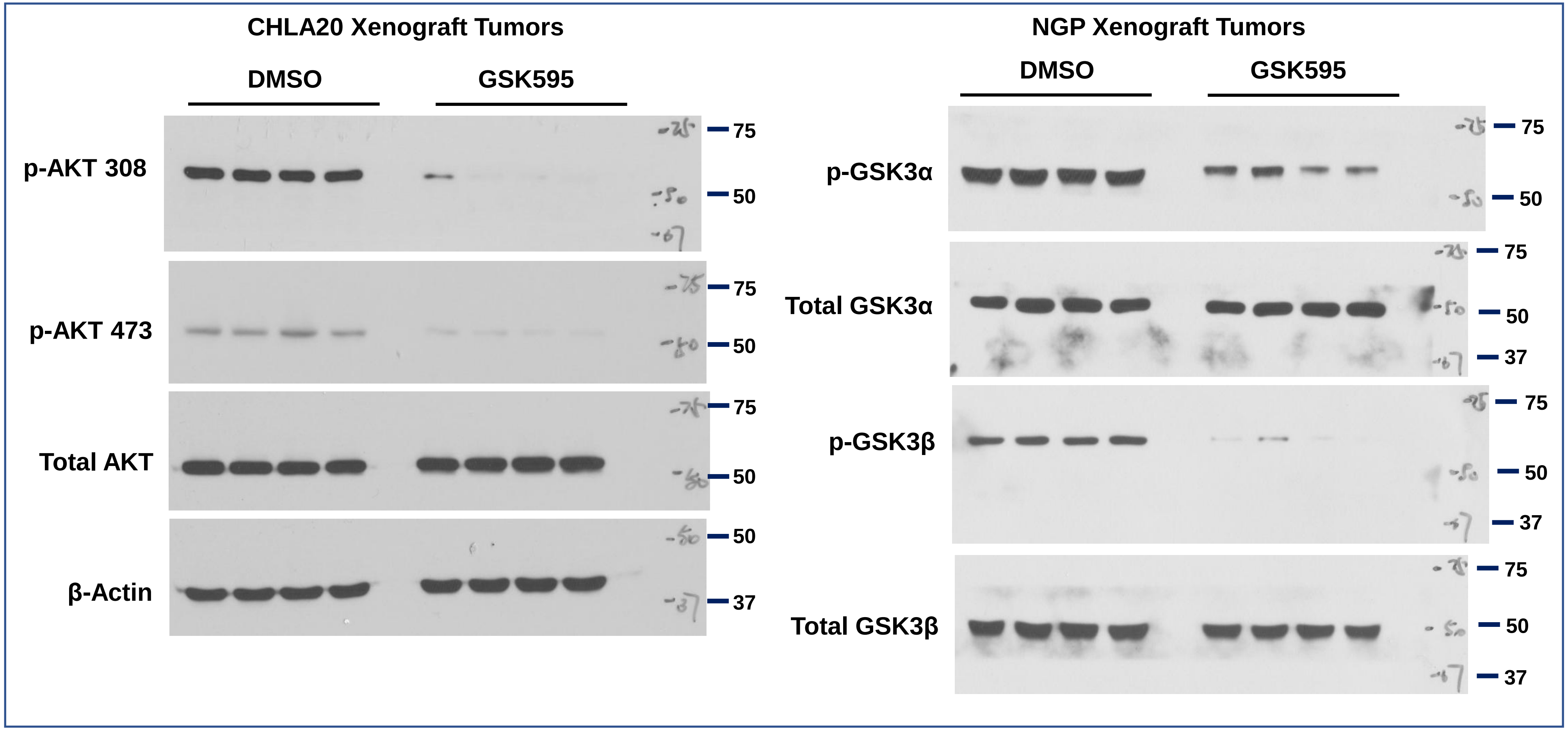

Fig. 3e right panel

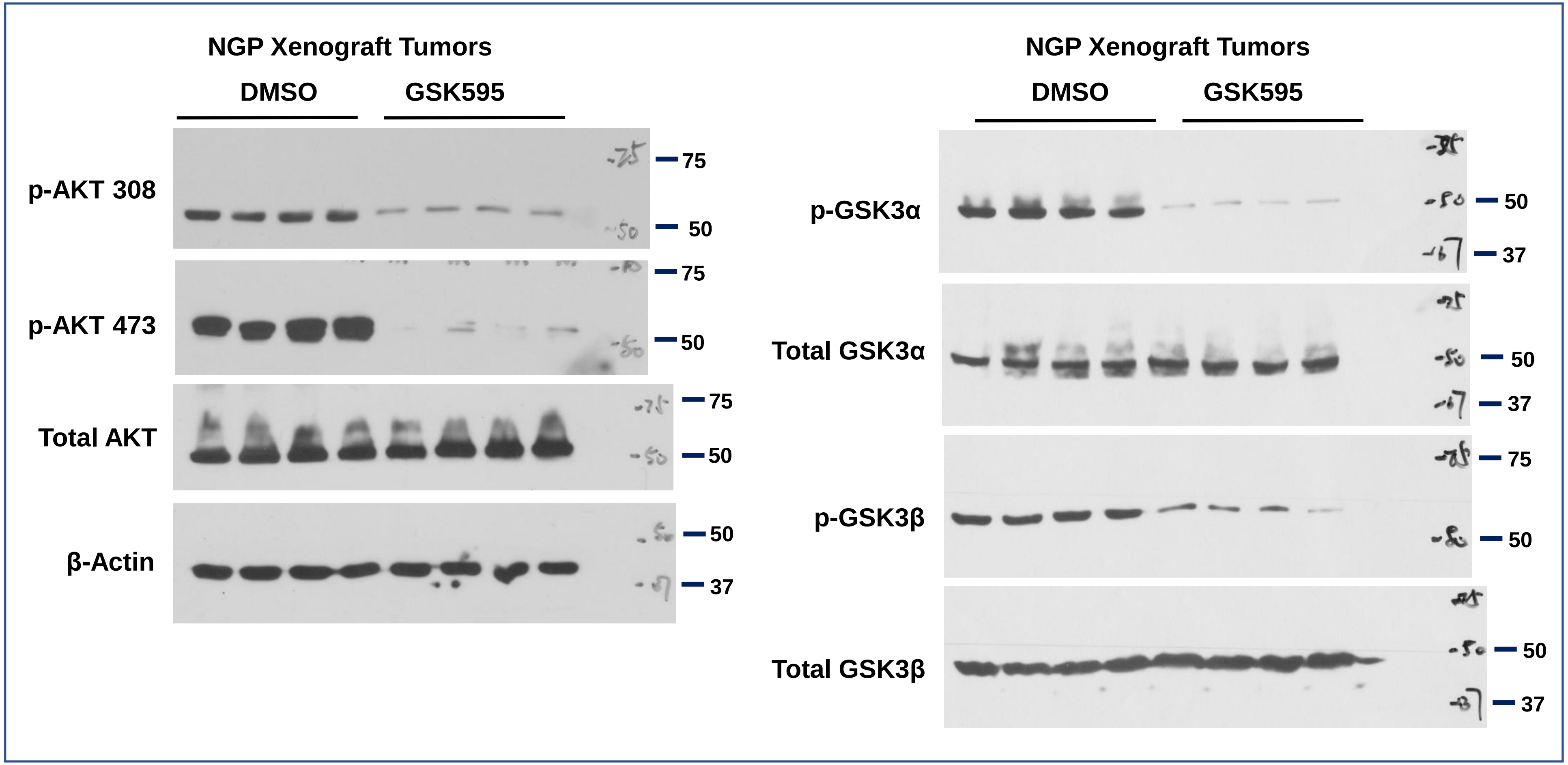

Fig. 4a

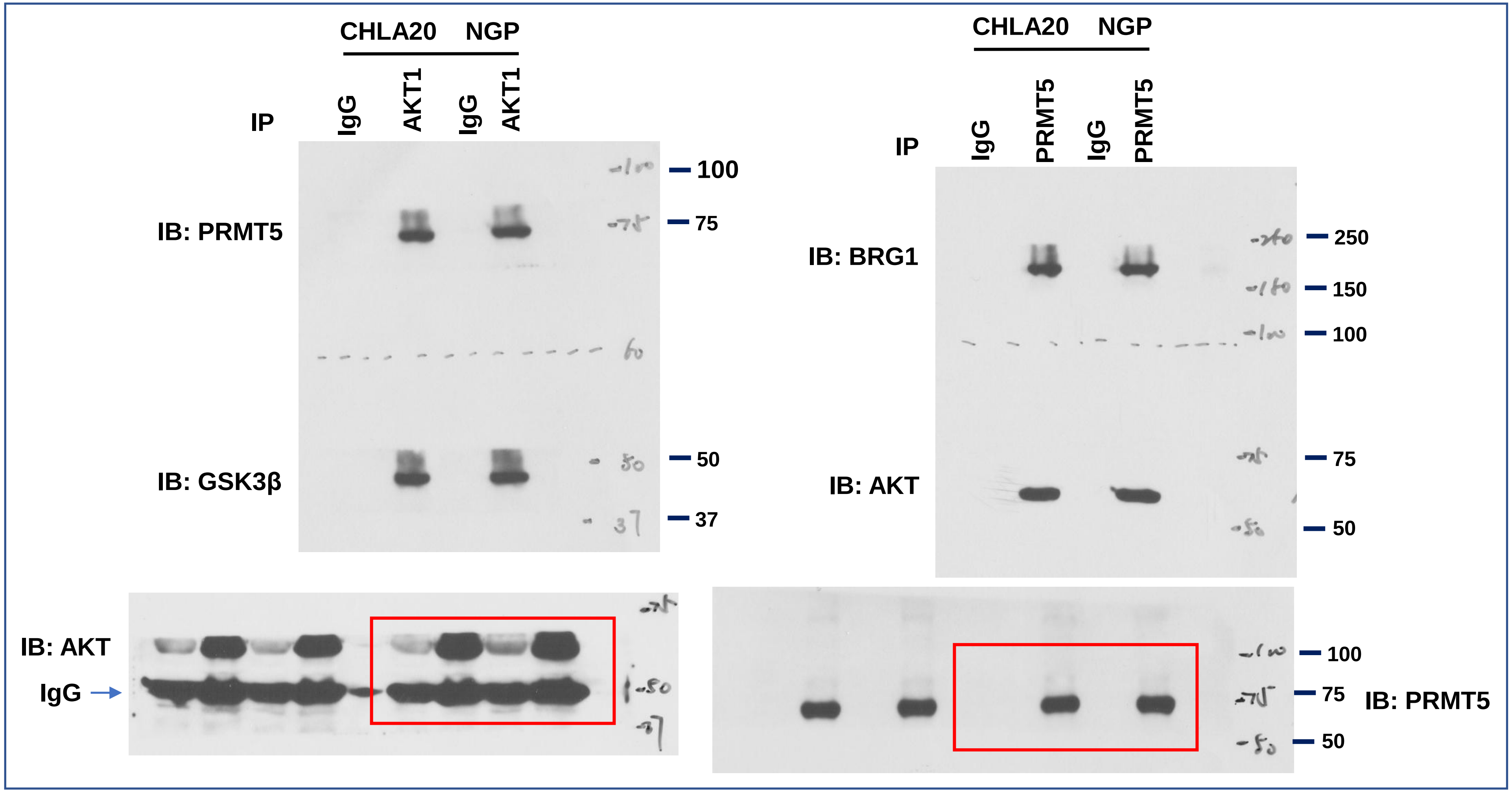
CHLA20 NGP
IP
IgG
AKT1
IgG
AKT1
IB: PRMT5
IB: GSK3β
100
75
50
37
CHLA20 NGP
IP
IgG
PRMT5
IgG
PRMT5
IB: BRG1
IB: AKT
250
150
100
75
50
IB: AKT
IgG
IB: PRMT5
100
75
50

Fig. 4a continued

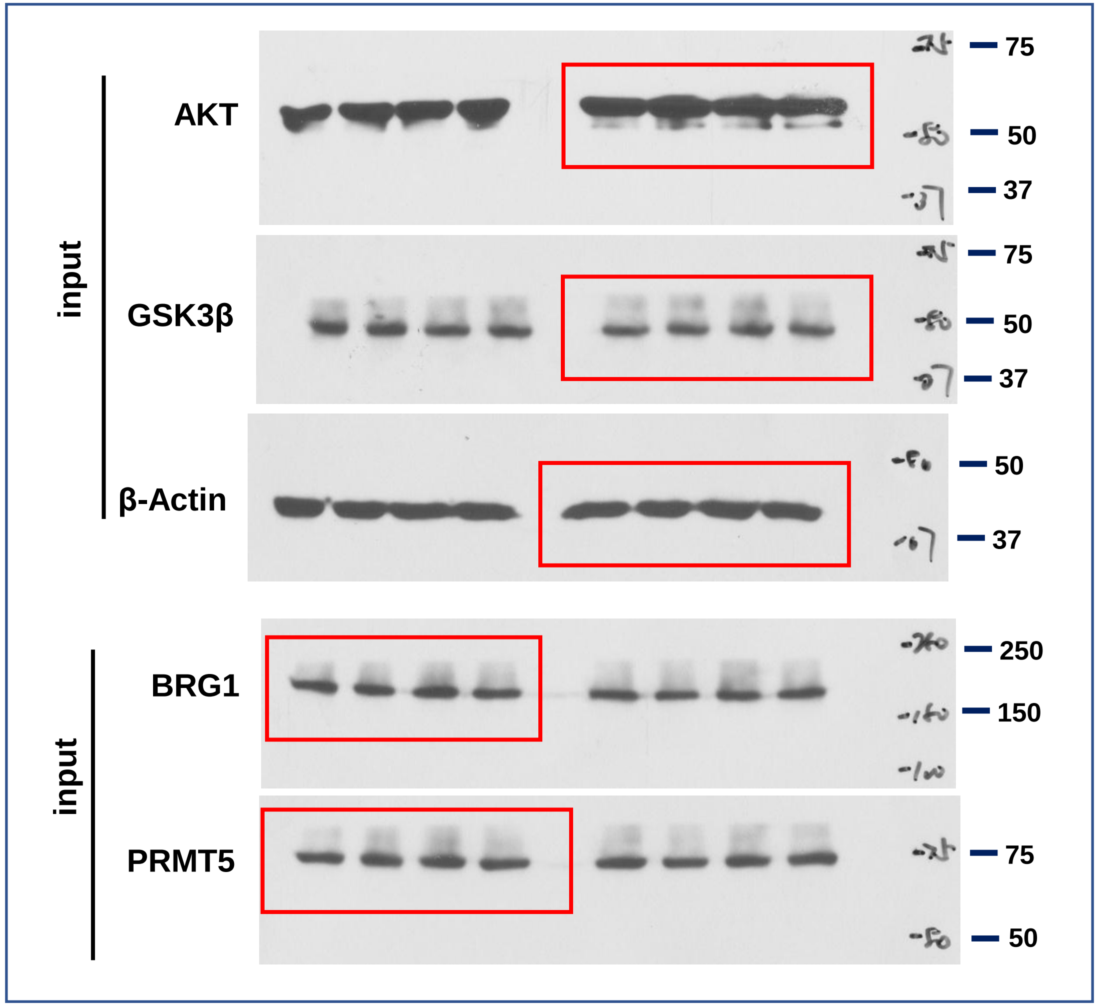

Fig. 4b

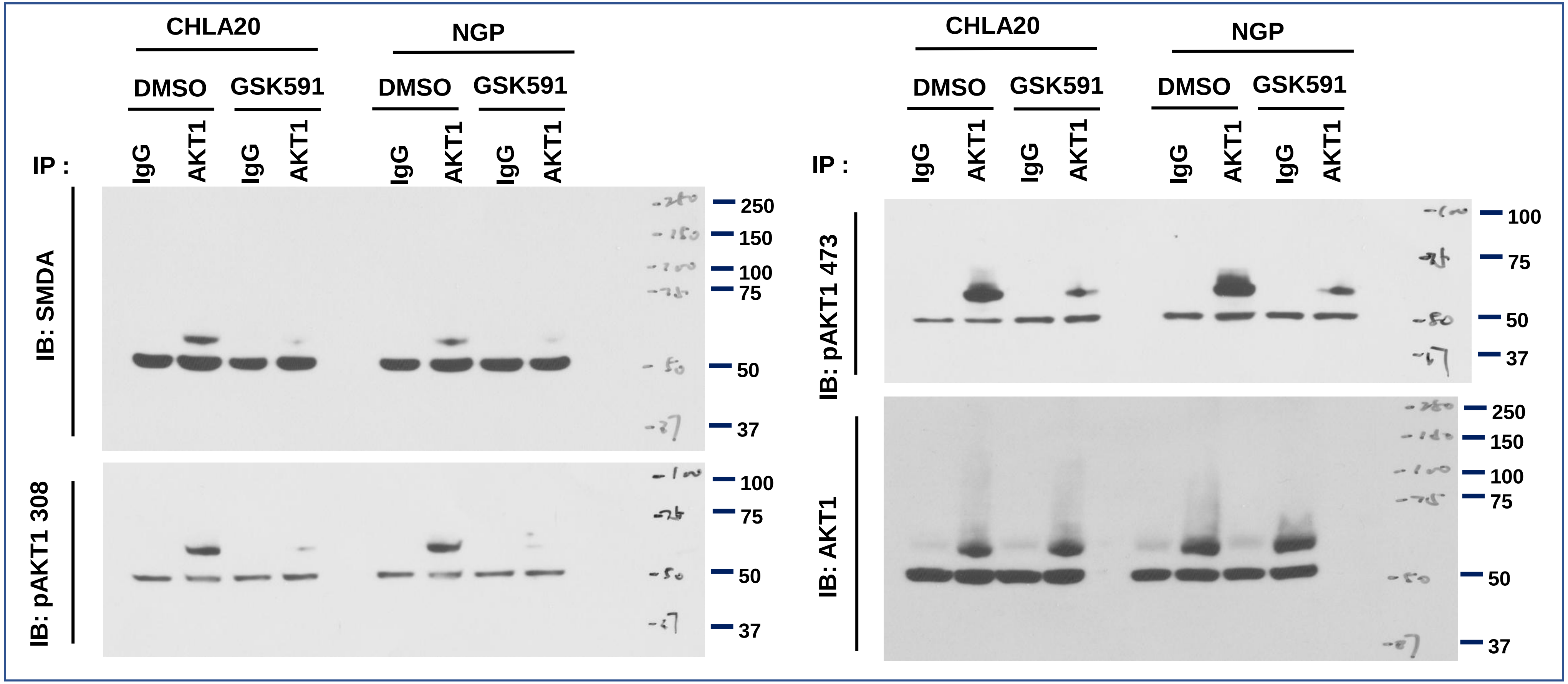

CHLA20
NGP
DMSO
GSK591
DMSO
GSK591
IP :
IgG
AKT1
IgG
AKT1
IgG
AKT1
IgG
AKT1
IB: SMDA
250
150
100
75
50
37
IB: pAKT1 308
100
75
50
37
CHLA20
NGP
DMSO
GSK591
DMSO
GSK591
IP :
IgG
AKT1
IgG
AKT1
IgG
AKT1
IgG
AKT1
IB: pAKT1 473
100
75
50
37
IB: AKT1
250
150
100
75
50
37

Fig. 4c

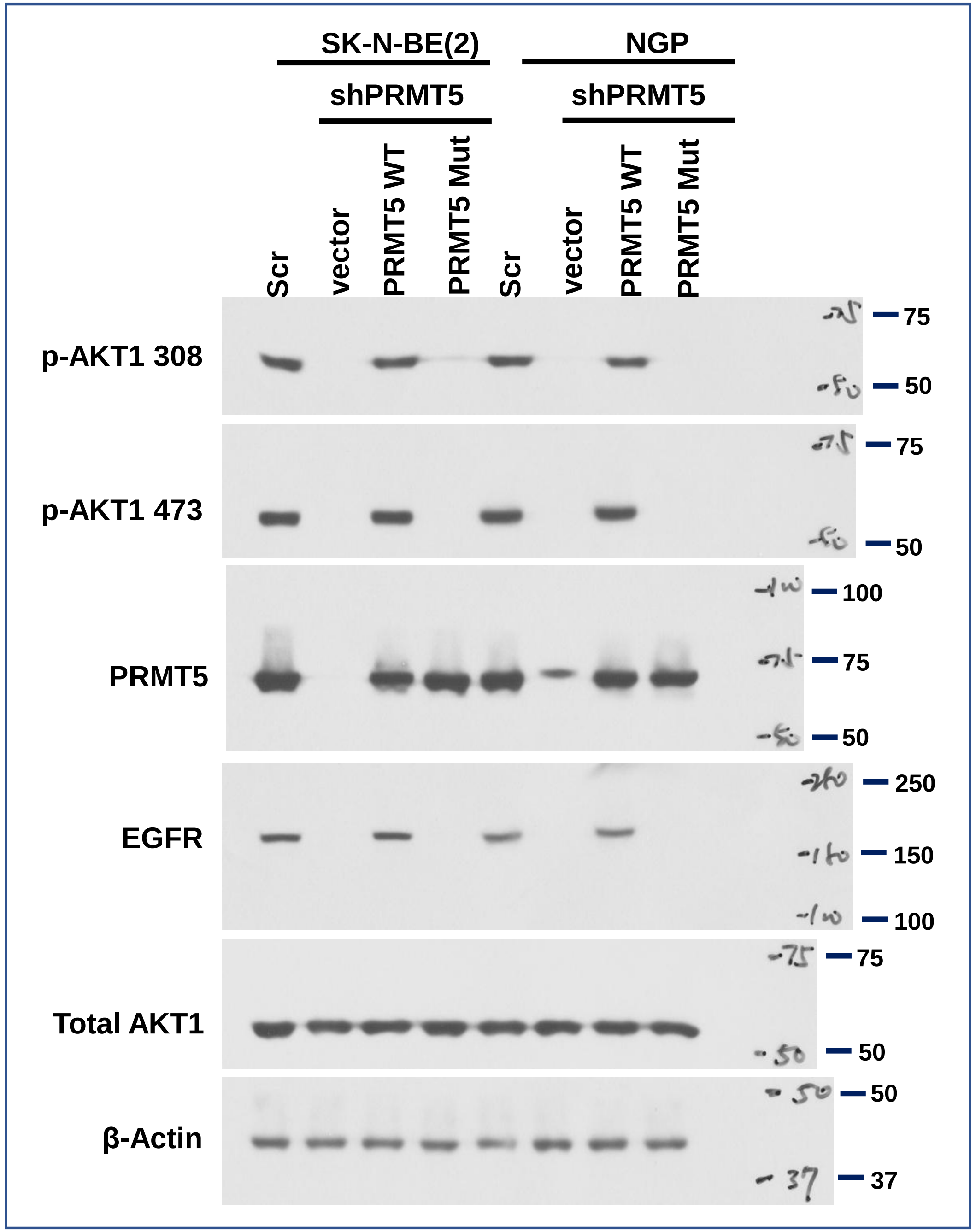

SK-N-BE(2)
NGP
shPRMT5
shPRMT5
Scr
vector
PRMT5 WT
PRMT5 Mut
Scr
vector
PRMT5 WT
PRMT5 Mut
p-AKT1 308
p-AKT1 473
PRMT5
EGFR
Total AKT1
β-Actin
75
50
75
50
100
75
50
250
150
100
75
50
50
37

Fig. 4d

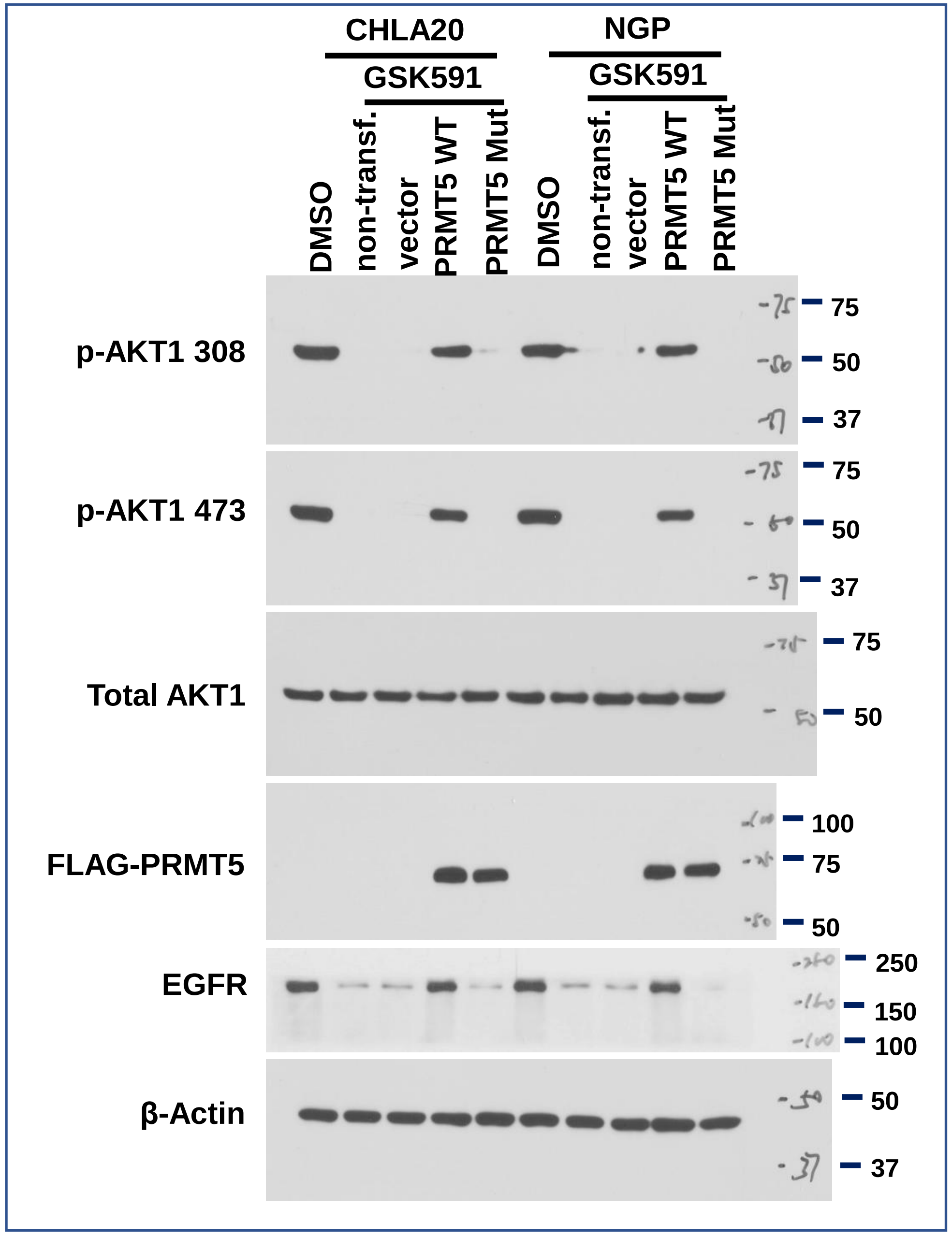

CHLA20
NGP
GSK591
GSK591
DMSO
non-transf.
vector
PRMT5 WT
PRMT5 Mut
DMSO
non-transf.
vector
PRMT5 WT
PRMT5 Mut
p-AKT1 308
p-AKT1 473
Total AKT1
FLAG-PRMT5
EGFR
β-Actin
75
50
37
75
50
37
75
50
100
75
50
250
150
100
50
37

Fig. 4e

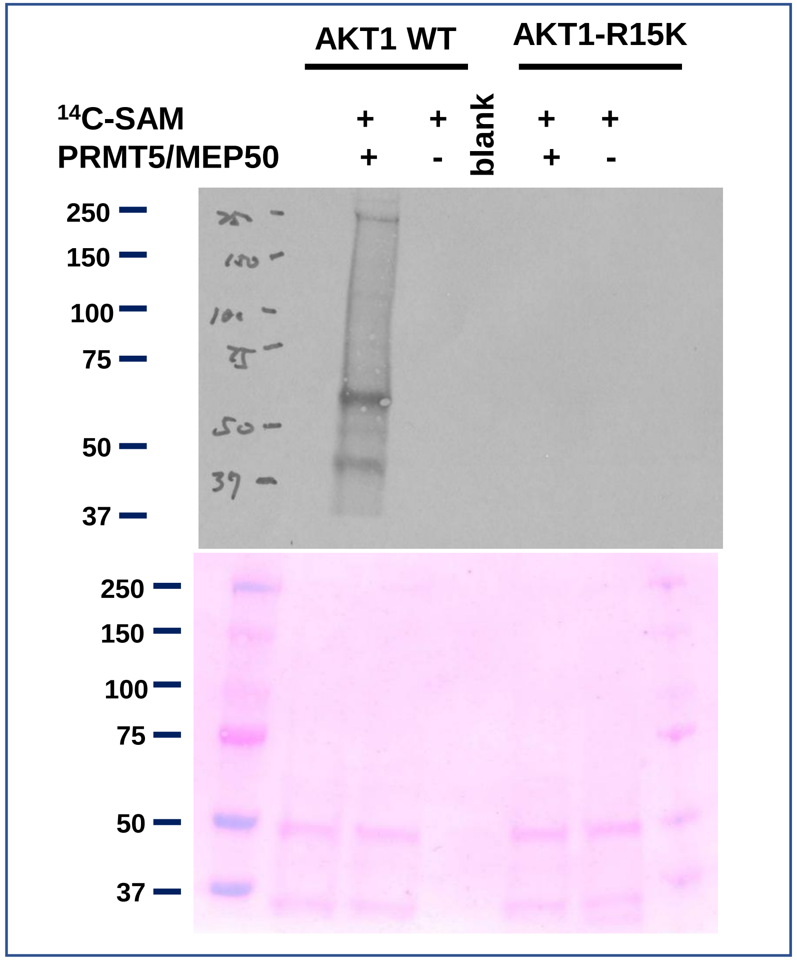
AKT1 WT
AKT1-R15K
^{14}C-SAM + + + +
PRMT5/MEP50 + - + -
blank
250
150
100
75
50
37
250
150
100
75
50
37

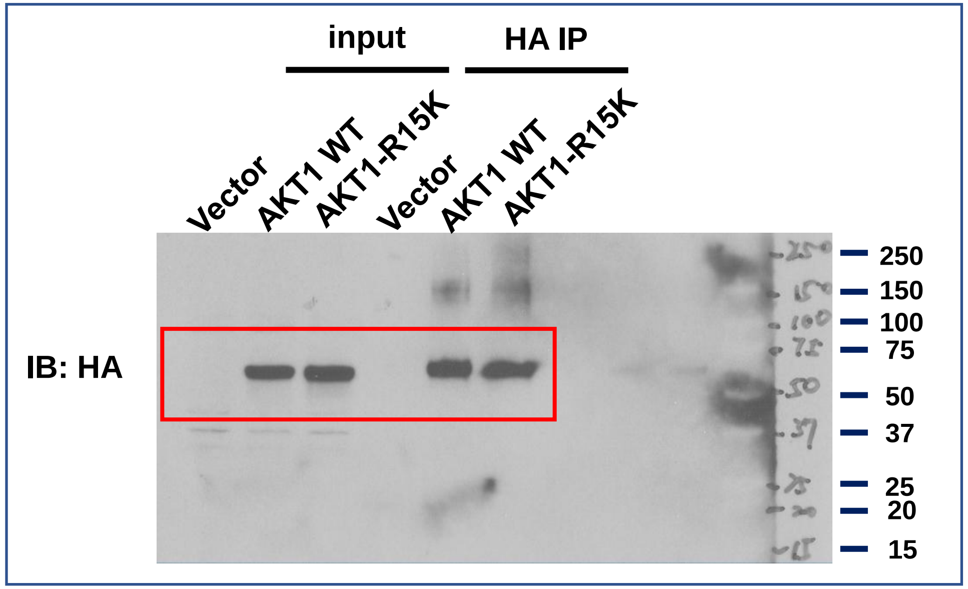
input
HA IP
Vector
AKT1 WT
AKT1-R15K
Vector
AKT1 WT
AKT1-R15K
IB: HA
250
150
100
75
50
37
25
20
15

Fig. 4f

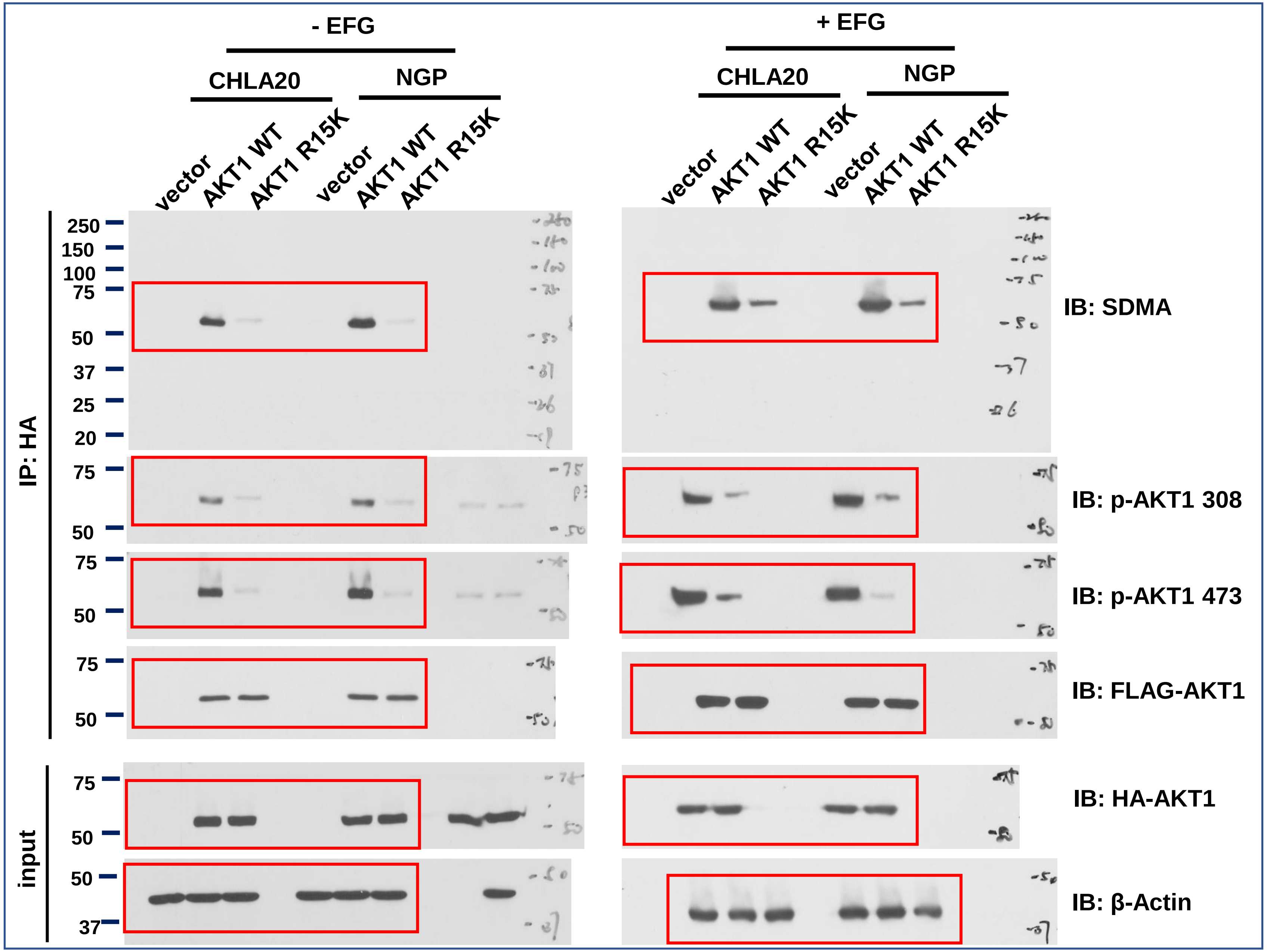

- EFG
+ EFG
CHLA20
NGP
vector
AKT1 WT
AKT1 R15K
IP: HA
input
250
150
100
75
50
37
25
20
IB: SDMA
IB: p-AKT1 308
IB: p-AKT1 473
IB: FLAG-AKT1
IB: HA-AKT1
IB: β-Actin

Fig. 5a

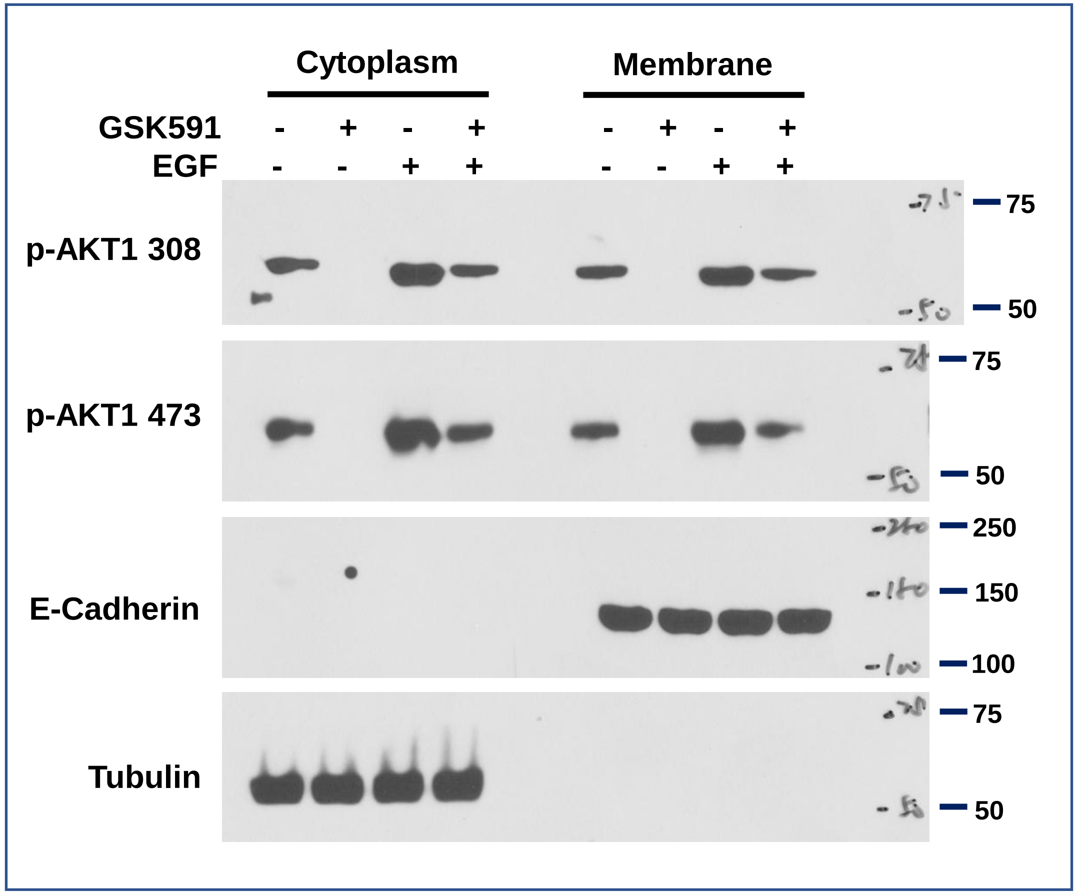
Cytoplasm
Membrane
GSK591 - + - + - + - +
EGF - - + + - - + +
p-AKT1 308
p-AKT1 473
E-Cadherin
Tubulin
75
50
75
50
250
150
100
75
50

Fig. 5b

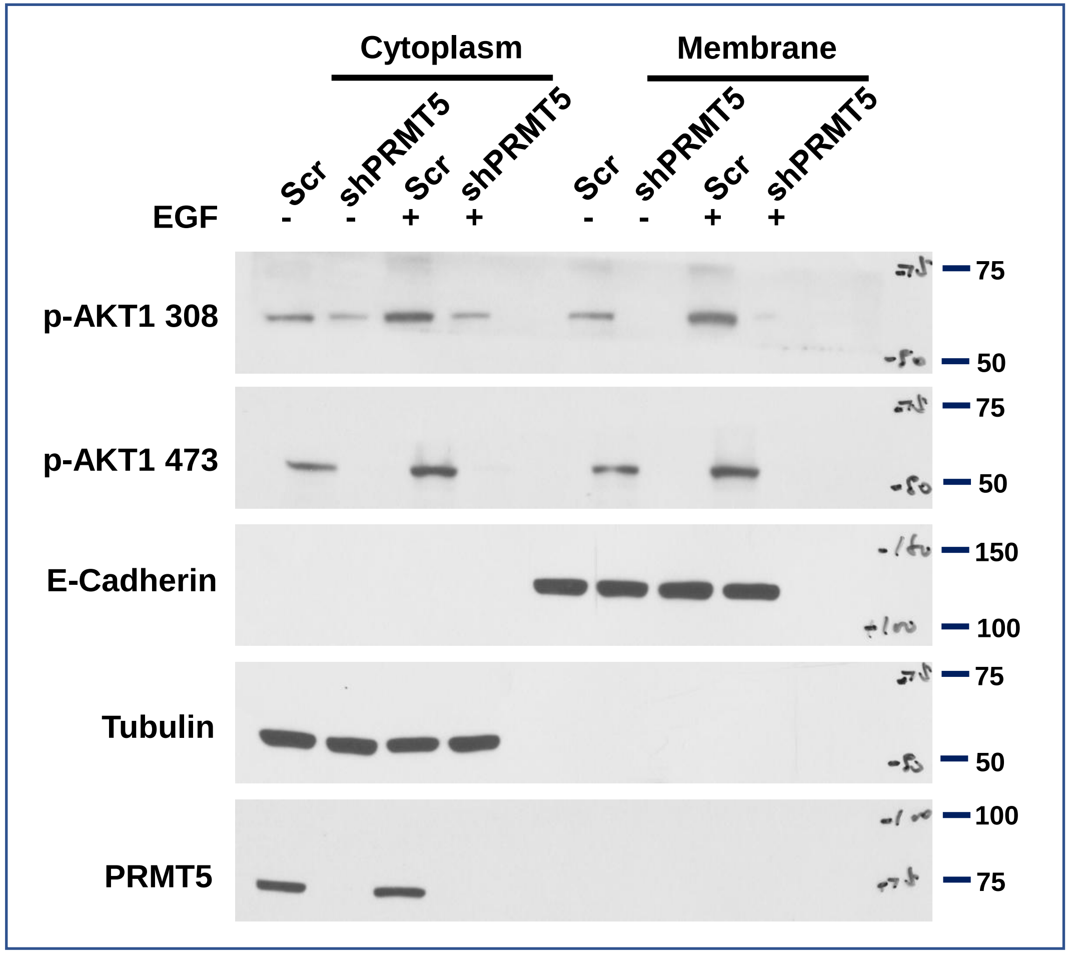
Cytoplasm
Membrane
Scr
shPRMT5
Scr
shPRMT5
Scr
shPRMT5
Scr
shPRMT5
EGF - - + + - - + +
p-AKT1 308
p-AKT1 473
E-Cadherin
Tubulin
PRMT5
75
50
75
50
150
100
75
50
100
75

Fig. 5d

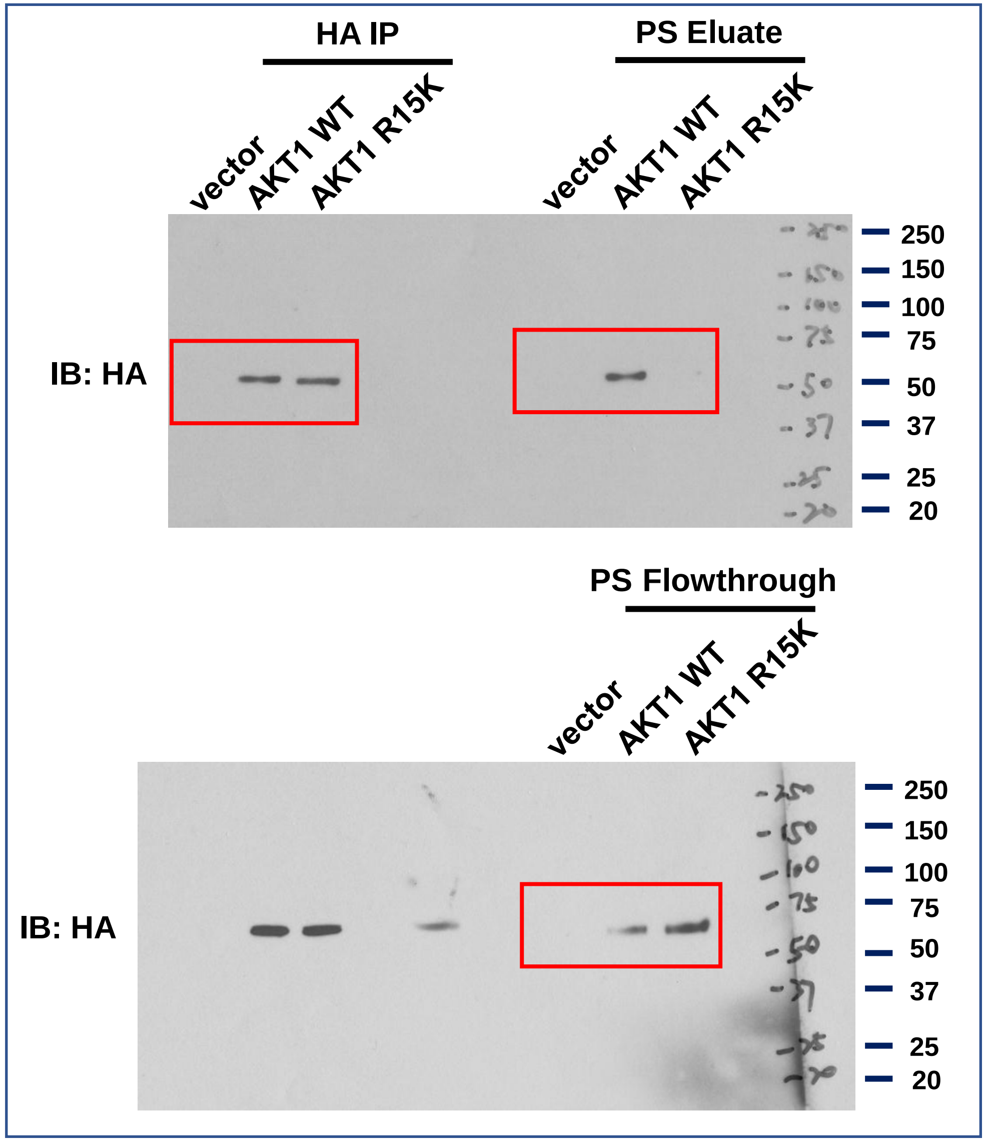
HA IP
vector
AKT1 WT
AKT1 R15K
PS Eluate
vector
AKT1 WT
AKT1 R15K
IB: HA
250
150
100
75
50
37
25
20
PS Flowthrough
vector
AKT1 WT
AKT1 R15K
IB: HA
250
150
100
75
50
37
25
20

Fig. 5e

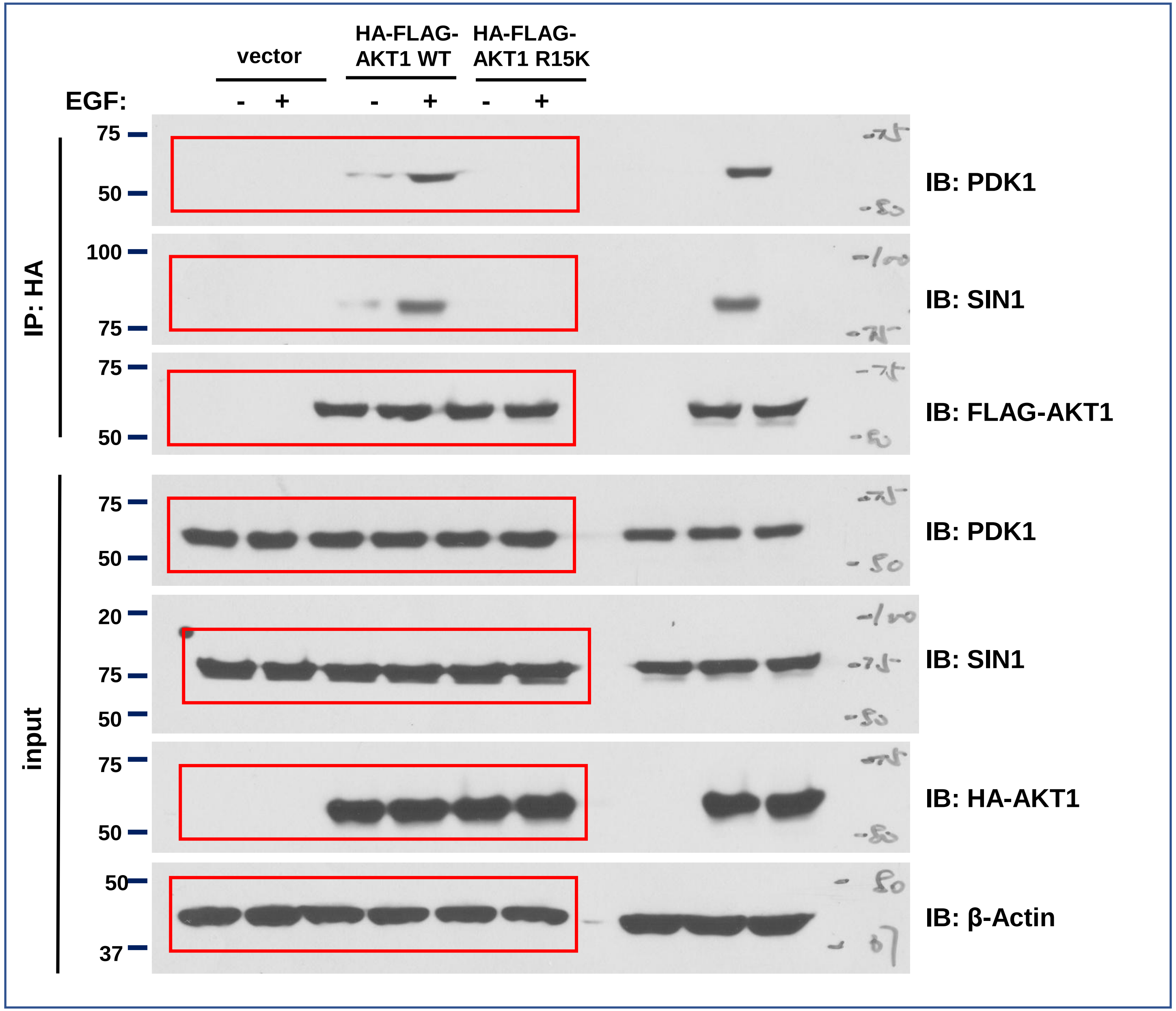

vector
HA-FLAG-AKT1 WT
HA-FLAG-AKT1 R15K
EGF:
- + - + - +
IP: HA
input
75
50
100
75
75
50
75
50
20
75
50
75
50
50
37
IB: PDK1
IB: SIN1
IB: FLAG-AKT1
IB: PDK1
IB: SIN1
IB: HA-AKT1
IB: β-Actin

Fig. 5f

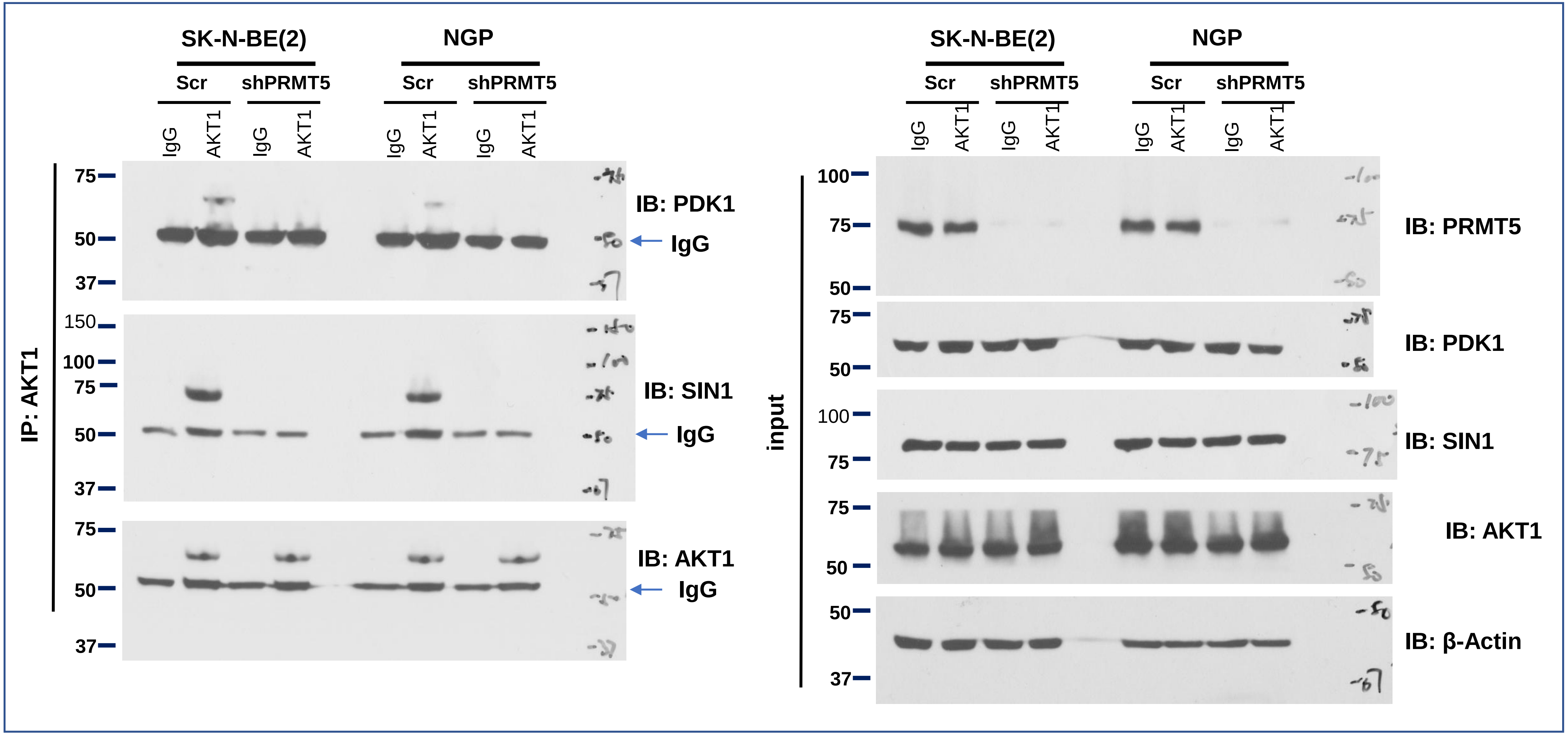

SK-N-BE(2)
NGP
Scr
shPRMT5
IgG
AKT1
IP: AKT1
IB: PDK1
IgG
IB: SIN1
IB: AKT1
input
IB: PRMT5
IB: β-Actin
150
100
75
50
37

Fig. 5g

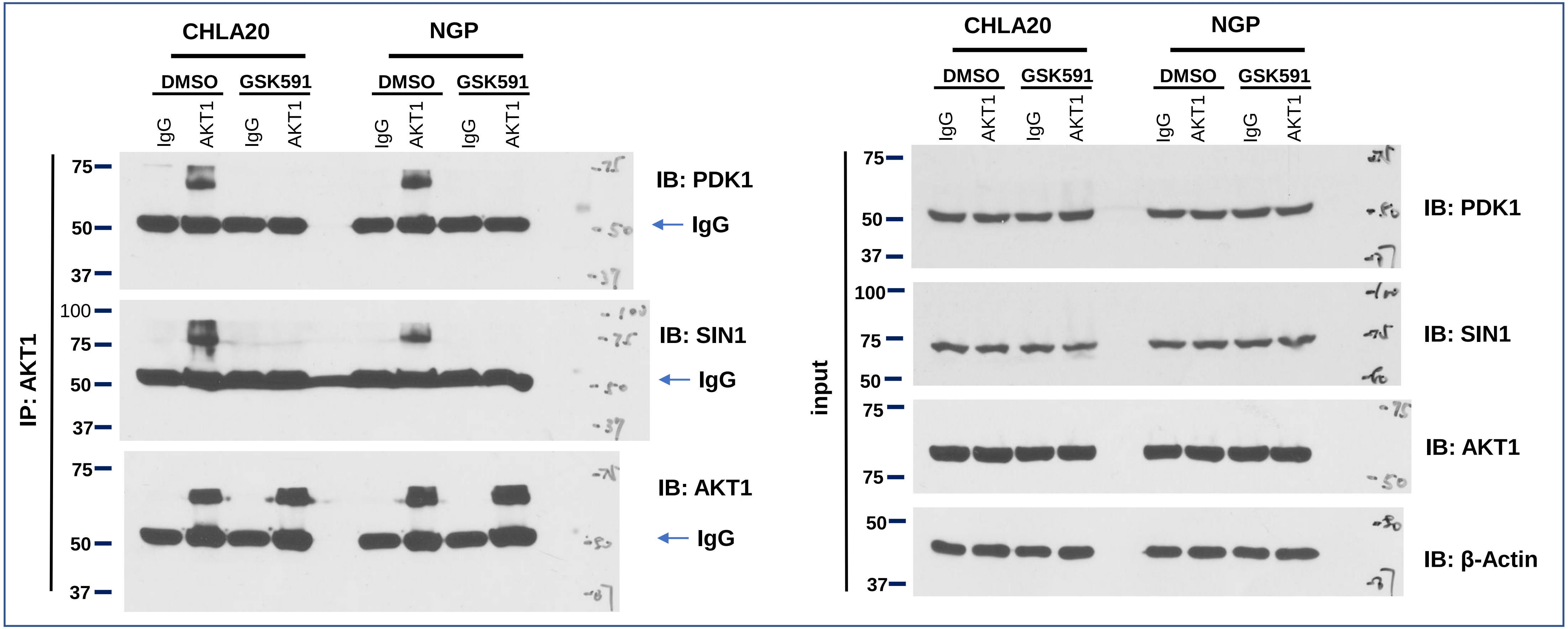
CHLA20
NGP
DMSO
GSK591
IgG
AKT1
IP: AKT1
IB: PDK1
IgG
IB: SIN1
IB: AKT1
input
IB: β-Actin
75
50
37
100

Fig. 6a

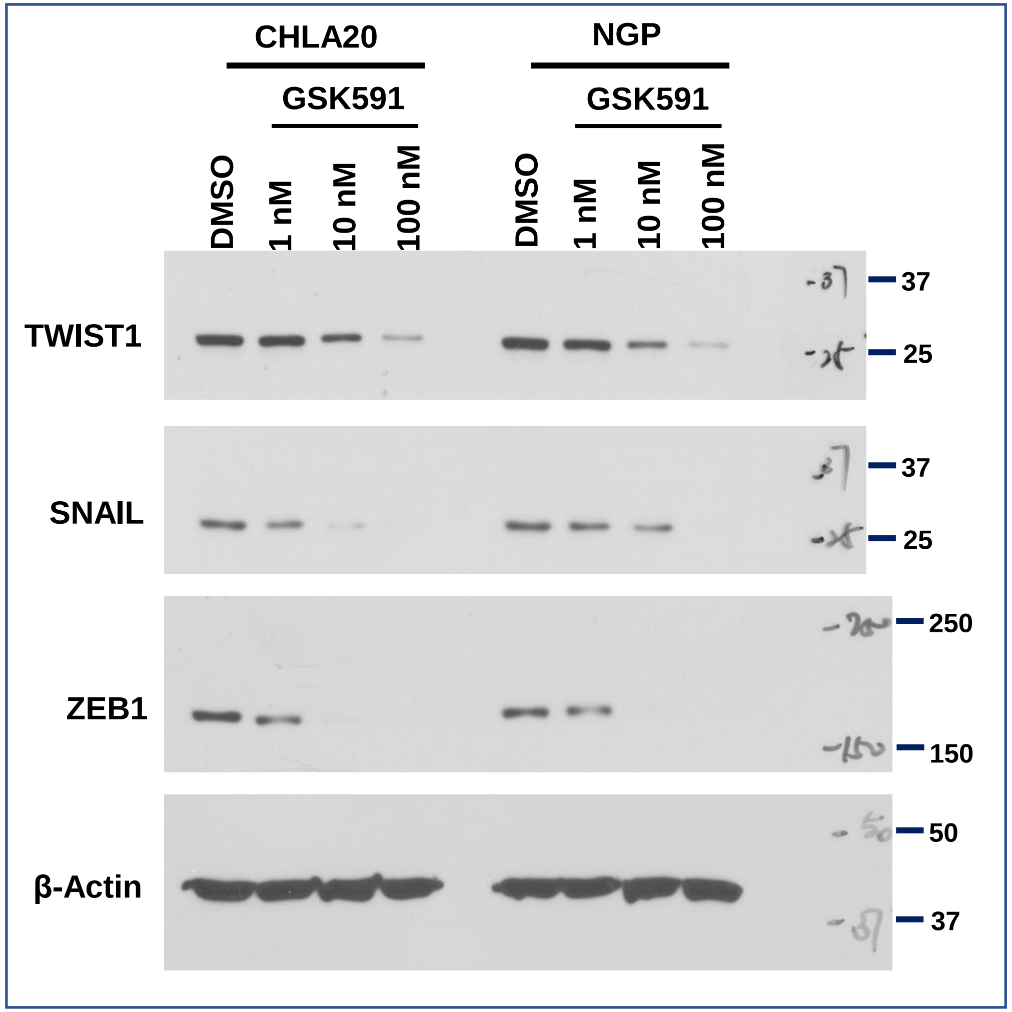

Fig. 6b_Left

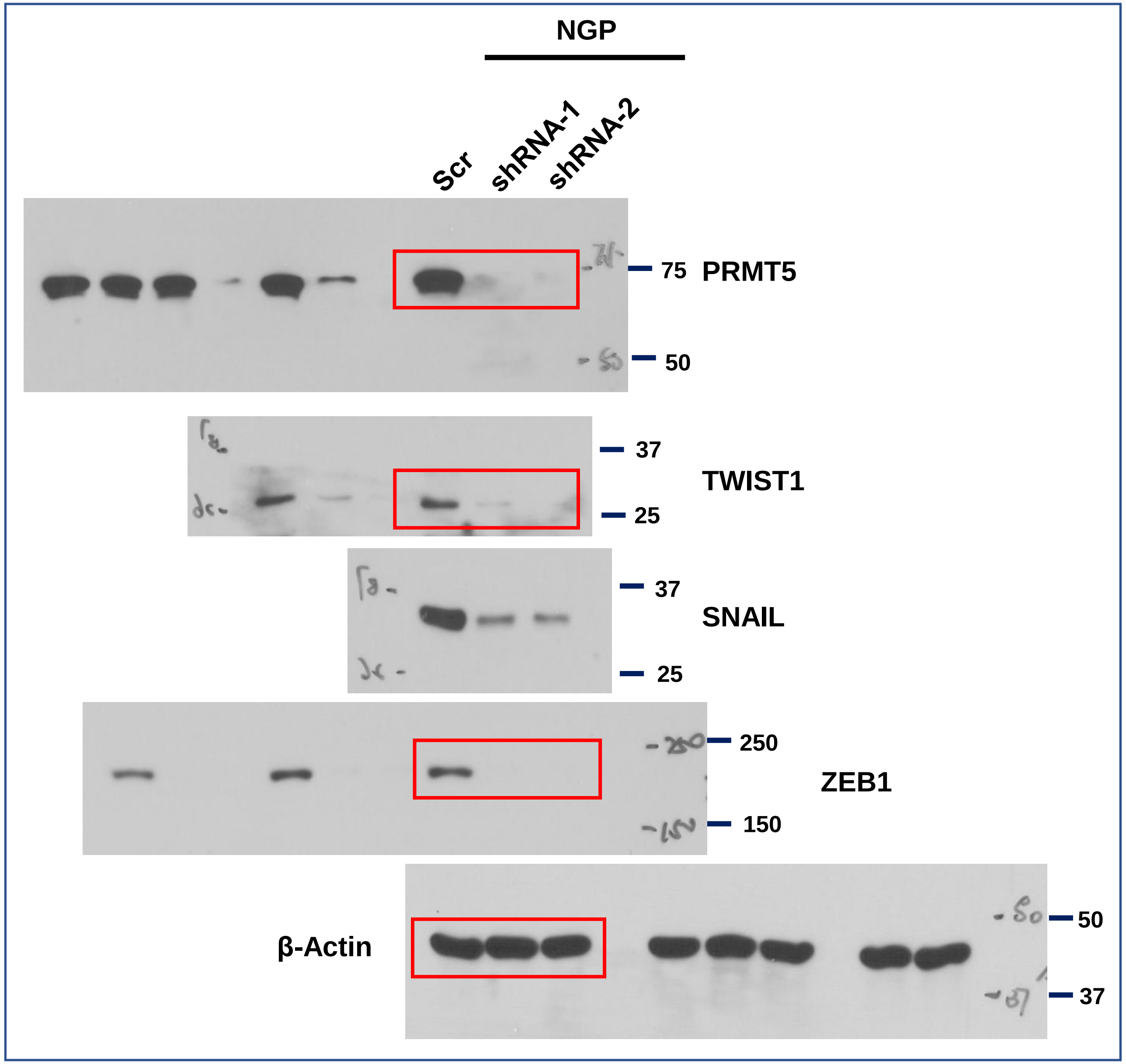

Fig. 6b_Right

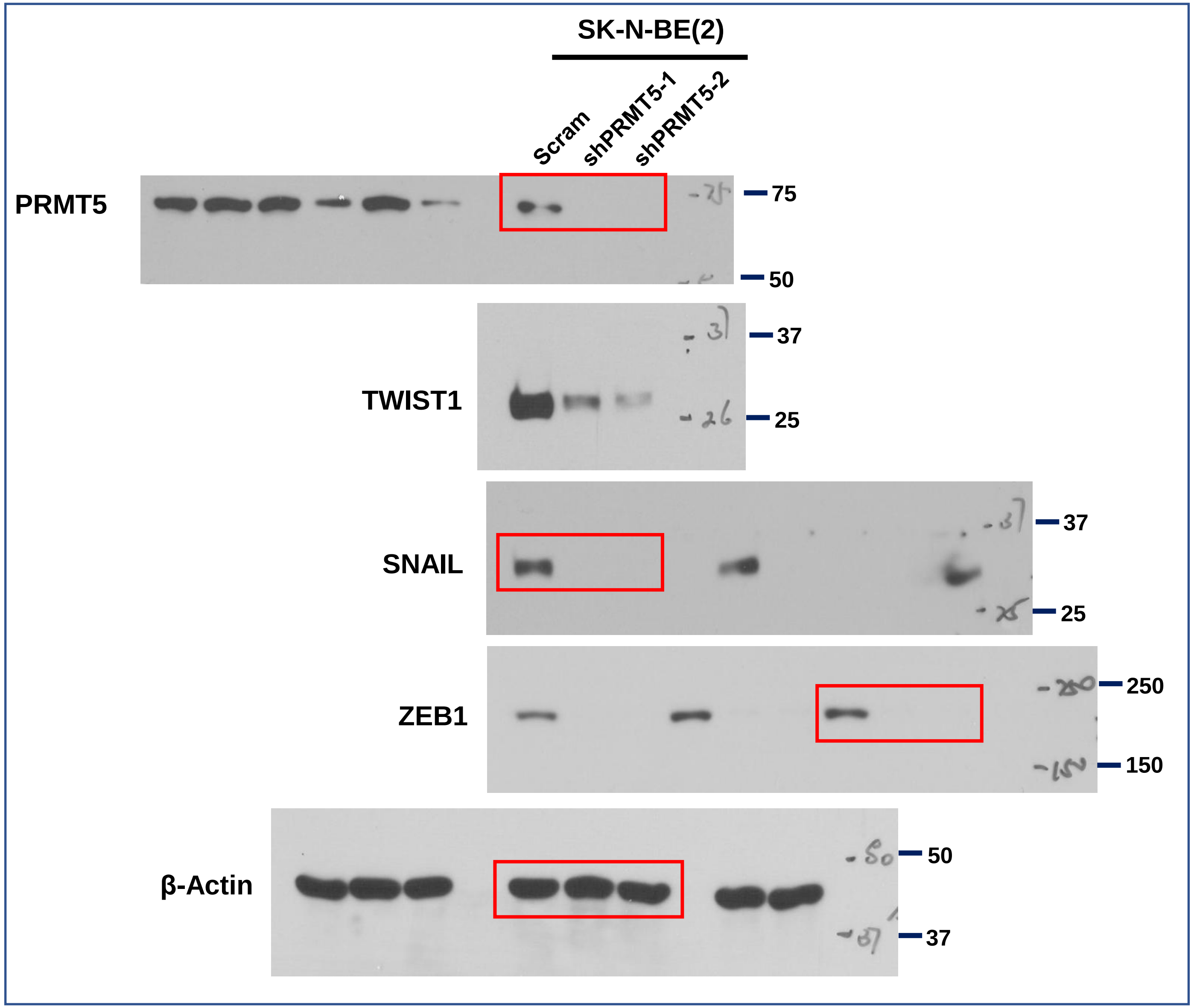
SK-N-BE(2)
Scram
shPRMT5-1
shPRMT5-2
PRMT5
75
50
TWIST1
37
25
SNAIL
37
25
ZEB1
250
150
β-Actin
50
37

Fig. 6c

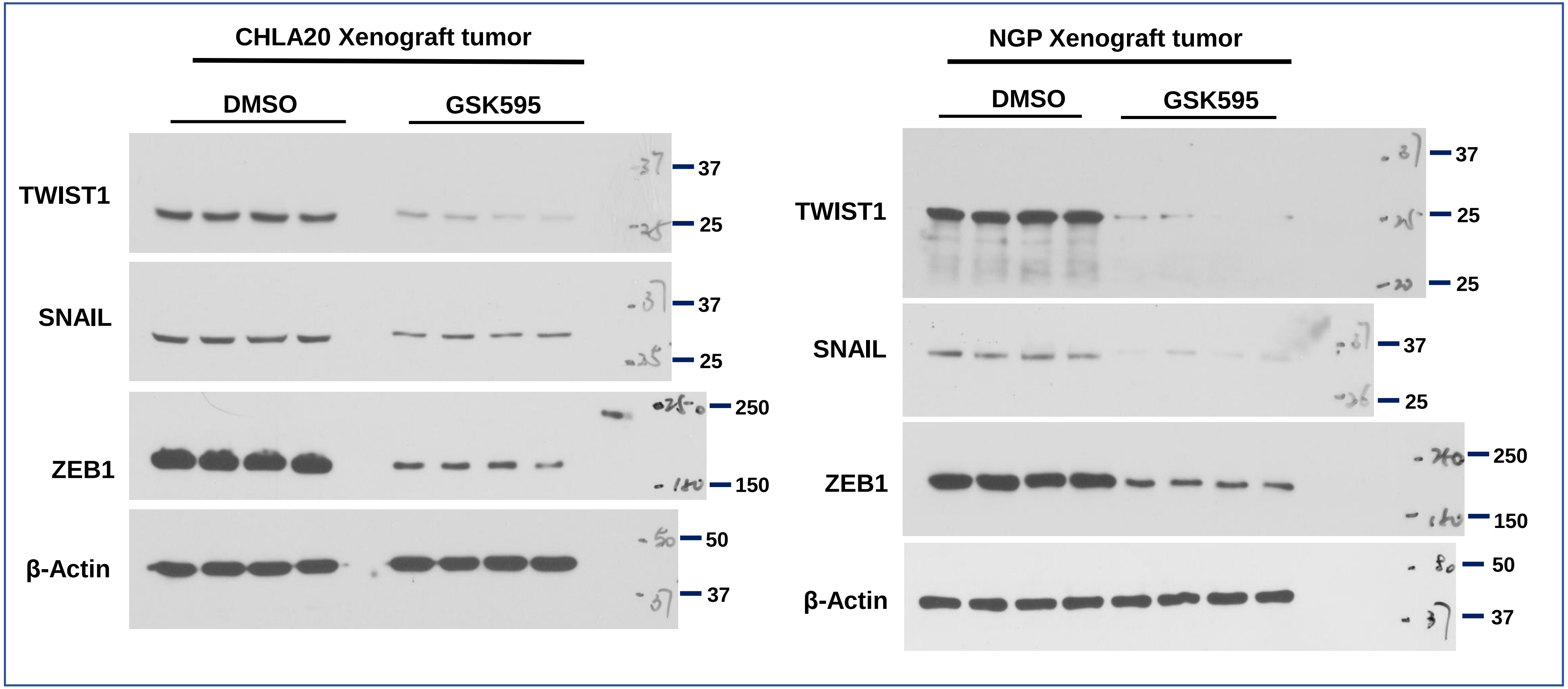

CHLA20 Xenograft tumor
DMSO
GSK595
TWIST1
SNAIL
ZEB1
β-Actin
37
25
37
25
250
150
50
37
NGP Xenograft tumor
DMSO
GSK595
TWIST1
SNAIL
ZEB1
β-Actin
37
25
25
37
25
250
150
50
37

Fig. 6h

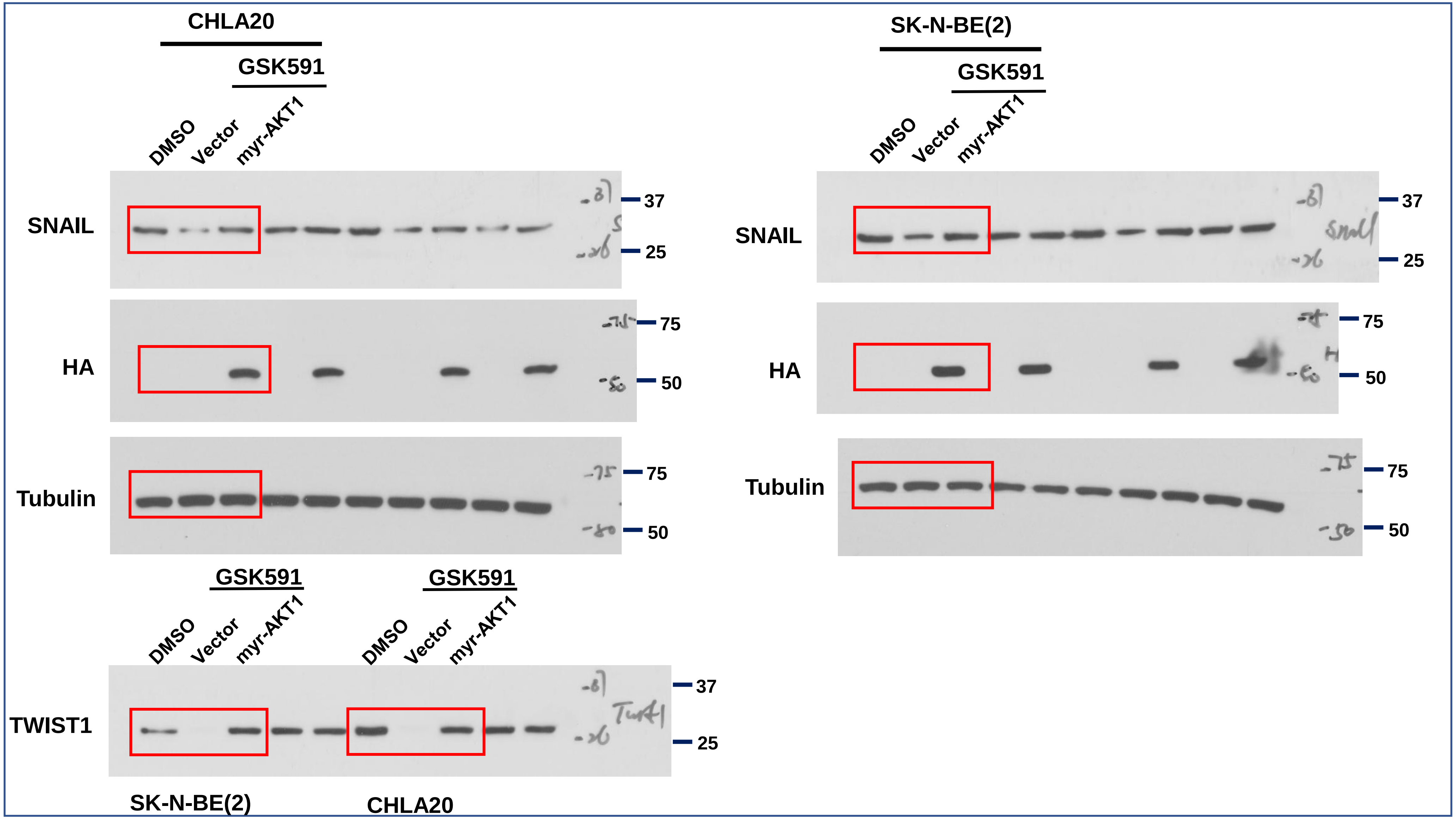

CHLA20
GSK591
DMSO
Vector
myr-AKT1
SNAIL
HA
Tubulin
37
25
75
50
SK-N-BE(2)
GSK591
DMSO
Vector
myr-AKT1
SNAIL
HA
Tubulin
GSK591
GSK591
DMSO
Vector
myr-AKT1
DMSO
Vector
myr-AKT1
TWIST1
SK-N-BE(2)
CHLA20

Fig. 6i

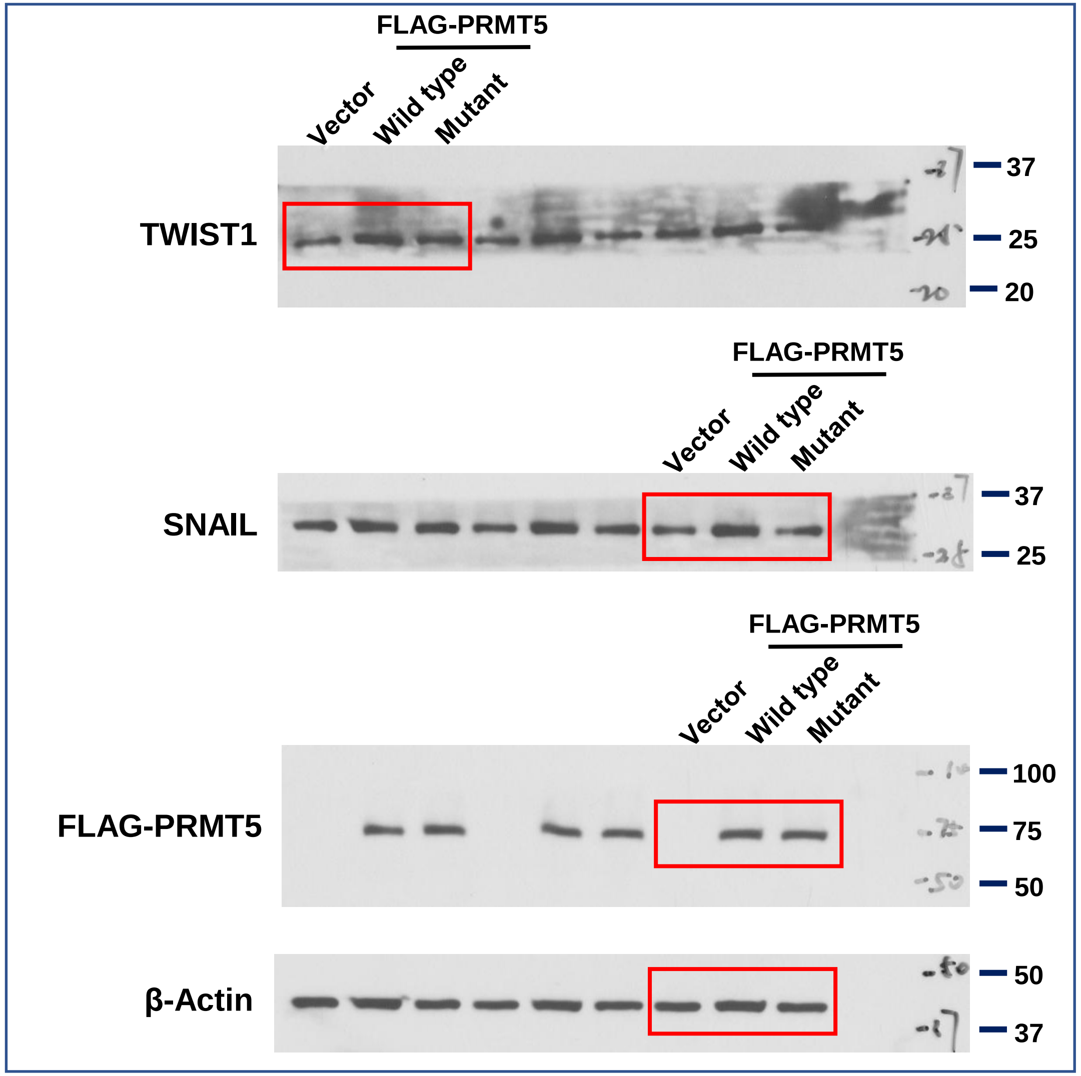
FLAG-PRMT5
Vector
Wild type
Mutant
TWIST1
37
25
20
SNAIL
37
25
FLAG-PRMT5
100
75
50
β-Actin
50
37

S1a

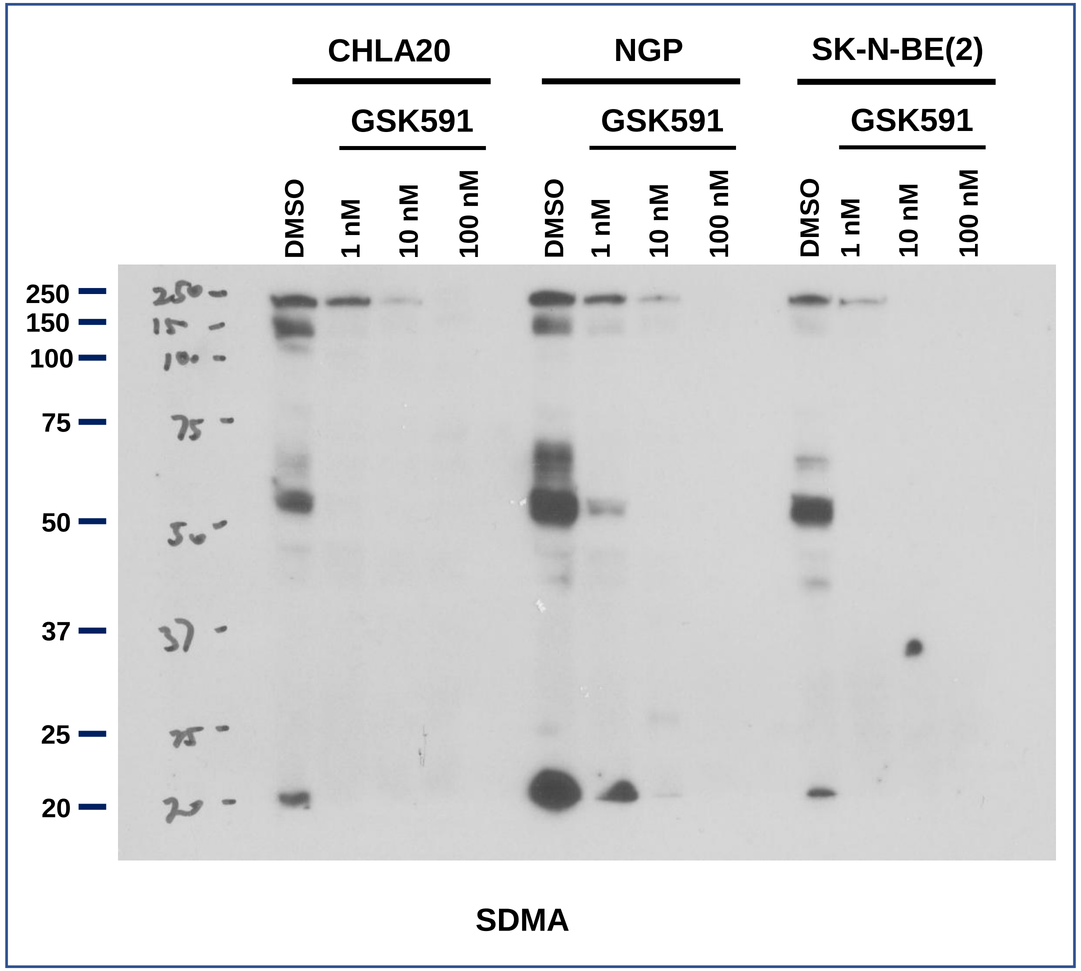
CHLA20
NGP
SK-N-BE(2)
GSK591
GSK591
GSK591
DMSO
1 nM
10 nM
100 nM
DMSO
1 nM
10 nM
100 nM
DMSO
1 nM
10 nM
100 nM
250
150
100
75
50
37
25
20
SDMA

S1b

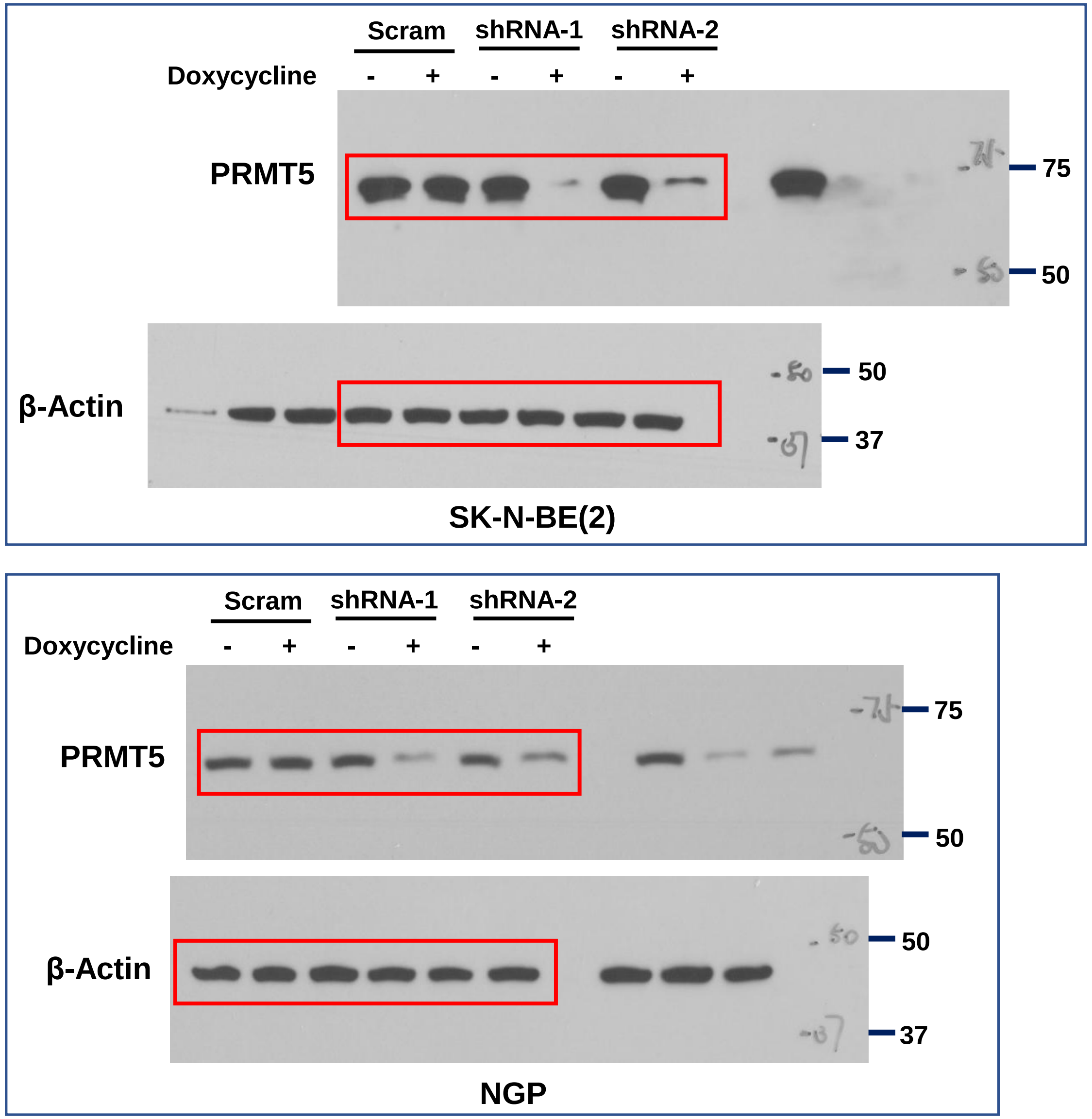
Scram
shRNA-1
shRNA-2
Doxycycline
- + - + - +
PRMT5
75
50
β-Actin
50
37
SK-N-BE(2)
Scram
shRNA-1
shRNA-2
Doxycycline
- + - + - +
PRMT5
75
50
β-Actin
50
37
NGP

S1c

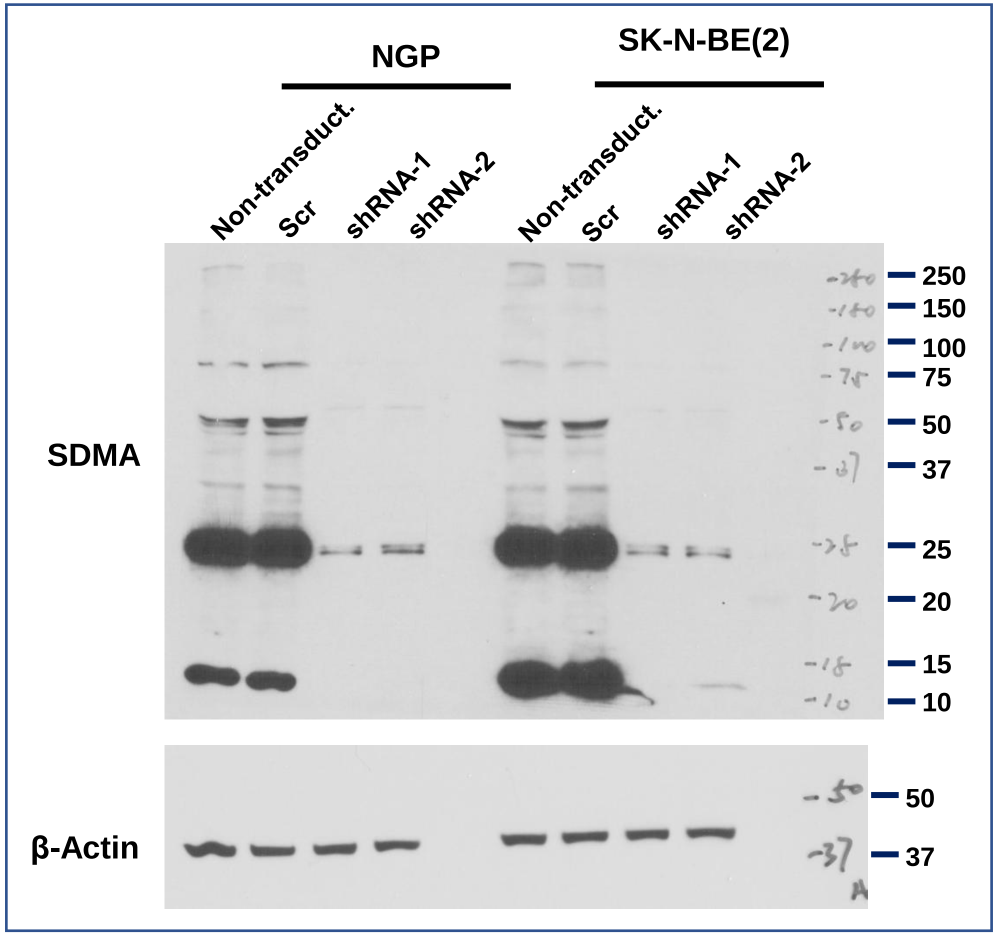
NGP
SK-N-BE(2)
Non-transduct.
Scr
shRNA-1
shRNA-2
Non-transduct.
Scr
shRNA-1
shRNA-2
SDMA
250
150
100
75
50
37
25
20
15
10
β-Actin
50
37

S1d

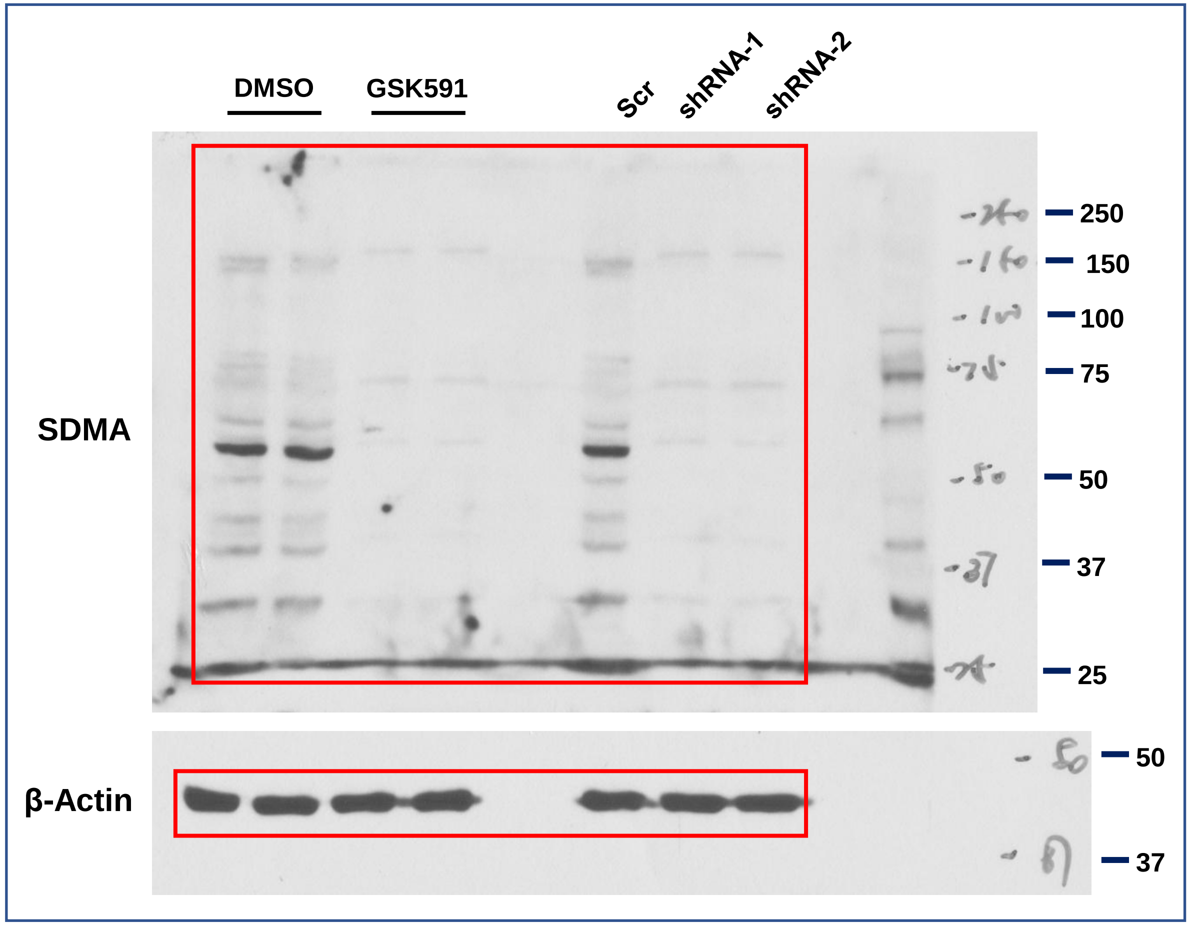
DMSO
GSK591
Scr
shRNA-1
shRNA-2
SDMA
250
150
100
75
50
37
25
β-Actin
50
37

S2b

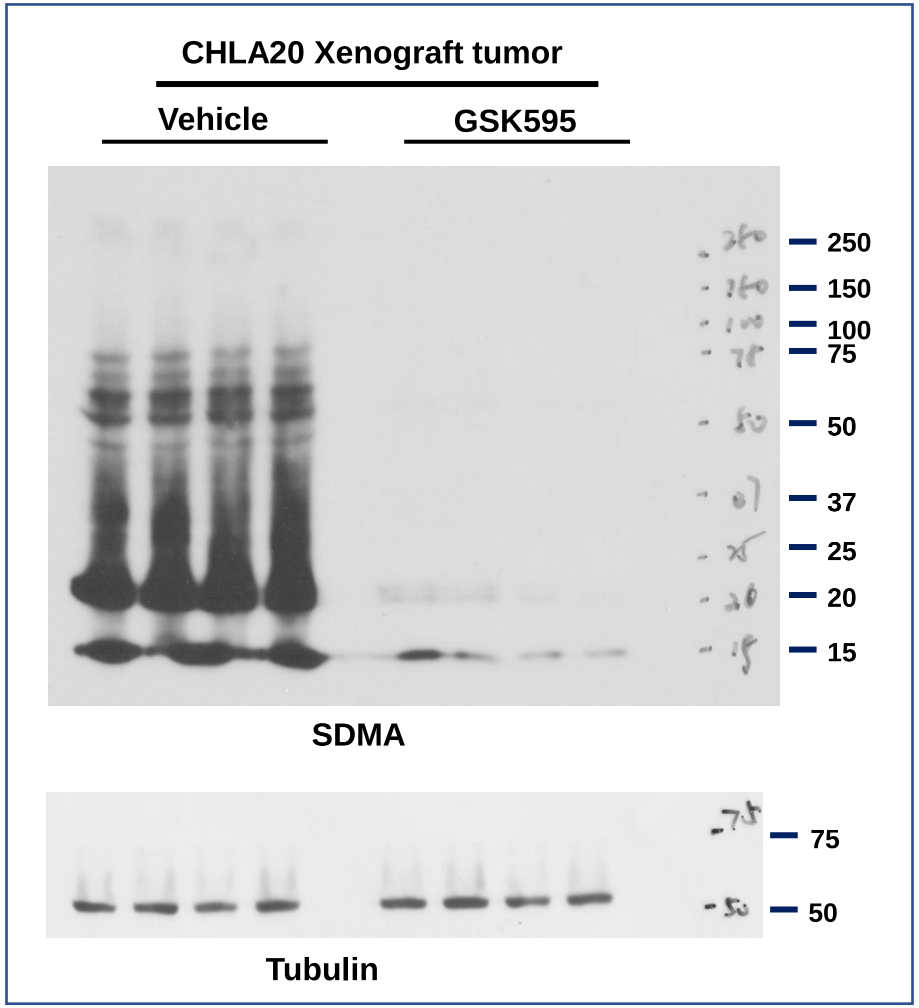
CHLA20 Xenograft tumor
Vehicle
GSK595
250
150
100
75
50
37
25
20
15
SDMA
75
50
Tubulin

S2c

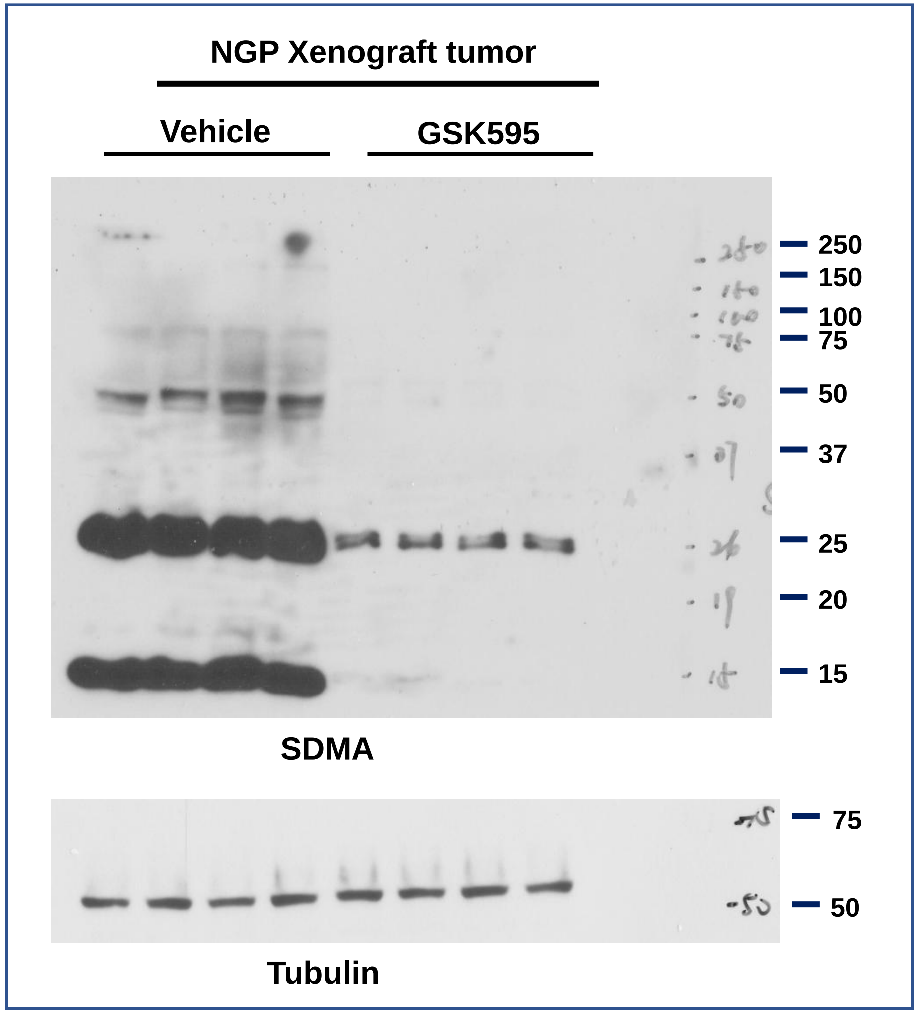
NGP Xenograft tumor
Vehicle
GSK595
250
150
100
75
50
37
25
20
15
SDMA
75
50
Tubulin

S3b

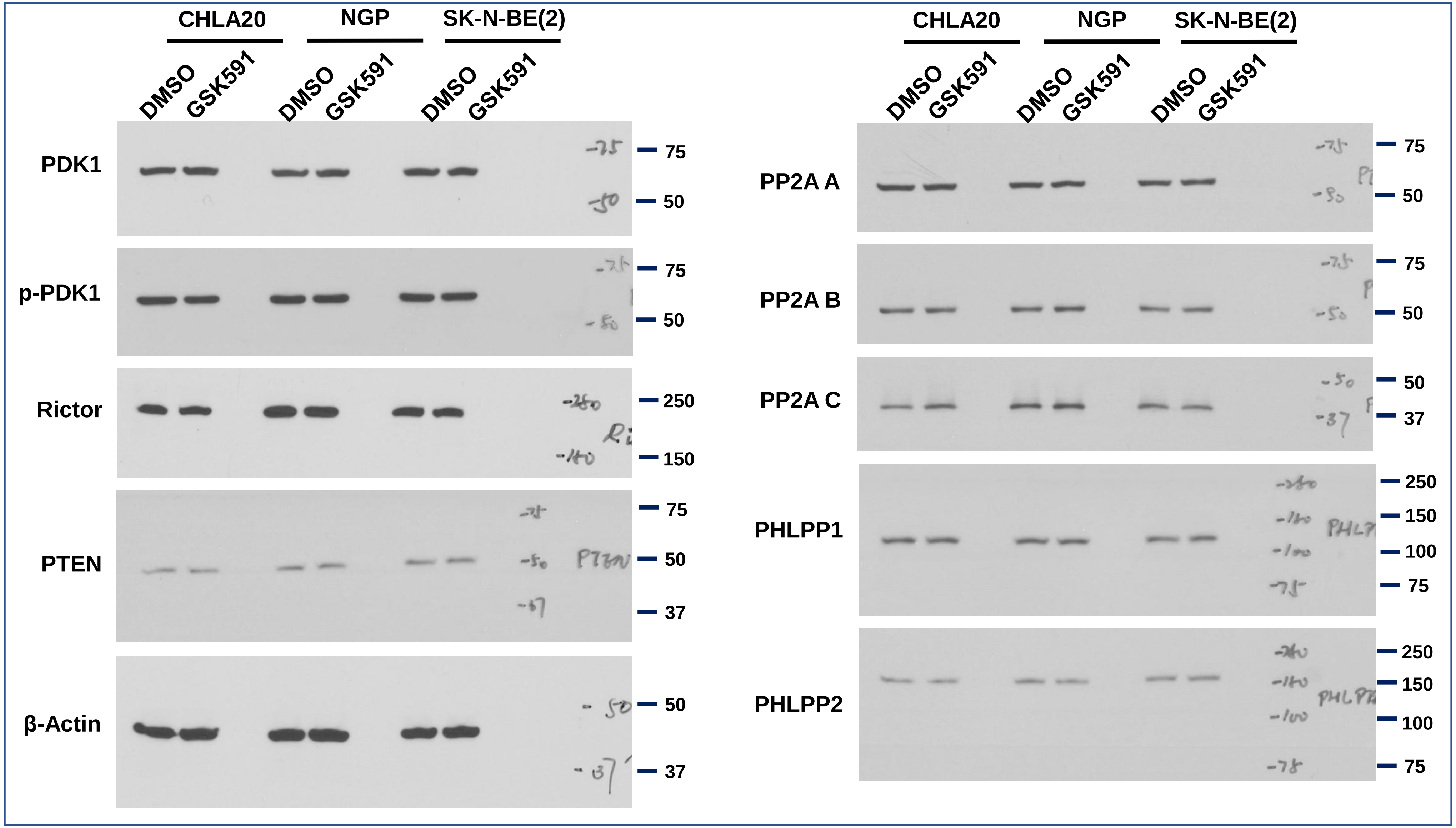
CHLA20
NGP
SK-N-BE(2)
DMSO
GSK591
PDK1
p-PDK1
Rictor
PTEN
β-Actin
PP2A A
PP2A B
PP2A C
PHLPP1
PHLPP2
75
50
250
150
37
100

S3b continue

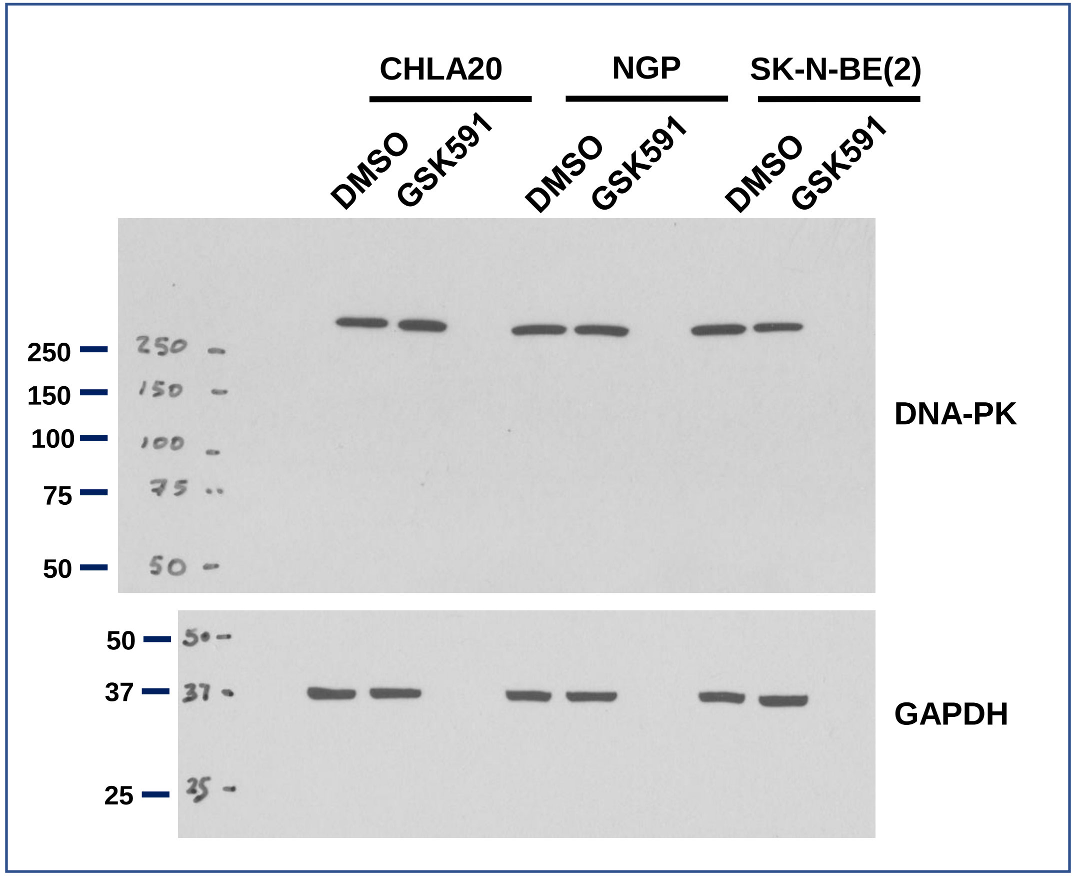

S3d

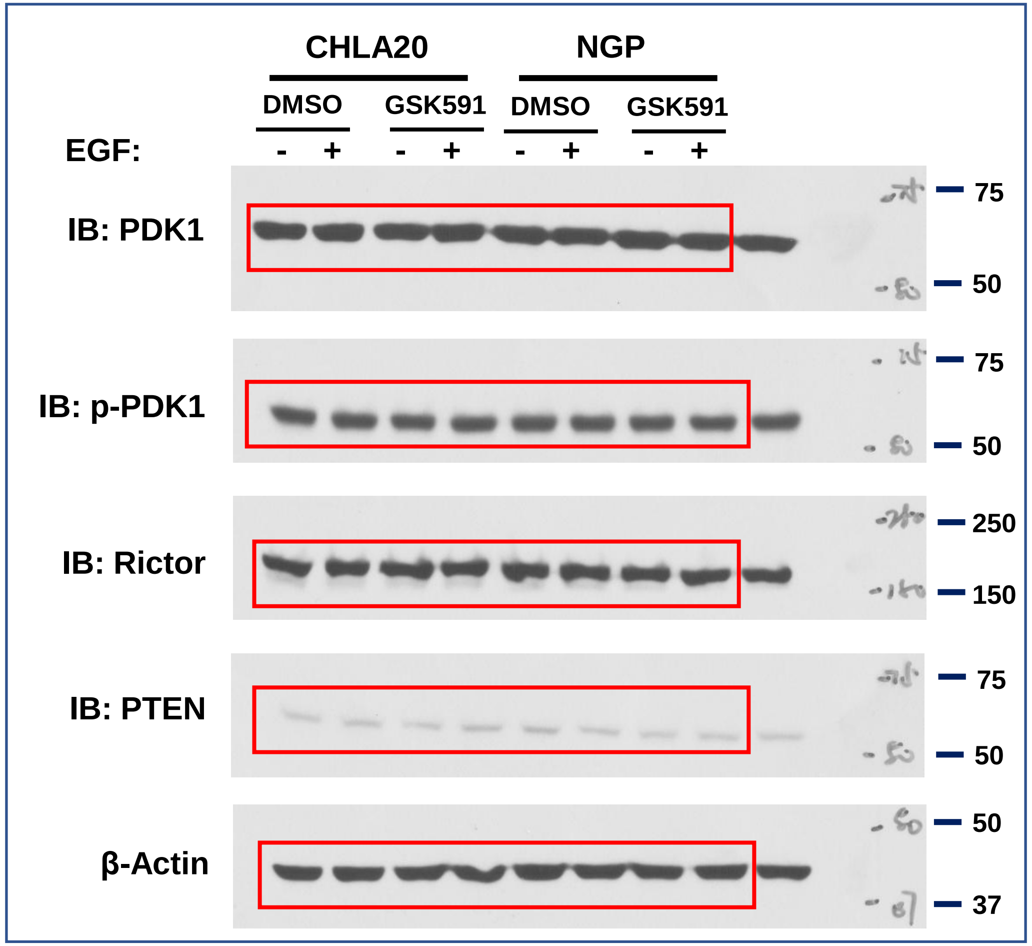

S3e

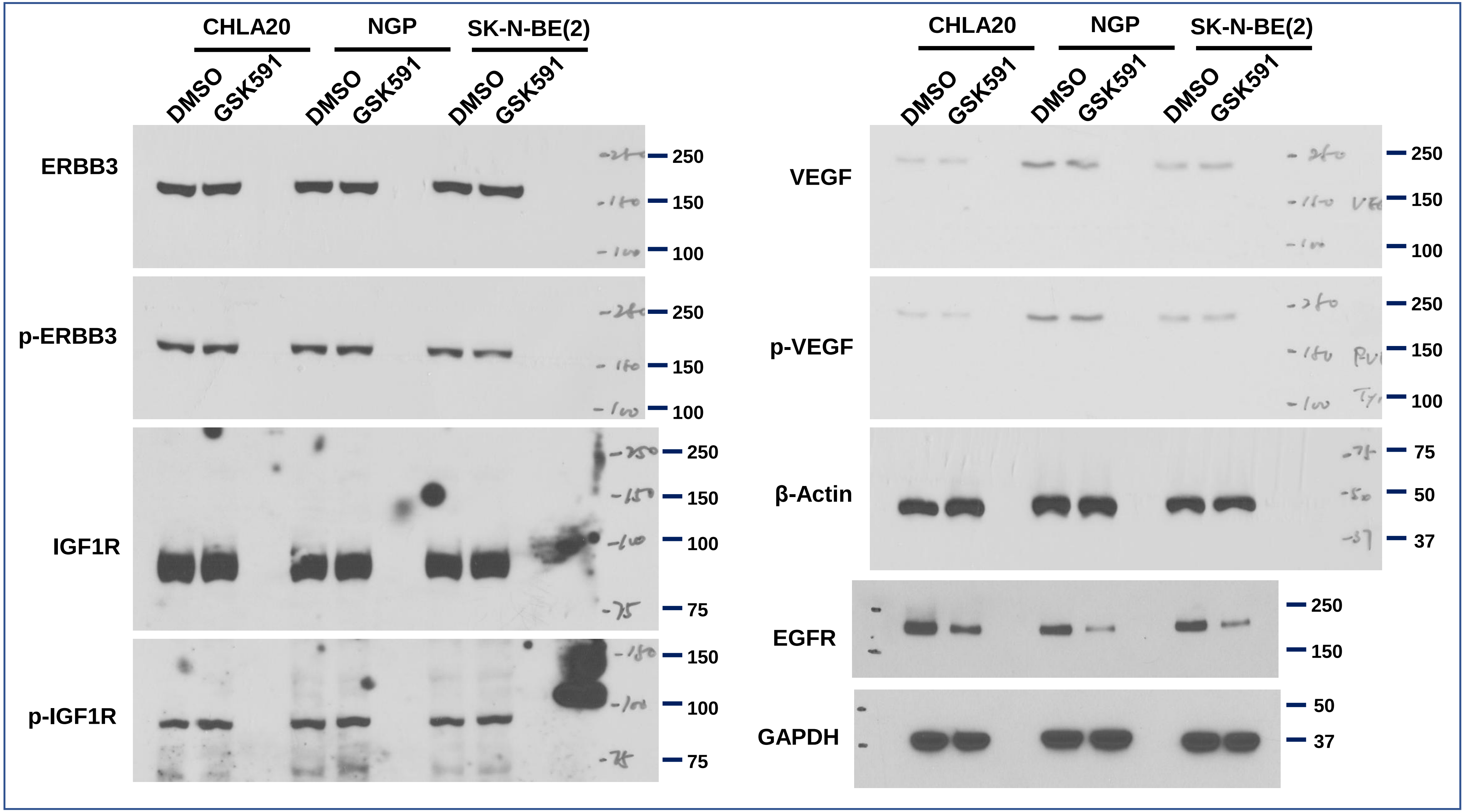

CHLA20
NGP
SK-N-BE(2)
DMSO
GSK591
ERBB3
p-ERBB3
IGF1R
p-IGF1R
VEGF
p-VEGF
β-Actin
EGFR
GAPDH
250
150
100
75
50
37

S3f

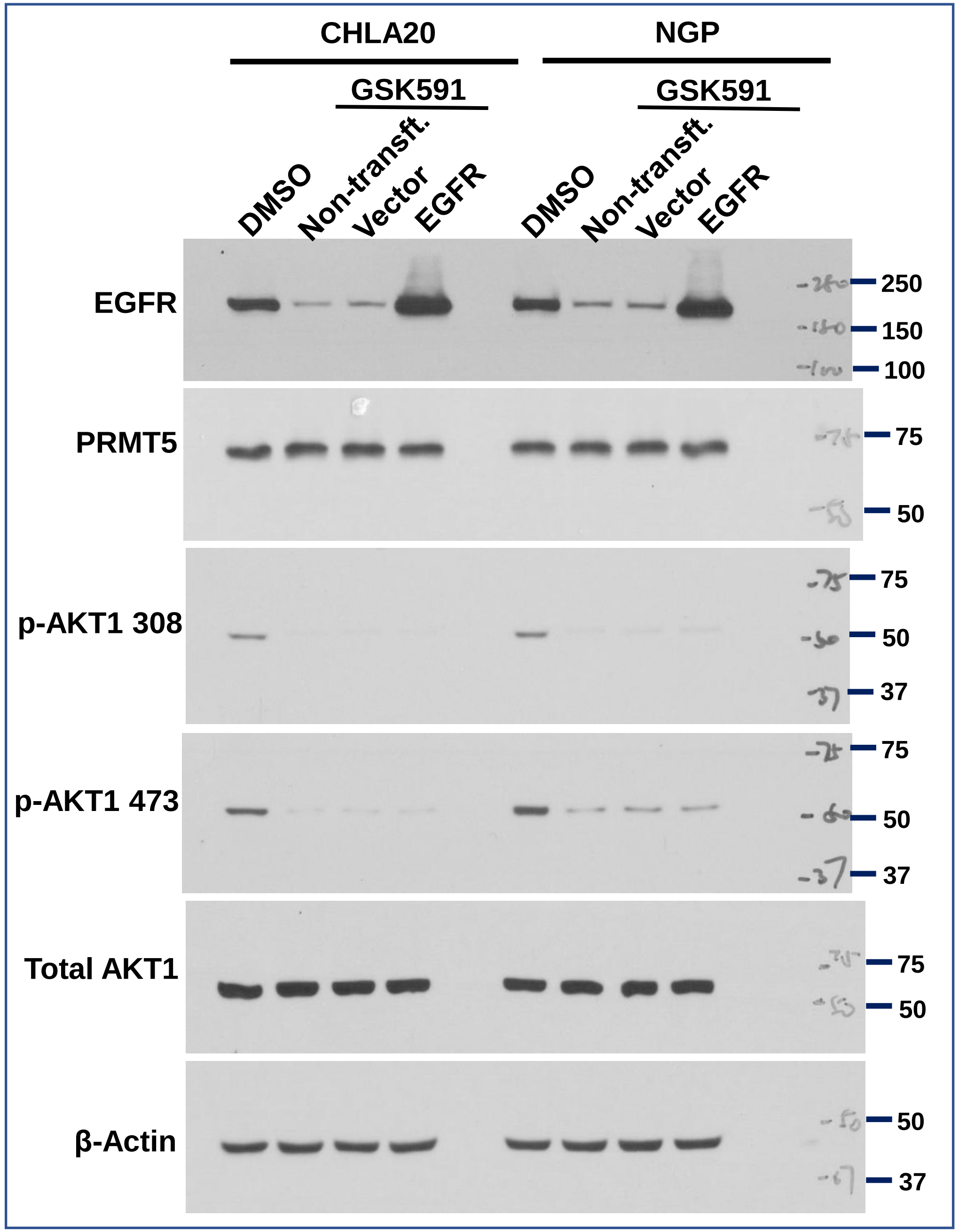

CHLA20
NGP
GSK591
GSK591
DMSO
Non-transft.
Vector
EGFR
DMSO
Non-transft.
Vector
EGFR
EGFR
250
150
100
PRMT5
75
50
p-AKT1 308
75
50
37
p-AKT1 473
75
50
37
Total AKT1
75
50
β-Actin
50
37

S3g

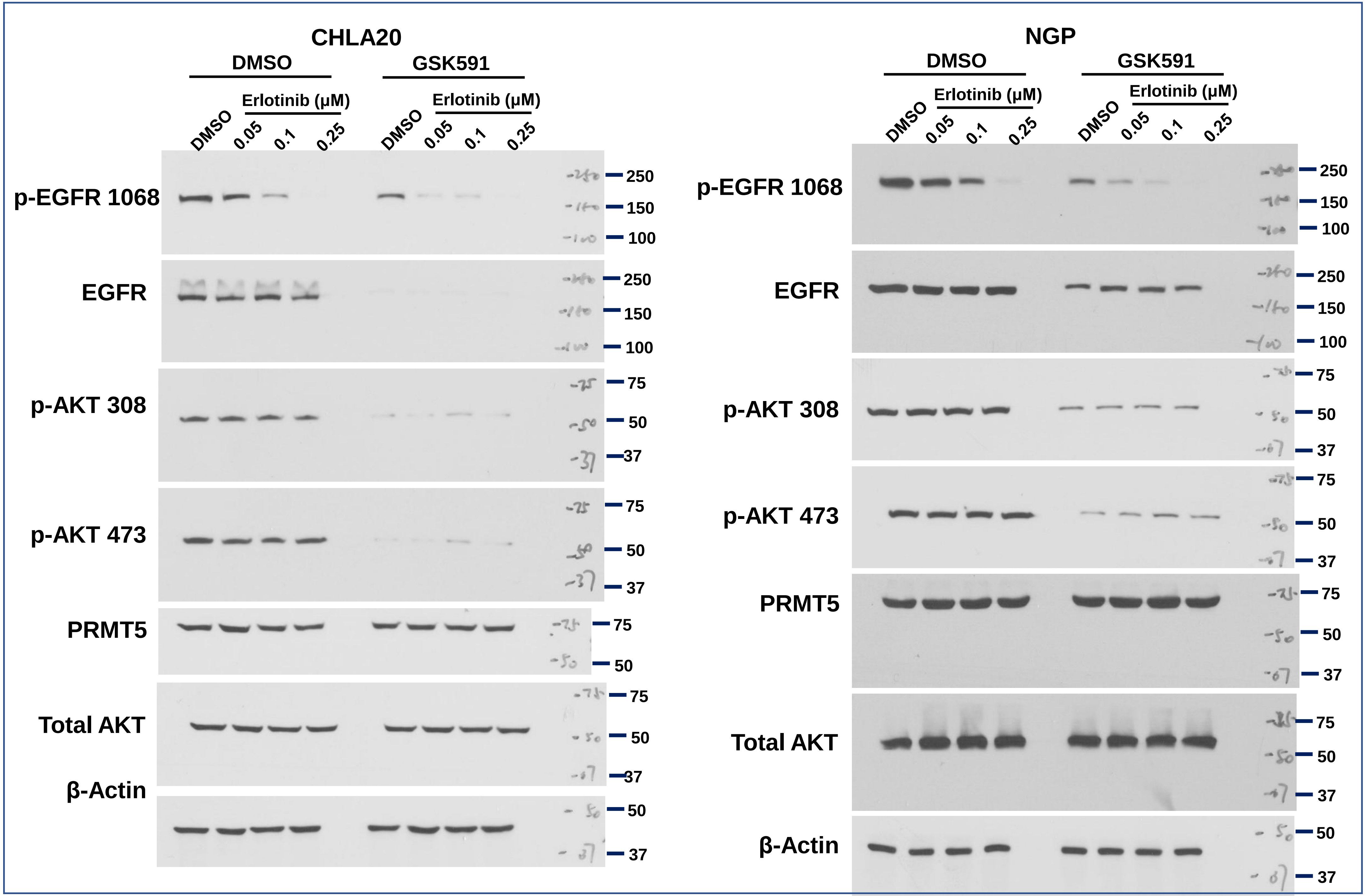
CHLA20
DMSO
GSK591
Erlotinib (μM)
DMSO
0.05
0.1
0.25
Erlotinib (μM)
DMSO
0.05
0.1
0.25
p-EGFR 1068
EGFR
p-AKT 308
p-AKT 473
PRMT5
Total AKT
β-Actin
250
150
100
75
50
37
NGP
DMSO
GSK591
Erlotinib (μM)
DMSO
0.05
0.1
0.25
Erlotinib (μM)
DMSO
0.05
0.1
0.25
p-EGFR 1068
EGFR
p-AKT 308
p-AKT 473
PRMT5
Total AKT
β-Actin

S4a

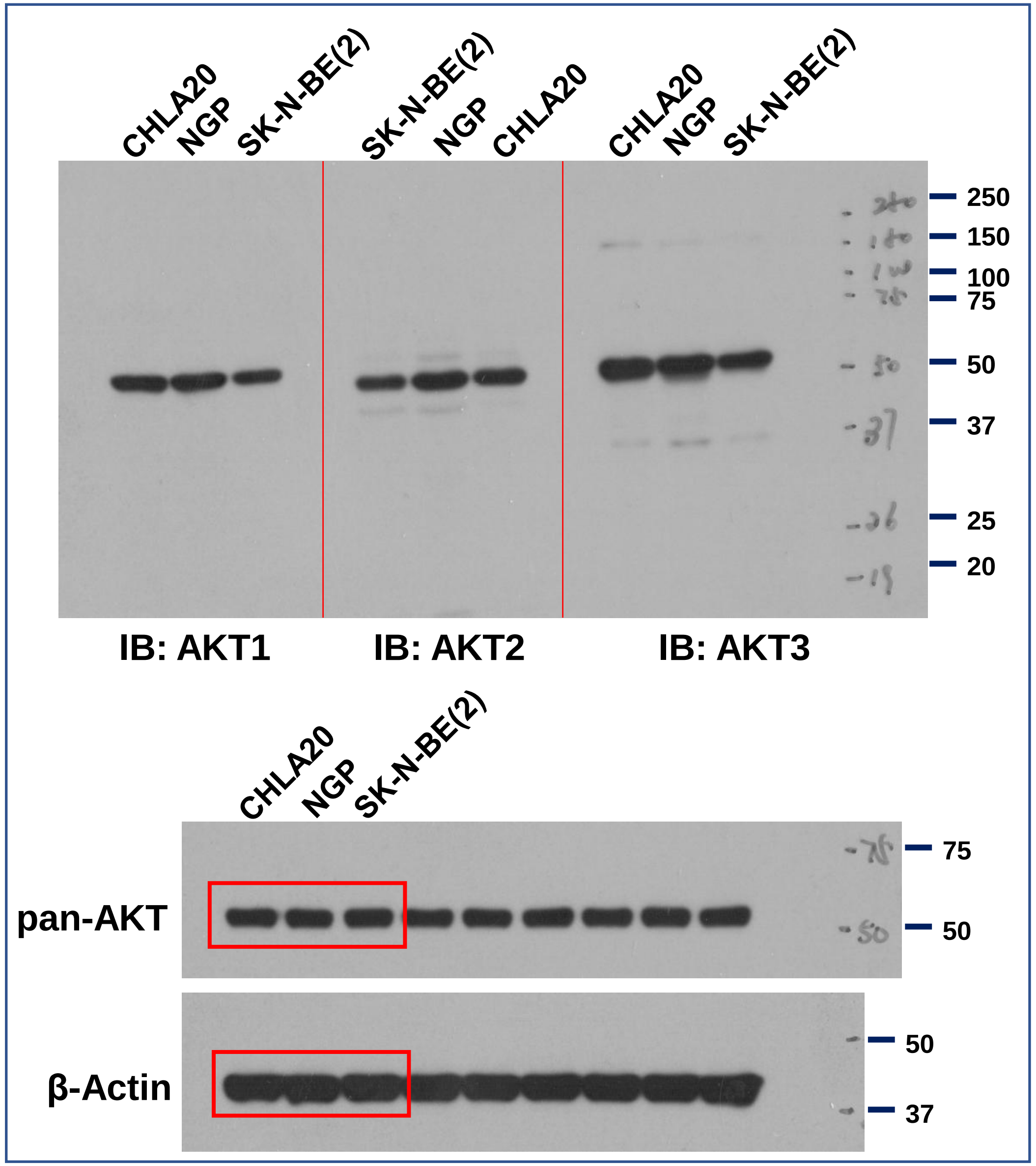
CHLA20
NGP
SK-N-BE(2)
SK-N-BE(2)
NGP
CHLA20
CHLA20
NGP
SK-N-BE(2)
250
150
100
75
50
37
25
20
IB: AKT1
IB: AKT2
IB: AKT3
CHLA20
NGP
SK-N-BE(2)
pan-AKT
75
50
β-Actin
50
37

S4b

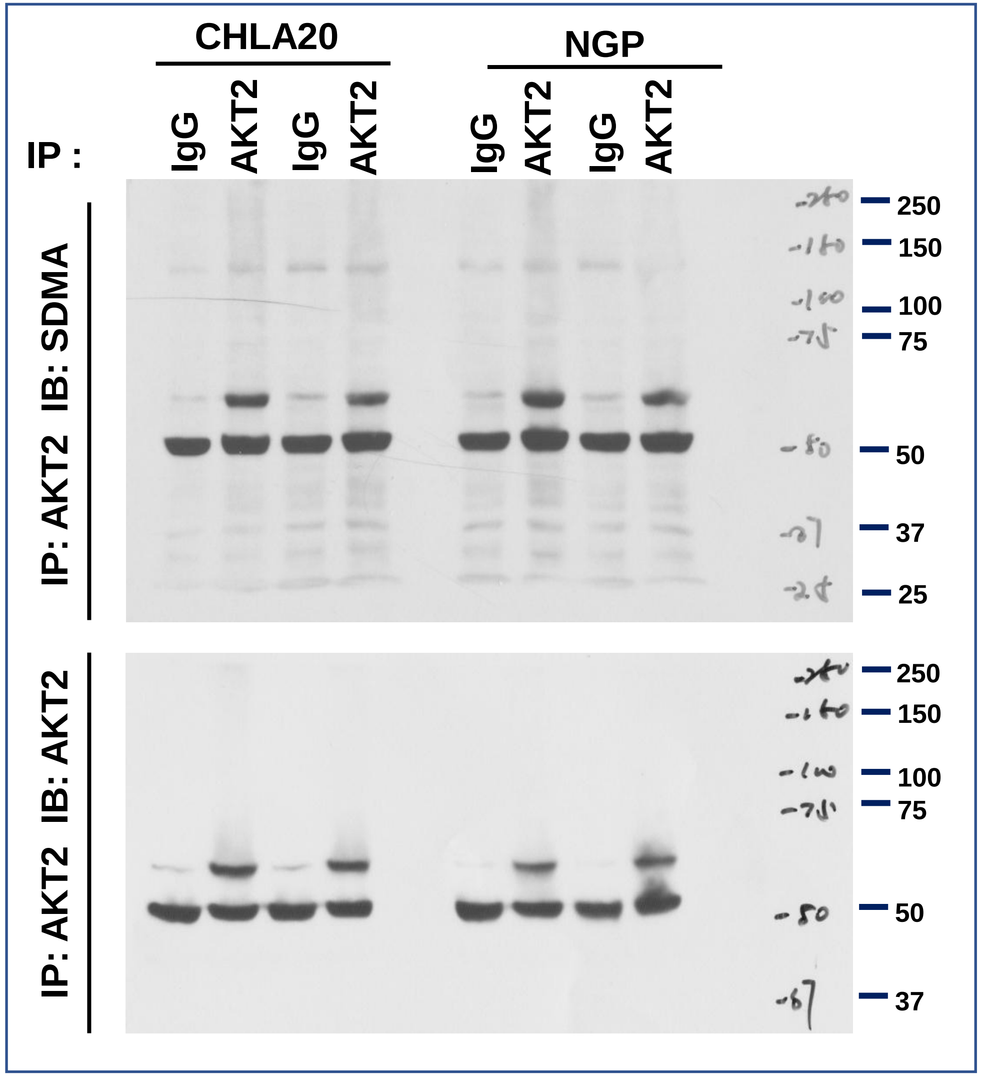
CHLA20
NGP
IP :
IgG
AKT2
IgG
AKT2
IgG
AKT2
IgG
AKT2
IP: AKT2 IB: SDMA
250
150
100
75
50
37
25
IP: AKT2 IB: AKT2
250
150
100
75
50
37

S4c

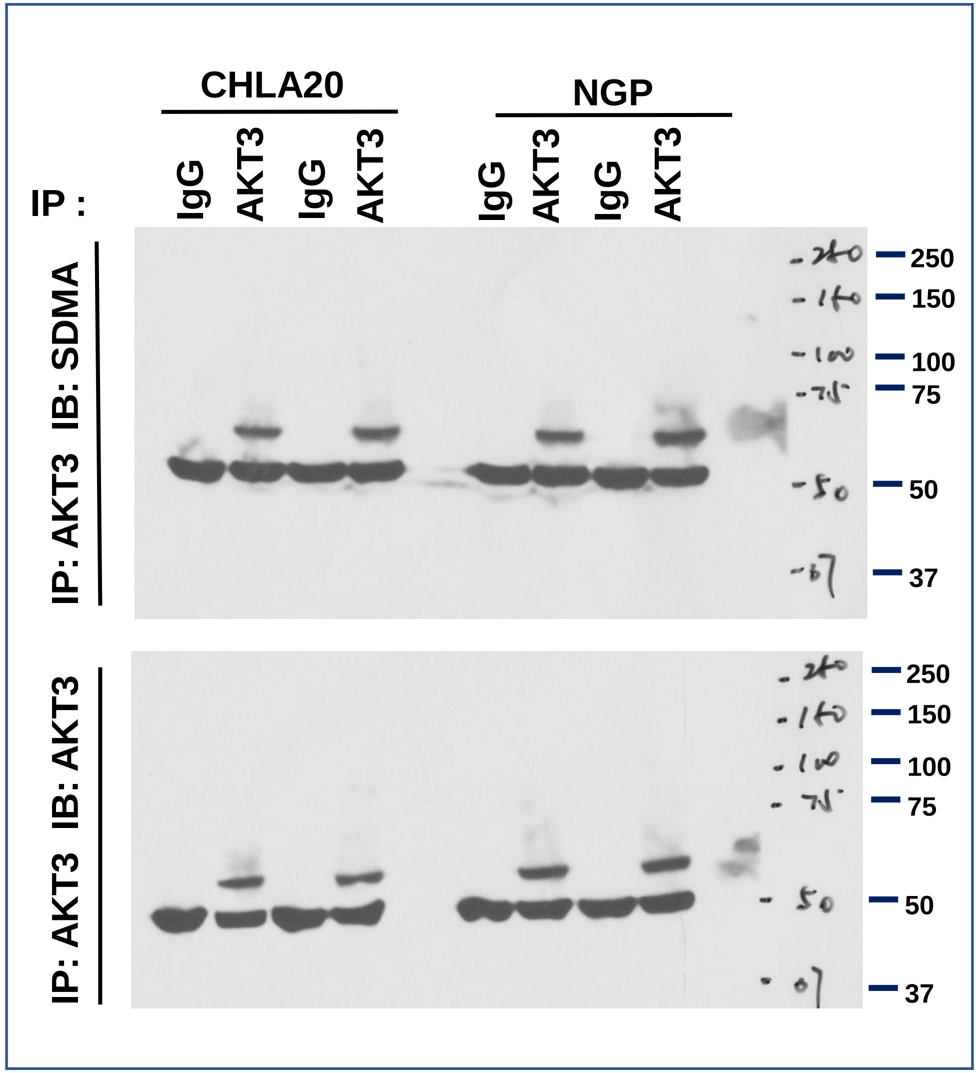
CHLA20
NGP
IP :
IgG
AKT3
IgG
AKT3
IgG
AKT3
IgG
AKT3
IP: AKT3 IB: SDMA
250
150
100
75
50
37
IP: AKT3 IB: AKT3
250
150
100
75
50
37

S4d

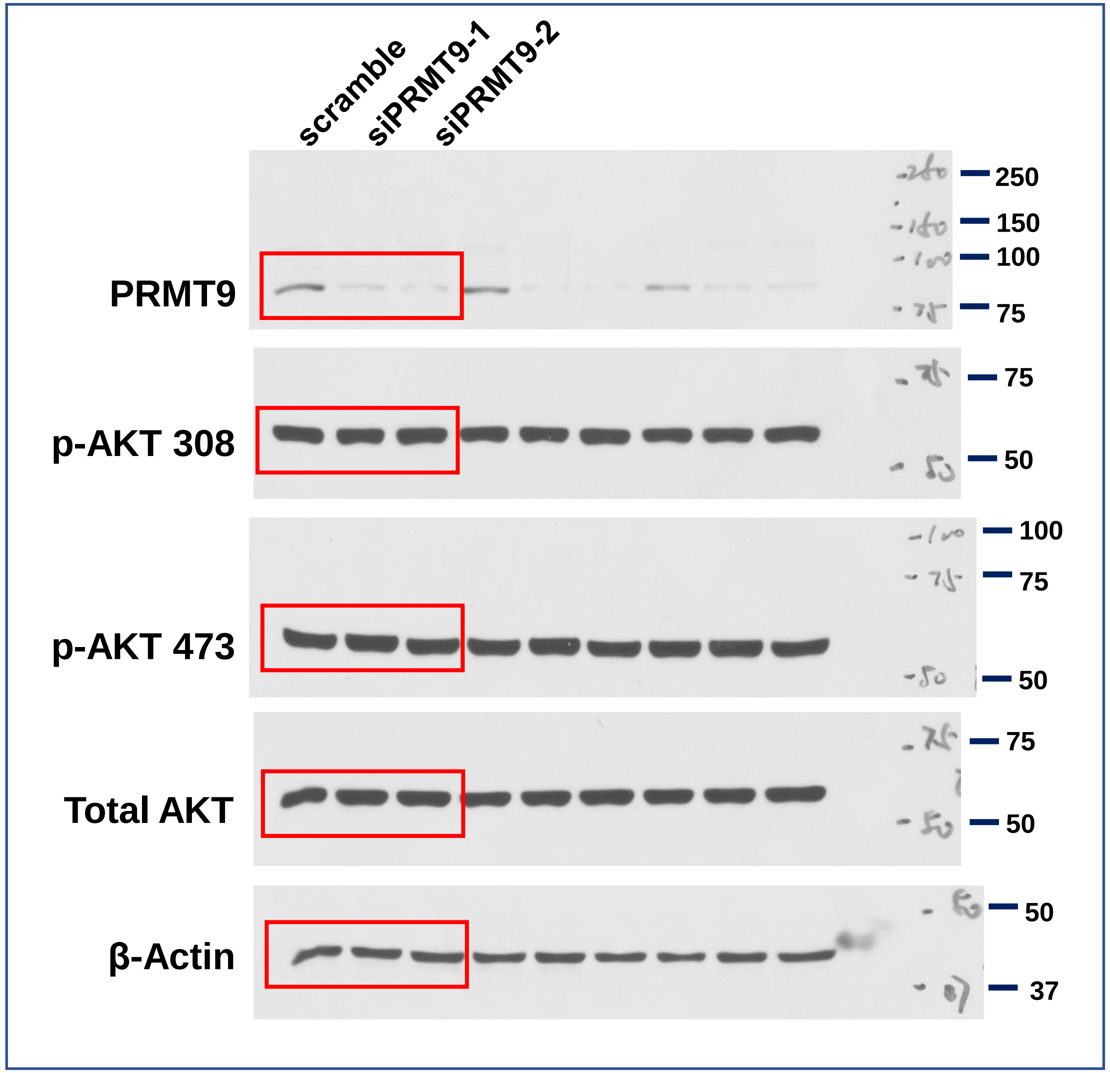
scramble
siPRMT9-1
siPRMT9-2
PRMT9
250
150
100
75
p-AKT 308
75
50
p-AKT 473
100
75
50
Total AKT
75
50
β-Actin
50
37

S5e

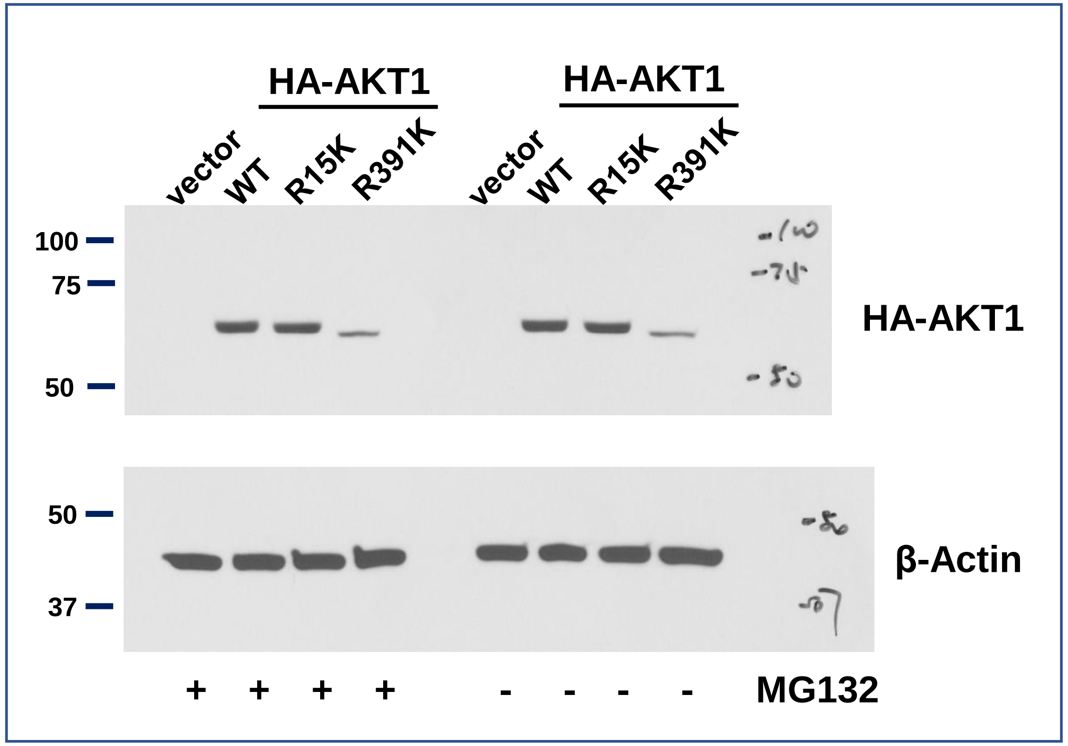
HA-AKT1
HA-AKT1
vector
WT
R15K
R391K
vector
WT
R15K
R391K
100
75
50
HA-AKT1
50
37
β-Actin
+ + + + - - - - MG132
