# Supplementary material for: PRMT5 activates AKT via methylation to promote tumor metastasis
Source: Nat Commun. 2022 Jul 8;13:3955. doi: 10.1038/s41467-022-31645-1 (PMC9270419; doi:10.1038/s41467-022-31645-1)
Supplement: Supplementary file 3 — Reporting Summary [file 41467_2022_31645_MOESM3_ESM.pdf]

nature research

Corresponding author(s): Joae Q. Wu
Last updated by author(s): May 28, 2022

Reporting Summary

Nature Research wishes to improve the reproducibility of the work that we publish. This form provides structure for consistency and transparency in reporting. For further information on Nature Research policies, see our Editorial Policies and the Editorial Policy Checklist.

Statistics

For all statistical analyses, confirm that the following items are present in the figure legend, table legend, main text, or Methods section.

n/a	Confirmed	
☐	☒	The exact sample size (*n*) for each experimental group/condition, given as a discrete number and unit of measurement
☐	☒	A statement on whether measurements were taken from distinct samples or whether the same sample was measured repeatedly
☐	☒	The statistical test(s) used AND whether they are one- or two-sided<br>*Only common tests should be described solely by name; describe more complex techniques in the Methods section.*
☒	☐	A description of all covariates tested
☒	☐	A description of any assumptions or corrections, such as tests of normality and adjustment for multiple comparisons
☐	☒	A full description of the statistical parameters including central tendency (e.g. means) or other basic estimates (e.g. regression coefficient) AND variation (e.g. standard deviation) or associated estimates of uncertainty (e.g. confidence intervals)
☐	☒	For null hypothesis testing, the test statistic (e.g. *F*, *t*, *r*) with confidence intervals, effect sizes, degrees of freedom and *P* value noted<br>*Give P values as exact values whenever suitable.*
☒	☐	For Bayesian analysis, information on the choice of priors and Markov chain Monte Carlo settings
☒	☐	For hierarchical and complex designs, identification of the appropriate level for tests and full reporting of outcomes
☐	☒	Estimates of effect sizes (e.g. Cohen's *d*, Pearson's *r*), indicating how they were calculated

Our web collection on statistics for biologists contains articles on many of the points above.

Software and code

Policy information about availability of computer code

Data collection	BD FACSDiva or MACSQuant 10 for flow cytometry data acquisition;<br>HP LJ300-400 M375-M475 Scan Software for image data collection;<br>MTS assay was read in Synergy HT microplate reader using Gen5 software;<br>Live animals and ex vivo organs were imaged in IVIS Imaging System using Caliper Life Sciences Living Image® Software Version 3.2; ex vivo organs were photographed using iphone camera;<br>Fluorescence imaging was acquired by Zeiss flurorescence microscope using ZEN blue software and nikon ECLIPSE Ti2 confocal microscope;<br>Images for cell invasion and migration were taken by Canon digital camera mounted on an Olympus CK2 microscope.
Data analysis	The results presented in Figure 1b-d were analyzed using survminer 0.4.8. The RNA expression of PRMT5 in increasing grades of neuroblastoma was analyzed using R. Proteomic screening heatmaps were developed in GraphPad Prism 9.3.0. All statistical tests were calculated in GraphPad Prism, Microsoft Excel or as specified in figure legend. Cell invasion and migration images were analyzed in Image J 1.53k. Flowjo 10.7.1 or MACSQuantify 2.13.3 was used for flow cytometry analyses.

For manuscripts utilizing custom algorithms or software that are central to the research but not yet described in published literature, software must be made available to editors and reviewers. We strongly encourage code deposition in a community repository (e.g. GitHub). See the Nature Research guidelines for submitting code & software for further information.

Data

Policy information about availability of data

All manuscripts must include a data availability statement. This statement should provide the following information, where applicable:

- Accession codes, unique identifiers, or web links for publicly available datasets
- A list of figures that have associated raw data
- A description of any restrictions on data availability

All data generated or analysed during this study are included in this published article (and its supplementary information files).
-The RNA-seq of increasing grades of neuroblastoma was from the GSE49711 RNA-seq data series.
-Results presented in Figure 1b-d were generated in R2 database. The gene expression and clinical data from 161 neuroblastoma patients were downloaded from the TARGET database [https://ocg.cancer.gov/programs/target/projects/neuroblastoma]. Three sets of microarray data from neuroblastoma patient cohorts were downloaded from R2 database (R2: Genomics Analysis and Visualization Platform [http://r2.amc.nl]).

Field-specific reporting

Please select the one below that is the best fit for your research. If you are not sure, read the appropriate sections before making your selection.

☒ Life sciences ☐ Behavioural & social sciences ☐ Ecological, evolutionary & environmental sciences

For a reference copy of the document with all sections, see nature.com/documents/nr-reporting-summary-flat.pdf

Life sciences study design

All studies must disclose on these points even when the disclosure is negative.

Sample size	The number of patients depended on availability of patient data in the database. The number of mice was chosen based on the minimal number of animals required to get statistic power. The proteomic screening was performed in samples collected from two independent biological experiments. For culture based experiments, sample sizes were determined based on pilot experiments and commonly used sample sizes in comparable publications within the field. Sample size used for analysis was 3- 6 as stated in the figure legends.
Data exclusions	No data were excluded from analysis.
Replication	To verify the reproducibility of our findings, experiments were performed using at least three biological replicates, unless clearly stated otherwise in the figure legends. All attempts at replication were successful. Proteomic screening and immunofluorescence were performed in biological independent duplicates with similar results. Other data presented in the manuscript were carried out in at least triplicates, as specified in Figure legends and Supplementary legends. Each replicate represents an independent biological experiment. All statistical analyses and figures pertain to pooled results from all these replicates.
Randomization	In animal study, mice with similar tumor size determined by bioluminascent signal were randomized to different treatment group. For cell culture experiments, cells were split, plated in culture vessels, and then treated with DMSO or drugs. Because control and treatment groups were derived from the same cell line, no randomization could be performed.
Blinding	Investigators who performed animal experiments were not blinded because they needed to prepare the drug freshly. But the Investigators were blinded during the sample collection and data analysis (live animal imaging, ex vivo organ imaging, tumor mass collection, and FACS). The in vitro cell culture experiments were not performed in a blinding manner because the same investigator performed the cell culture, treatment and processing. But independent experiments were conducted by different investigators.

Reporting for specific materials, systems and methods

We require information from authors about some types of materials, experimental systems and methods used in many studies. Here, indicate whether each material, system or method listed is relevant to your study. If you are not sure if a list item applies to your research, read the appropriate section before selecting a response.

Materials & experimental systems

n/a	Involved in the study	
☐	☒	Antibodies
☐	☒	Eukaryotic cell lines
☒	☐	Palaeontology and archaeology
☐	☒	Animals and other organisms
☒	☐	Human research participants
☐	☒	Clinical data
☒	☐	Dual use research of concern

Methods

n/a	Involved in the study	
☒	☐	ChIP-seq
☐	☒	Flow cytometry
☒	☐	MRI-based neuroimaging

Antibodies

Antibodies used

The following antibodies were used in this study (all commercially available):
From Cell Signaling Technology:
1: Akt Antibody (#9272, 1:1000)

2: Akt1 (C73H10) Rabbit mAb (#2938, 1:1000/1:50)

3: Akt2 (D6G4) Rabbit mAb (#3063, 1:1000/1:50)

4: Akt3 (E1Z3W) Rabbit mAb (#14982, 1:1000/1:50)

5: Phospho-Akt (Ser473) (D9E) XP® Rabbit mAb (#4060, 1:1000/1:300)

6: Phospho-Akt (Thr308) (D25E6) XP® Rabbit mAb (#13038, 1:1000/1:500)

7: Phospho-Akt1 (Ser473) (D7F10) XP® Rabbit mAb (Akt1 Specific) (#9018, 1:1000)

8: Phospho-Akt2 (Ser474) (D3H2) Rabbit mAb (Akt2 Specific) (#8599, 1:1000)

9: Symmetric Di-Methyl Arginine Motif [sdme-RG] MultiMab™ Rabbit mAb mix (#13222, 1:1000)

10: DNA-PKcs (E6U3A) Rabbit mAb (#38168, 1:1000)

11: EGF Receptor (D38B1) XP® Rabbit mAb (#4267, 1:1000)

12: Phospho-EGF Receptor (Tyr1068) (D7A5) XP® Rabbit mAb (#3777, 1:1000)

13: HER3/ErbB3 (D22C5) XP® Rabbit mAb (#12708, 1:1000)

14: Phospho-HER3/ErbB3 (Tyr1289) (21D3) Rabbit mAb (#4791, 1:1000)

15: FGF Receptor 4 (D3B12) XP® Rabbit mAb (#8562, 1:1000)

16: VEGF Receptor 2 (D5B1) Rabbit mAb (#9698, 1:1000)

17: Phospho-VEGF Receptor 2 (Tyr1059) (D5A6) Rabbit mAb (#3817, 1:1000)

18: IGF-I Receptor β (D23H3) XP® Rabbit mAb (#9750, 1:1000)

19: Phospho-IGF-I Receptor β (Tyr1316) Antibody (#28897, 1:1000)

20: GSK-3α (D80D1) Rabbit mAb (#4818, 1:1000)

21: GSK-3β (D5C5Z) XP® Rabbit mAb (#12456, 1:1000)

22: Phospho-GSK-3α (Ser21) (36E9) Rabbit mAb (#9316, 1:1000)

23: Phospho-GSK-3β (Ser9) (D85E12) XP® Rabbit mAb (#5558, 1:1000)

24: Phospho-(Ser/Thr) Phe Antibody (#9631, 1:1000)

25: Phospho-Tyrosine (P-Tyr-1000) MultiMab™ Rabbit mAb mix (#8954, 1:1000)

26: p75NTR (D4B3) XP® Rabbit mAb (#8238, 1:1000)

27: PDK1 Antibody (#3062, 1:1000)

28: PP2A A Subunit (81G5) Rabbit mAb (#2041, 1:1000)

29: PP2A B Subunit (100C1) Rabbit mAb (#2290, 1:1000)

30: PP2A C Subunit (52F8) Rabbit mAb (#2259, 1:1000)

31: PTEN (D4.3) XP® Rabbit mAb (#9188, 1:1000)

32: Rictor (D16H9) Rabbit mAb (#9476, 1:1000)

33: Phospho-Rictor (Thr1135) (D30A3) Rabbit mAb (#3806, 1:1000)

34: ZEB1 (E2G6Y) XP® Rabbit mAb (#70512, 1:1000)

35: Snail (C15D3) Rabbit mAb (#3879, 1:1000)

36: TWIST1 (E7E2G) Rabbit mAb (#69366, 1:1000)

37: Sin1 (D7G1A) Rabbit mAb (#12860, 1:1000)

38: Anti-rabbit IgG, HRP-linked Antibody (#7074, 1:2000)

From Santa Cruz Biotechnology:
39: Anti-PRMT5 Antibody (A-11): (sc-376937, 1:1000/1:50)

40: mouse anti-rabbit IgG-HRP: (sc-2357, 1:2000)

41: m-IgGκ BP-HRP: (sc-516102, 1:2000)

From Sigma:
42: Anti-dimethyl-Arginine Antibody, symmetric (SYM10) (#07-412, 1:1000)

43: ANTI-FLAG® antibody produced in rabbit (#F7425, 1:2000)

44: Anti-HA-Peroxidase, High Affinity (#12013819001, 1:2000)

From ThermoFisher
45: Goat anti-Rabbit IgG (H+L) Highly Cross-Adsorbed Secondary Antibody, Alexa Fluor™ Plus 488 (#A32731, 1:300)

46: Goat anti-Mouse IgG (H+L) Highly Cross-Adsorbed Secondary Antibody, Alexa Fluor™ 594 (#A11032, 1:300)

From Bethyl Laboratories
47: Anti-PRMT9 antibody (#A304-189A, 1:1000)

Validation

1: Akt Antibody
https://www.cellsignal.com/products/primary-antibodies/akt-antibody/9272?_=1653337063498&Ntt=9272&tahead=true

2: Akt1 (C73H10) Rabbit mAb
https://www.cellsignal.com/products/primary-antibodies/akt1-c73h10-rabbit-mab/2938?_=1653337070682&Ntt=2938&tahead=true

3: Akt2 (D6G4) Rabbit mAb
https://www.cellsignal.com/products/primary-antibodies/akt2-d6g4-rabbit-mab/3063?_=1653337334247&Ntt=3063&tahead=true

4: Akt3 (E1Z3W) Rabbit mAb
https://www.cellsignal.com/products/primary-antibodies/akt3-e1z3w-rabbit-mab/14982?_=1653337442304&Ntt=14982&tahead=true

5: Phospho-Akt (Ser473) (D9E) XP® Rabbit mAb
https://www.cellsignal.com/products/primary-antibodies/phospho-akt-ser473-d9e-xp-rabbit-mab/4060?_=1653337516456&Ntt=4060&tahead=true

6: Phospho-Akt (Thr308) (D25E6) XP® Rabbit mAb
https://www.cellsignal.com/products/primary-antibodies/phospho-akt-thr308-d25e6-xp-rabbit-mab/13038?_=1653337580020&Ntt=13038&tahead=true

7: Phospho-Akt1 (Ser473) (D7F10) XP® Rabbit mAb (Akt1 Specific)
https://www.cellsignal.com/products/primary-antibodies/phospho-akt1-ser473-d7f10-xp-rabbit-mab-akt1-specific/9018?_=1653337646408&Ntt=9018&tahead=true

8: Phospho-Akt2 (Ser474) (D3H2) Rabbit mAb (Akt2 Specific)
https://www.cellsignal.com/products/primary-antibodies/phospho-akt2-ser474-d3h2-rabbit-mab-akt2-specific/8599?_=1653337713870&Ntt=8599&tahead=true

9: Symmetric Di-Methyl Arginine Motif [sdme-RG] MultiMab™ Rabbit mAb mix
https://www.cellsignal.com/products/primary-antibodies/symmetric-di-methyl-arginine-motif-sdme-rg-multimab-rabbit-mab-

mix/13222

10: DNA-PKcs (E6U3A) Rabbit mAb
https://www.cellsignal.com/products/primary-antibodies/dna-pkcs-e6u3a-rabbit-mab/38168?_=1653337906988&Ntt=38168&tahead=true

11: EGF Receptor (D38B1) XP® Rabbit mAb
https://www.cellsignal.com/products/primary-antibodies/egf-receptor-d38b1-xp-rabbit-mab/4267?_=1653337954846&Ntt=4267&tahead=true

12: Phospho-EGF Receptor (Tyr1068) (D7A5) XP® Rabbit mAb (
https://www.cellsignal.com/products/primary-antibodies/phospho-egf-receptor-tyr1068-d7a5-xp-rabbit-mab/3777?_=1653338027107&Ntt=3777&tahead=true

13: HER3/ErbB3 (D22C5) XP® Rabbit mAb
https://www.cellsignal.com/products/primary-antibodies/her3-erbb3-d22c5-xp-rabbit-mab/12708?_=1653338073855&Ntt=12708&tahead=true

14: Phospho-HER3/ErbB3 (Tyr1289) (21D3) Rabbit mAb
https://www.cellsignal.com/products/primary-antibodies/phospho-her3-erbb3-tyr1289-21d3-rabbit-mab/4791

15: FGF Receptor 4 (D3B12) XP® Rabbit mAb
https://www.cellsignal.com/products/primary-antibodies/fgf-receptor-4-d3b12-xp-rabbit-mab/8562

16: VEGF Receptor 2 (D5B1) Rabbit mAb
https://www.cellsignal.com/products/primary-antibodies/vegf-receptor-2-d5b1-rabbit-mab/9698?_=1653338294467&Ntt=9698&tahead=true

17: Phospho-VEGF Receptor 2 (Tyr1059) (D5A6) Rabbit mAb
https://www.cellsignal.com/products/primary-antibodies/phospho-vegf-receptor-2-tyr1059-d5a6-rabbit-mab/3817?_=1653338262889&Ntt=3817&tahead=true

18: IGF-I Receptor β (D23H3) XP® Rabbit mAb
https://www.cellsignal.com/products/primary-antibodies/igf-i-receptor-b-d23h3-xp-rabbit-mab/9750?_=1653338327777&Ntt=9750&tahead=true

19: Phospho-IGF-I Receptor β (Tyr1316) Antibody
https://www.cellsignal.com/products/primary-antibodies/phospho-igf-i-receptor-b-tyr1316-antibody/28897?_=1653338361192&Ntt=28897&tahead=true

20: GSK-3α (D80D1) Rabbit mAb
https://www.cellsignal.com/products/primary-antibodies/gsk-3a-d80d1-rabbit-mab/4818?_=1653338430119&Ntt=4818&tahead=true

21: GSK-3β (D5C5Z) XP® Rabbit mAb
https://www.cellsignal.com/products/primary-antibodies/gsk-3b-d5c5z-xp-rabbit-mab/12456?_=1653338477934&Ntt=12456&tahead=true

22: Phospho-GSK-3α (Ser21) (36E9) Rabbit mAb
https://www.cellsignal.com/products/primary-antibodies/phospho-gsk-3a-ser21-36e9-rabbit-mab/9316?_=1653338532134&Ntt=9316&tahead=true

23: Phospho-GSK-3β (Ser9) (D85E12) XP® Rabbit mAb
https://www.cellsignal.com/products/primary-antibodies/phospho-gsk-3b-ser9-d85e12-xp-rabbit-mab/5558?_=1653338572061&Ntt=5558&tahead=true

24: Phospho-(Ser/Thr) Phe Antibody
https://www.cellsignal.com/products/primary-antibodies/phospho-ser-thr-phe-antibody/9631?_=1653338611682&Ntt=9631&tahead=true

25: Phospho-Tyrosine (P-Tyr-1000) MultiMab™ Rabbit mAb mix
https://www.cellsignal.com/products/primary-antibodies/phospho-tyrosine-p-tyr-1000-multimab-rabbit-mab-mix/8954?_=1653338662166&Ntt=8954&tahead=true

26: p75NTR (D4B3) XP® Rabbit mAb
https://www.cellsignal.com/products/primary-antibodies/p75ntr-d4b3-xp-rabbit-mab/8238?_=1653338713820&Ntt=8238&tahead=true

27: PDK1 Antibody
https://www.cellsignal.com/products/primary-antibodies/pdk1-antibody/3062?_=1653338807048&Ntt=3062&tahead=true

28: PP2A A Subunit (81G5) Rabbit mAb
https://www.cellsignal.com/products/primary-antibodies/pp2a-a-subunit-81g5-rabbit-mab/2041?_=1653338854993&Ntt=2041T&tahead=true

29: PP2A B Subunit (100C1) Rabbit mAb
https://www.cellsignal.com/products/primary-antibodies/pp2a-b-subunit-100c1-rabbit-mab/2290?_=1653338892340&Ntt=2290T&tahead=true

30: PP2A C Subunit (52F8) Rabbit mAb
https://www.cellsignal.com/products/primary-antibodies/pp2a-c-subunit-52f8-rabbit-mab/2259?site-search-type=Products&N=4294956287&Ntt=2259t&fromPage=plp&_requestid=876465

31: PTEN (D4.3) XP® Rabbit mAb
https://www.cellsignal.com/products/primary-antibodies/pten-d4-3-xp-rabbit-mab/9188?_=1653338998817&Ntt=9188&tahead=true

32: Rictor (D16H9) Rabbit mAb
https://www.cellsignal.com/products/primary-antibodies/rictor-d16h9-rabbit-mab/9476?_=1653339037996&Ntt=9476&tahead=true

33: Phospho-Rictor (Thr1135) (D30A3) Rabbit mAb
https://www.cellsignal.com/products/primary-antibodies/phospho-rictor-thr1135-d30a3-rabbit-mab/3806?_=1653339073342&Ntt=3806&tahead=true

34: ZEB1 (E2G6Y) XP® Rabbit mAb
https://www.cellsignal.com/products/primary-antibodies/zeb1-e2g6y-xp-rabbit-mab/70512?_=1653339132306&Ntt=70512&tahead=true

35: Snail (C15D3) Rabbit mAb (
https://www.cellsignal.com/products/primary-antibodies/snail-c15d3-rabbit-mab/3879?_=1653339178121&Ntt=3879&tahead=true

36: TWIST1 (E7E2G) Rabbit mAb (
https://www.cellsignal.com/products/primary-antibodies/twist1-e7e2g-rabbit-mab/69366?_=1653339225403&Ntt=69366&tahead=true

37: Sin1 (D7G1A) Rabbit mAb
https://www.cellsignal.com/products/primary-antibodies/sin1-d7g1a-rabbit-mab/12860?_=1653339262450&Ntt=12860&tahead=true

38: Anti-rabbit IgG, HRP-linked Antibody
https://www.cellsignal.com/products/secondary-antibodies/anti-rabbit-igg-hrp-linked-antibody/7074?_=1653339331179&Ntt=7074&tahead=true

From Santa Cruz Biotechnology:
39: Anti-PRMT5 Antibody (A-11)
https://www.scbt.com/p/prmt5-antibody-a-11?requestFrom=search

40: mouse anti-rabbit IgG-HRP
https://www.scbt.com/p/mouse-anti-rabbit-igg-hrp?requestFrom=search

41: m-IgGκ BP-HRP
https://www.scbt.com/p/m-igg-kappa-bp-hrp?requestFrom=search

From Sigma:
42: Anti-dimethyl-Arginine Antibody, symmetric (SYM10)
https://www.sigmaaldrich.com/US/en/product/mm/07-412

43: ANTI-FLAG® antibody produced in rabbit
https://www.sigmaaldrich.com/US/en/product/sigma/f7425

44: Anti-HA-Peroxidase, High Affinity
https://www.sigmaaldrich.com/US/en/product/roche/12013819001

From ThermoFisher
45: Goat anti-Rabbit IgG (H+L) Highly Cross-Adsorbed Secondary Antibody, Alexa Fluor™ Plus 488
https://www.thermofisher.com/antibody/product/Goat-anti-Rabbit-IgG-H-L-Highly-Cross-Adsorbed-Secondary-Antibody-Polyclonal/A32731

46: Goat anti-Mouse IgG (H+L) Highly Cross-Adsorbed Secondary Antibody, Alexa Fluor™ 594

https://www.thermofisher.com/antibody/product/Goat-anti-Mouse-IgG-H-L-Highly-Cross-Adsorbed-Secondary-Antibody-Polyclonal/A-11032

From Bethyl Laboratories
47: Anti-PRMT9 antibody (#A304-189A, 1:1000)
https://www.bethyl.com/product/A304-189A?referrer=search

Eukaryotic cell lines

Policy information about cell lines

Cell line source(s)	CHLA20 and SK-N-BE(2) were obtained from Children Cancer Repository. NGP was purchased from DSMZ.
Authentication	We established a cell bank of all cell lines and sub clones to ensure reproducibility over time. We validate all original cell lines by chromosomal short tandem repeat (STR) profiling. All sub clones will also be validated by STR at least every 6 months. Frozen stocks of all cell line and sub clones are backed up immediately upon generation and validation.
Mycoplasma contamination	All cell lines are routinely tested for mycoplasma contamination every three months when in culture. All the cultured cells used in experiments were tested negative.
Commonly misidentified lines (See ICLAC register)	No commonly misidentfied lines were used in this study.

Animals and other organisms

Policy information about studies involving animals; ARRIVE guidelines recommended for reporting animal research

Laboratory animals	In this study, we used NOD.Cg-Prkdc<scid>Il2rg<tm1Wjl/SzJ (NSG) mice, both male and female, between the age of 8 to 12 weeks. All animals were housed in a 12-h-light/12-h-dark cycle at a temperature-controlled (23 °C ± 0.9 °C) facility with free access to food and water.
Wild animals	This study did not involve wild animals.
Field-collected samples	This study did not involve field-collected samples.
Ethics oversight	All procedures were approved by the Institutional Animal Care and Utilization Committee (IACUC) at UMass Medical School.

Note that full information on the approval of the study protocol must also be provided in the manuscript.

Clinical data

Policy information about clinical studies
All manuscripts should comply with the ICMJEguidelines for publication of clinical research and a completedCONSORT checklist must be included with all submissions.

Clinical trial registration	Clinical data for analysis of the correlation between PRMT5 expression and patient survival were publicaly available from<br>1)Therapeutically Applicable Research to Generate Effective Treatments (TARGET)<br>2)Genomics Analysis and Visualization Platform (R2)
Study protocol	*Note where the full trial protocol can be accessed OR if not available, explain why.*
Data collection	*Describe the settings and locales of data collection, noting the time periods of recruitment and data collection.*
Outcomes	*Describe how you pre-defined primary and secondary outcome measures and how you assessed these measures.*

Flow Cytometry

Plots

Confirm that:

- [x] The axis labels state the marker and fluorochrome used (e.g. CD4-FITC).
- [x] The axis scales are clearly visible. Include numbers along axes only for bottom left plot of group (a 'group' is an analysis of identical markers).
- [x] All plots are contour plots with outliers or pseudocolor plots.
- [x] A numerical value for number of cells or percentage (with statistics) is provided.

Methodology

Sample preparation	Liver, lung, kidney were disrupted using gentleMACS Dissociator
Instrument	Data was acquired on a BD LSR II or MACSQuant 10
Software	Flowjo 10.7.1 or MACSQuantify 2.13.3
Cell population abundance	Cell populations were not sorted on a cell sorter
Gating strategy	For all experiments, single live cells were gated on by forward and side scatter profiles. Live iRF720+ metastatic tumor cells were gated for fluorescent in the AF700 channel.

☐ Tick this box to confirm that a figure exemplifying the gating strategy is provided in the Supplementary Information.
